# Assessing plasmon-induced reactions by a combined quantum chemical-quantum/classical hybrid approach†

**DOI:** 10.1039/d4nr02099e

**Published:** 2024-07-23

**Authors:** Sadaf Ehtesabi, Martin Richter, Stephan Kupfer, Stefanie Gräfe

**Affiliations:** a Institute of Physical Chemistry and Abbe Center of Photonics, Friedrich Schiller University Jena 07743 Jena Germany stephan.kupfer@uni-jena.de s.graefe@uni-jena.de; b Fraunhofer Institute for Applied Optics and Precision Engineering 07745 Jena Germany

## Abstract

Plasmon-driven reactions on metal nanoparticles feature rich and complex mechanistic contributions, involving a manifold of electronic states, near-field enhancement, and heat, among others. Although localized surface plasmon resonances are believed to initiate these reactions, the complex reactivity demands deeper exploration. This computational study investigates factors influencing chemical processes on plasmonic nanoparticles, exemplified by protonation of 4-mercaptopyridine (4-MPY) on silver nanoparticles. We examine the impact of molecular binding modes and molecule-molecule interactions on the nanoparticle's surface, near-field electromagnetic effects, and charge-transfer phenomena. Two proton sources were considered at ambient conditions, molecular hydrogen and water. Our findings reveal that the substrate's binding mode significantly affects not only the energy barriers governing the thermodynamics and kinetics of the reaction but also determine the directionality of light-driven charge-transfer at the 4-MPY-Ag interface, pivotal in the chemical contribution involved in the reaction mechanism. In addition, significant field enhancement surrounding the adsorbed molecule is observed (eletromagnetic contribution) which was found insufficient to modify the ground state thermodynamics. Instead, it initiates and amplifies light-driven charge-transfer and thus modulates the excited states’ reactivity in the plasmonic-molecular hybrid system. This research elucidates protonation mechanisms on silver surfaces, highlighting the role of molecular-surface and molecule-molecule-surface orientation in plasmon-catalysis.

## Abstract 2

This study analyzes plasmon reactions on silver nanoparticles, focusing on 4-MPY protonation. It examines molecular orientation effects on kinetics and thermodynamics in varying electromagnetic fields, emphasizing key charge-transfer processes.

## Graphical abstract

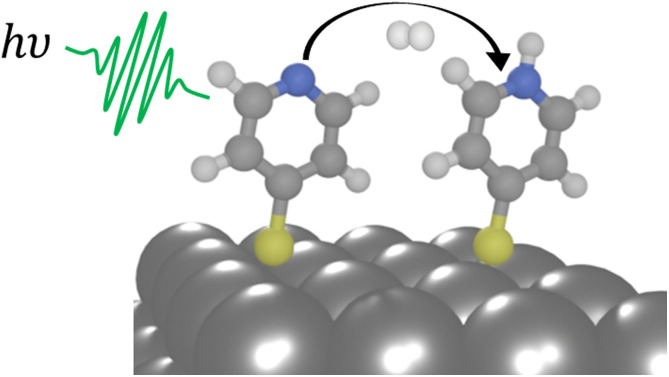

## 1. Introduction

In recent decades, plasmons have found their way into the field of molecular spectroscopy as well as catalysis. The highly confined and enhanced electric field in the vicinity of the plasmonic nanoparticle is the foundation for surface-enhanced Raman spectroscopy (SERS) and tip-enhanced Raman spectroscopy (TERS) – powerful techniques applied in a wide array of fields, ranging from ultrasensitive chemical sensing,^1–6^ single molecule detection^7–12^ and intracellular imaging^13–16^ as well as driving and tracing chemical reactions at the surface of the metallic nanoparticle (plasmon-catalysis).^17–22^ Plasmon-catalysis has been widely investigated experimentally and theoretically, including very different reactions, such as oxidation,^23–32^ reduction,^33–42^ coupling,^43–48^ and dissociation^49–51^ on various metallic nanoparticles. These underlying mechanisms driving plasmon-catalysis can be assigned to three basic categories: thermal contribution/heat, field-enhancement, and charge-transfer processes.^21^ Many previous works have focused on elucidating individual mechanistic contributions in plasmon-driven reactions, demonstrating how each may contribute to the observed outcomes.^52–62^ Among these, Zhang *et al.* emphasized the distinction between thermal and nonthermal effects in plasmon-enhanced catalysis, highlighting the role of hot carriers in accelerating chemical reactions.^56^ Zhao *et al.* proposed two distinct mechanisms: plasmon-driven photocatalysis and plasmon-assisted surface catalysis, offering a framework for understanding how plasmonic nanoparticles influence catalytic reactions through different pathways.^59^ Additionally, Keller and Frontiera addressed the contribution of localized temperature increases, stressing the importance of determining how these temperature effects impact reaction outcomes.^60^ Despite these advances, addressing and contrasting multiple contributions such as heat, light, and electronic structure and dynamics simultaneously remains a challenge.

Examining the injection of energetic charge carriers, including hot electrons and holes, into adsorbed molecules on metal nanoparticles stands as one of the primary mechanisms governing these reactions, and advanced computational methods have been developed and applied.^63–67^ The characterization of adsorbate frontier states and their energies relative to the metal's Fermi level has been accomplished using density functional theory (DFT).^68–73^ This approach allows for an understanding of the electronic structure and energetics of the hybrid systems involved in the charge-transfer processes. Additionally, the exploration of excited states in these hybrid systems has been facilitated through methods such as density functional embedding theory or embedded correlated wavefunction theory.^74–76^ On the other hand, thermodynamic and kinetic investigation of plasmonic reactions allowed to address thermal effects. Furthermore, researchers have endeavored to identify reaction pathways, transition states, and energy barriers, offering a comprehensive view of the reaction mechanisms under plasmonic conditions.^59,74,77,78^

Despite the increased knowledge regarding the mechanisms governing plasmon-catalysis reactions, the pursuit to disentangle the three mechanistic contributions, “hot electrons”, photothermal effects, and electronic excitation of the adsorbate-molecule hybrid system, and to gain a deeper understanding of these remains ongoing. A conscientious and well-balanced evaluation of all aspects associated with these three mechanisms in a single investigation is almost impossible. Therefore, we focus our present theoretical investigation on the electronic ground and excited states properties of the adsorbate-molecule interface and subsequent chemical processes. For this, we introduce a holistic approach based on state-of-the-art quantum chemical simulations in combination with quantum/classical hybrid methods. Exemplary, we explore the protonation reaction of 4-mercaptopyridine (4-MPY) on a silver surface, to benchmark the computational approach and determine factors that influence the light-driven charge-transfer and the thermodynamics of plasmon-driven reactions. The choice is motivated by recent experiments in the group of Volker Deckert:^79^ they found that the protonation of 4-MPY is highly sensitive to environmental conditions and the excitation wavelength. The absence of protonation under continuous flow of argon suggests the necessity on atmospheric molecules as proton source. It has been proposed that H_2_ and H_2_O, present under ambient conditions, may serve as potential sources for the protonation; however, the responsible molecule remained undetermined. Furthermore, the exclusive occurrence of this reaction under 532 nm laser radiation, as opposed to 632 nm irradiation, remained unclear and warrants further investigation.

In this fully theoretical contribution, we follow and extend our computational methodologies as established in several recent joint spectroscopic-theoretical studies in the frame of plasmonic-molecular hybrid systems.^17,18,80,81^ The subsequent section focuses on the analysis of newly generated electronic hybrid states. Thereby, the nature of such states is evaluated for the first time not only by computationally efficient DFT and time-dependent DFT (TDDFT) simulations but also by multiconfigurational wavefunction-based methods that allow an elaborate description of both static and dynamic electron correlation. Furthermore, substrate-surface interactions, field-enhancement effects, charge-transfer processes, and the thermodynamics of the reaction are elucidated. We identify various potential reaction pathways and examine their associated energy barriers while also considering different molecular orientations and varying sources of protons.

## 2. Computational methods

### 2.1 Quantum simulations of isolated and silver-attached 4-MPY

Initially, we have benchmarked both the isolated 4-MPY molecule and the 4-MPY-Ag complex. For a reliable description of these (model) systems, 4-MPY and 4-MPY-Ag, the structures were optimized at the DFT level of theory using the ORCA 4.1.0 package.^82,83^ Subsequently, Complete active space self-consistent field (CASSCF) calculations were conducted within the fully relaxed equilibrium structures. An active space of (12,9) was used for assessing the 14 lowest singlet excited states of 4-MPY. This active space comprised three pairs π_py_/π_py_* orbitals (six electrons), the three lone pairs of the nitrogen and the sulfur atoms (six electrons), providing a robust description of dipole-allowed transitions involving the π-system of 4-MPY as well as of dipole-forbidden nπ* transitions. For 4-MPY-Ag, the 5s orbital of the silver atom was further incorporated, yielding in consequence an (12,10) active space; see Table S1.† This way, charge-transfer states between the molecule and the silver atom are covered additionally. Following the CASSCF calculations, which primarily account for static correlation effects, n-electron valence second-order perturbation theory (NEVPT2) simulations were performed to correct for the lack of dynamical electron correlation. The impact of basis sets on 4-MPY was rigorously evaluated using def2-svp, def2-tzvp, and def2-qzvp basis sets.^84^ With respect to def2-svp, an average shift of −0.61 and −0.73 Eh was observed in the ground and excited states when using def2-tzvp and def2-qzvp, respectively. Since these shifts were relatively consistent across all states, it was determined that the use of the def2-svp basis set is sufficiently flexible for investigating the excited states in the molecular systems of interest. Additionally, more cost-efficient TDDFT calculations were carried out to explore the properties of the lowest 14 excited states of 4-MPY and 4-MPY-Ag. These TDDFT calculations employed the def2-svp^84,85^ basis set and the CAM-B3LYP functional. Although a single silver atom is surely insufficient to fully account for the electronic structure of a metallic nanoparticle, the multiconfigurational simulations allow to benchmark results of the cost-efficient (TD)DFT setup and to extrapolate its applicability for the more sophisticated hybrid system studied herein.

### 2.2 Quantum simulations of 4-MPY on silver surface

These simulations enable examining 4-MPY's modified chemical properties caused by the vicinity of the Ag surface that modulate the ground and excited state electronic structure (*i.e.* locally excited and charge-transfer states). All geometry optimizations were carried out using DFT calculations based on the projector-augmented wave (PAW) method, incorporating the optB88-vdW functional.^86^ The calculations employed a real-space grid with a resolution of 0.2 Å, implemented in the GPAW program package in cooperation with ASE interface.^87–90^ The Ag slab is represented by a 4 × 4 × 3 fcc(111) cluster, resulting in 3 layers of 16 atoms using an optimized lattice constant of 4.19 Å. A robust description of the surface-sample interactions requires a sufficient number of silver layers to be included. Adsorption energies (*E*_ads_ = *E*_mol+slab_ − *E*_mol_ − *E*_slab_) were calculated for the hybrid system with one to 15 layers; convergence was achieved upon three Ag layers (see Fig. S1†). The structural relaxation was performed employing two-dimensional periodic boundary conditions (*x*- and *y*-direction) while the second and the third layers of the Ag slab were kept frozen to reduce computational costs.

We studied three configurations of the respective hybrid systems in depth. While chemisorption is realized in all three configurations by the sulfur-silver interaction, the orientation of the aromatic plane with respect to the silver surface varies, and thus, the additional physisorption. All optimized structures are available from the online repository Zenodo *via* ref. 91. In the first orientation, denoted as structure 1, the aromatic plane of the molecule is orthogonal to the Ag surface. Structure 2 displays a slight non-perpendicular deflection based the interaction between the ^2^C's hydrogen atom with the silver surface (see Fig. S2† for atom labels). Finally, in structure 3, the substrate is aligned to lie flat on the surface, and the aromatic ring is almost parallel to the Ag surface. Here, the molecule binds to the surface through interactions of the silver surface with the S atom as well as with the π orbitals of the ring. We note that structures 1 and 2 represent orientations enabling a large surface coverage of adsorbed molecules on a Ag surface; in contrast, structure 3 describes a possible scenario with small surface coverage.

To identify reaction pathways and their associated energy barriers, the automated nudged elastic band algorithm (AutoNEB) in conjunction with the climbing image method was used; the image-dependent pair potential (IDPP) interpolation method was applied for improving the initial guess of the NEB path.^92–96^

Subsequently, we proceeded with non-periodic TDDFT simulations, utilizing the Gaussian 16 program.^97^ These simulations were focused on the hybrid systems incorporating additionally H_2_ as a proton source and were conducted at the CAM-B3LYP/def2-svp level of theory.^84,85,98^ Our objective was to gain a comprehensive understanding of the electronic transitions associated with light-driven processes induced by resonant photoexcitation at either 633 nm *vs.* 532 nm in the plasmonic hybrid systems. This involved the characterization of electronic transitions, encompassing both local substrate excitation and charge-transfer excitations between the substrate and the metal cluster, as assessed through charge density differences (CDDs). Detailed results are provided in the ESI (see Tables S2–S6†).

### 2.3 Hybrid quantum/classical simulations

To study dynamical interactions between 4-MPY and silver nanoparticle as well as to assess the near-field enhancement, we employed a hybrid quantum/classical method. This method combines the quasistatic finite-difference time-domain (QSFDTD) and time-propagation TDDFT,^99–101^ implemented in the GPAW program package in cooperation with ASE interface.^87–90^ The whole system was divided into a quantum and a classical subsystem, allowing to investigate, both the chemical as well as the electromagnetic contributions. The classical subsystem, a Ag-sphere nanoparticle with a radius of 60 Å, is treated by the QSFDTD method in its own real-space grid with a resolution of 3 Å. The permittivity used for silver was taken from ref. 101. The quantum subsystem containing the 4-MPY molecule adsorbed on the silver cluster was propagated using the time-propagation TDDFT method on a real-space grid of 1/3 Å grid spacing which fit completely inside the classical grid. Each subsystem was propagated independently on its own real-space grid, yet they shared a common electrostatic potential. The time evolution was followed in these calculations for 24 fs employing a time step of 12 as.

## 3. Results and discussion

### 3.1 Isolated and silver-attached 4-MPY

We investigated the electronic structure of 4-MPY in gas phase as well as surface-immobilized on silver (4-MPY-Ag) with the help of quantum chemical simulations both at the multiconfigurational and at the (time-dependent) DFT levels of theory. As described above, the silver surface was mimicked initially exclusively by a single silver atom attached to the sulfur atom. This molecular model system allows benchmarking the resource-efficient TDDFT simulations with respect to accurate multiconfigurational.

The CASSCF/NEVPT2 simulations show that the isolated 4-MPY molecule does not feature any electronic excited states within the range of the laser radiation. This highlights that it is essential to account for the emergence of newly generated hybrid excited states, a result of the molecule-metal interactions. Already for the simple case, 4-MPY-Ag, the NEVPT2 calculations have affirmed the presence of an excited electronic state at 2.38 eV (520 nm), arising from the interaction between the metal and the molecule. Notably, this state coincides with the wavelength of the incident laser excitation of 532 nm used in the experiment.^79^

This finding suggests that this wavelength is not only in resonance with the silver nanoparticles but also precisely matches the energy needed to excite the hybrid state of the system and to the promote the population of charge-separated states.

Consequently, this result emphasizes that the energy of metal nanoparticle's LSPRs and the often-overlooked hybrid states during the excitation need to be considered and matched to promote a certain (redox) reaction of interest along the sample-molecule interface.

Moreover, as illustrated in Table 1, the TDDFT results, obtained using the range-separated CAM-B3LYP functional, show good agreement with the NEVPT2 results with respect to excitation energies and electronic charters. In all cases, the energetic deviation is below 0.2 eV for locally excited states of 4-MPY as well as for charge-transfer transitions from the substrate to the semi-occupied 5s orbital of the silver. These charge-transfer states cause the oxidation of 4-MPY and reduce the silver atom, which mimics a silver nanoparticle. This consistency of the high level multiconfigurational results with the TDDFT values confirms the suitability of the CAM-B3LYP functional to describe the ground and excited states in such hybrid systems.

**Table 1:** Comparison of key excited transitions of 4-MPY and 4-MPY-Ag as obtained by CASSCF/NEVPT2 and TDDFT (CAM-B3LYP)

	Excited state	NEVPT2 energy [eV]	TDDFT energy [eV]	Electronic transition (weight %)		
4-MPY	ππ*	5.20	5.37	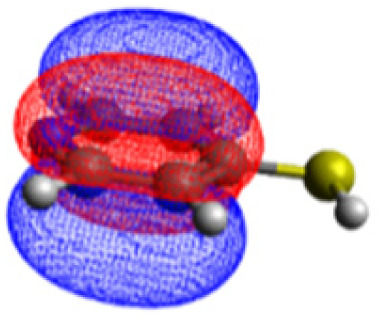	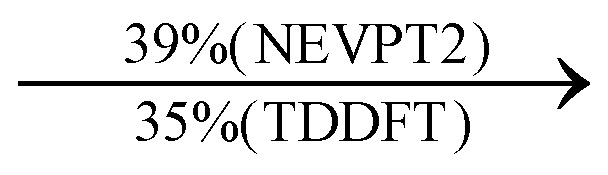	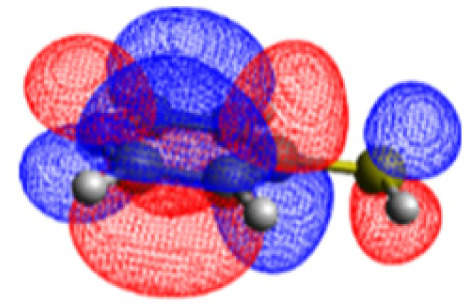
				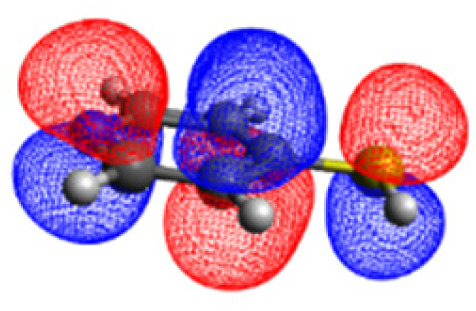	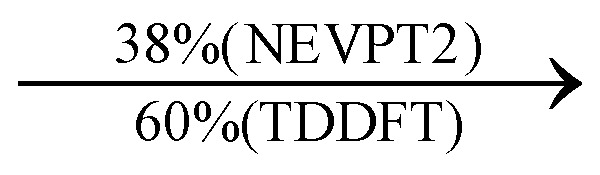	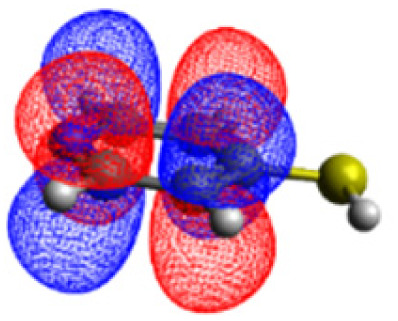
	nπ*	5.32	5.19	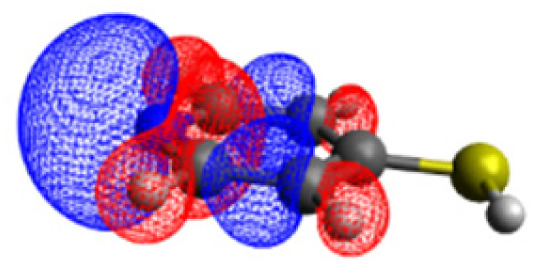	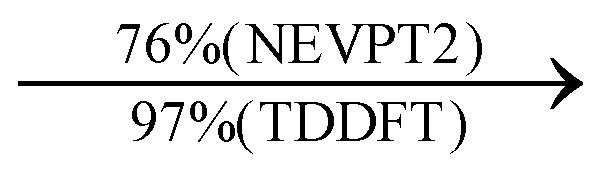	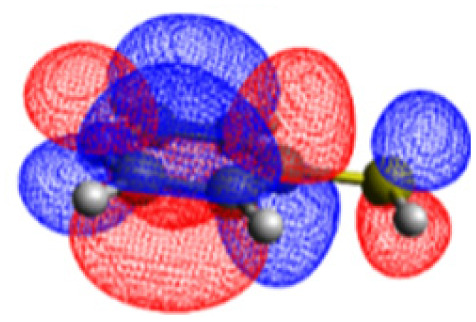
4-MPY-Ag	LMCT_1_	2.38	2.29	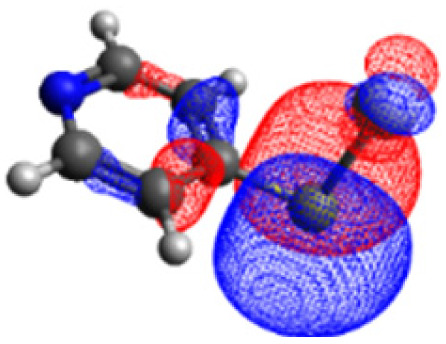	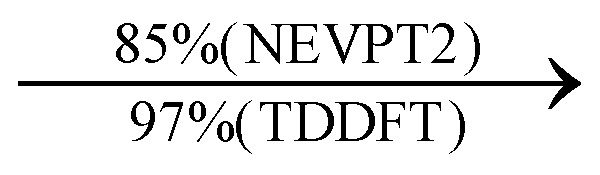	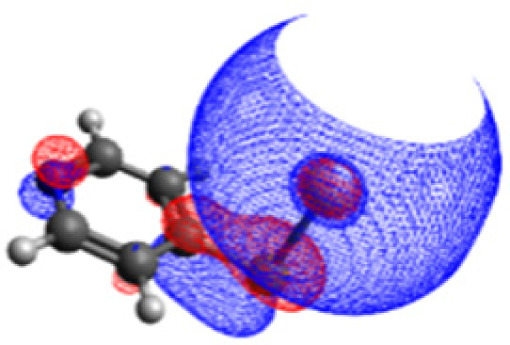
	LMCT_2_	2.90	3.02	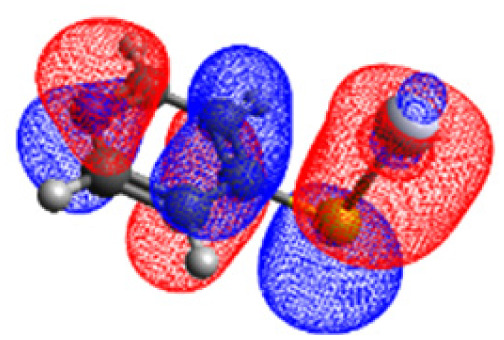	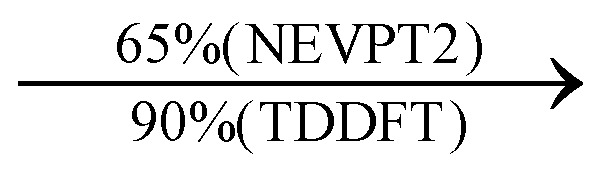	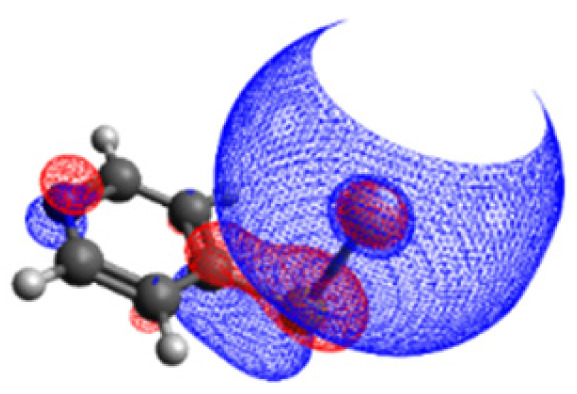

### 3.2 Effects of molecular binding modes and proton sources on thermodynamic properties of reaction

**Fig. 1:**
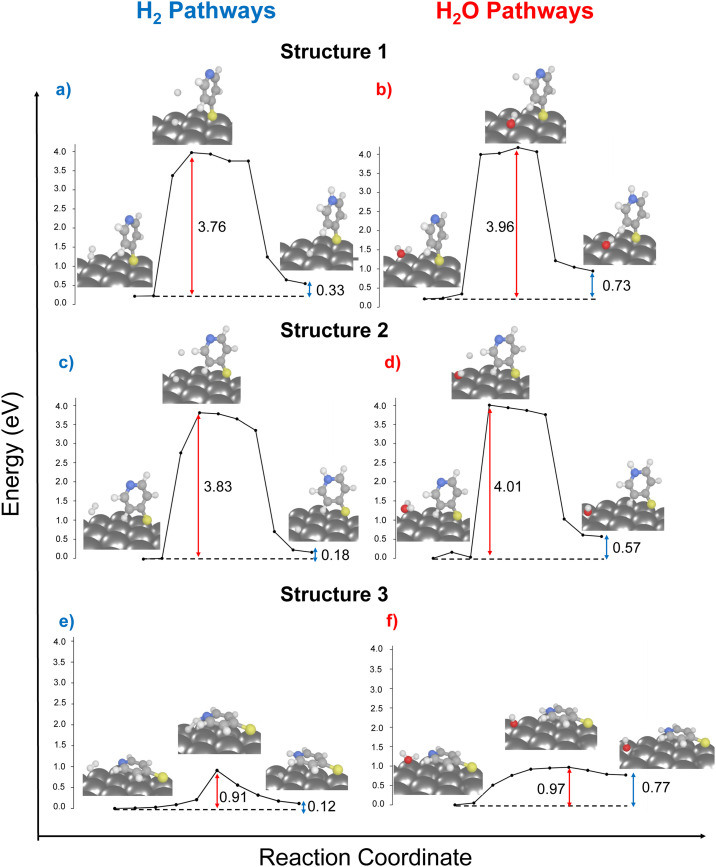
Ground-state protonation pathways of 4-MPY on a silver surface: six diagrams (a–f) depicting different molecular orientations (structures 1, 2, and 3) and varied proton sources (hydrogen and water, left and right). The initial (reactant), transition and final (product) states are illustrated for each pathway. The energy barriers (red arrow) and reaction energies (blue arrow) are shown.

A chemical reaction is characterized by both thermodynamic and kinetic aspects. Specifically, to determine thermodynamic properties, we assess reaction energy while for the kinetic aspects, we calculate the reaction's activation energy. For reliable values, the description within a single silver atom is insufficient. In addition, various orientations and binding modes of the molecule on the silver surface need to be considered. As described above, three configurations are investigated in detail. Thereby, all structures are chemically bound *via* a S–Ag bond, however, with different orientation of the aromatic ring to the surface. Structures 1 and 2 feature a rather orthogonal orientation with structure 2 being tilted slightly sideways in favour of a hydrogen – silver interaction. Notably, in general such an upright 4-MPY orientation is assumed.^102–104^ Experimentally, the molecular orientation of the surface-immobilized aromatic substrates is typically characterized by means of the prominent ring breathing mode which acts as marker band. However, this ring-breathing mode is polarized within the aromatic plane. Therefore, this marker band is best detectable (*z*-direction) in case of surface-sample configurations with 4-MPY pointing upwards (structures 1 and 2). In contrast, this mode is (almost) Raman inactive (*z*-direction) in case of the flat-lying structure 3. Therefore, as the Raman signal of this marker band is notably less intense in the flat lying configuration (*e.g.* structure 3) in comparison to the vertically aligned orientations, it is thus likely that the fraction of molecules parallel to the surface is experimentally underestimated. Finally, we consider various potential sources of protons at ambient conditions. Considering both hydrogen and water molecules as potential proton sources involved in the plasmon-induced protonation of 4-MPY, we investigated two distinct pathways for each configuration of the hybrid systems, resulting in two pathways for each of the three conformers, for a total of six paths connecting the respective initial and final states (Fig. 1).

**Fig. 2:**
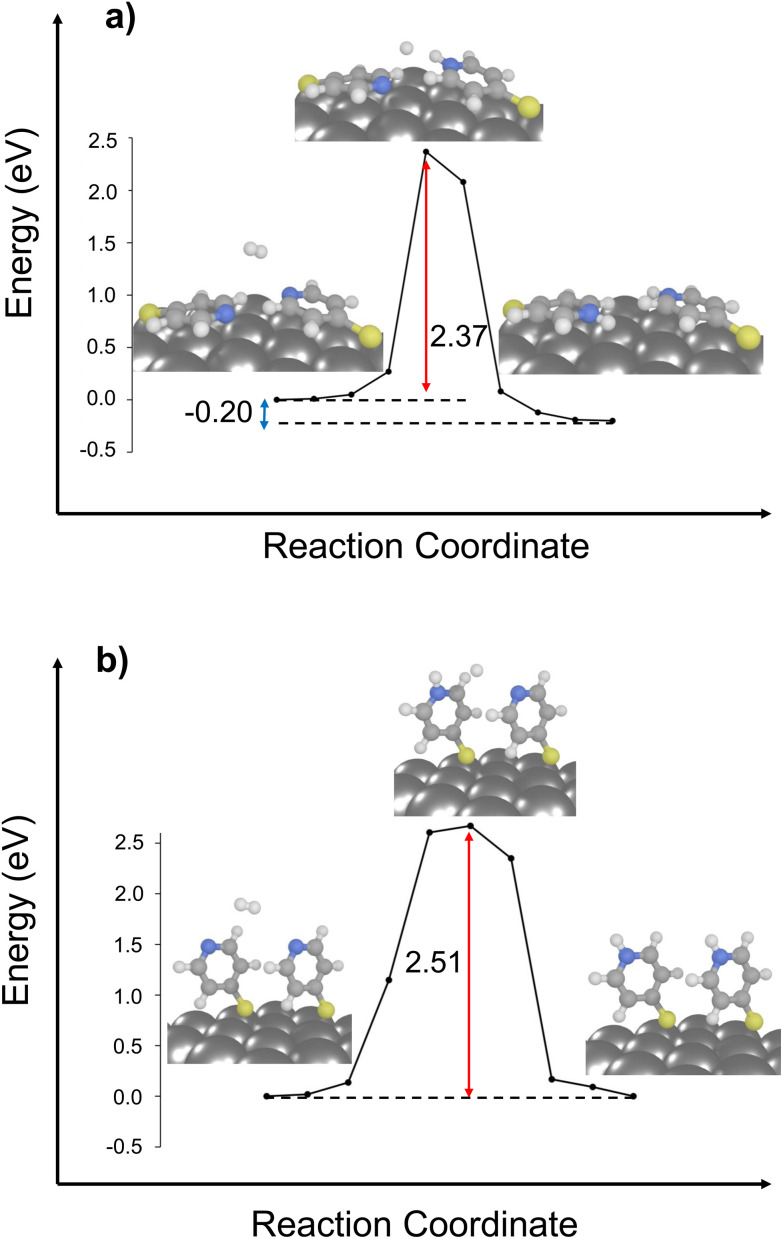
Ground-state protonation pathways of two 4-MPY on a Ag surface in two different molecular orientations, (a) structure 3 and (b) structure 2, considering the hydrogen molecule as proton source. The initial (reactant), transition and final (product) states are illustrated for each pathway. The energy barriers (red arrow) and reaction energy (blue arrow) are shown.

As depicted in Fig. 1, all six considered reaction pathways are endothermic. This indicates already that the protonation of the nitrogen only proceeds under plasmonic conditions, within the excited states of the hybrid system. However, the specific reaction energies vary depending on the molecule's binding mode. Remarkably, pathways involving hydrogen molecules, see Fig. 2(a), (c) and (e), are slightly less thermodynamically unfavorable (*i.e.*, +0.12 to 0.33 eV) compared to those involving water molecules (*i.e.*, +0.57 to 0.77 eV). Among the H_2_-mediated pathways, structure 1 features with 0.33 eV the highest energy demand, structure 2 needs 0.18 eV uphill in energy, and structure 3 notably is the most favorable endothermic by 0.12 eV, making it the most feasible candidate only based on the reaction energy.

Based on the comparison of the energy barriers of the investigated pathways, it becomes evident that both structures 1 and 2 feature considerable energy barriers for hydrogen as well as for water-mediated protonation; Fig. 1(a–d). Activation energies of 3.76 (H_2_) and 3.96 eV (H_2_O), respectively, are predicted in case of structure 1. Similarly, structure 2 features barriers of 3.83 and 4.01 eV, respectively. Notably, these substantial activation energies cannot be overcome by the energy provided by the laser excitation, which operates at either 532 nm (2.33 eV) or 632 nm (1.97 eV).

This underscores the critical importance of the molecule's orientation in initiating the reaction. Therefore, by taking both reaction energy and energy barrier into consideration, the reaction pathway of structure 3, incorporating a H_2_ molecule (Fig. 1(e)), which is endothermic by 0.12 eV and featuring a barrier of 0.91 eV, emerges as the most probable pathway for the protonation reaction. Thus, our calculations reveal that the chemical environment in the vicinity of the N-atom is of fundamental importance to stabilize the transition state's energy, *i.e.*, the second H atom in case of the investigated H_2_ pathway or the OH in the H_2_O pathway. Furthermore, this result underscores the need to carefully consider the effects of surface coverage of self-assembled monolayer on these modes, and to meticulously examine the edges of the nanostructures. Both factors are crucial as they can influence molecular binding modes, thereby affecting reaction outcomes.

### 3.3 Effect of dispersive molecule-molecule interaction on thermodynamic properties of reaction

In the following, we extended our 2D-periodic DFT setup to include two neighboring molecules on the Ag surface in the same unit cell. This extension was exclusively performed for structures 2 and 3 (see Fig. 2). This methodological choice enabled us to systematically investigate the influence dispersive molecule-molecule interactions, thereby mirroring the conditions of high-coverage (structure 2) monolayers *vs.* upon low surface coverage (structure 3). For these calculations, one H_2_ molecule was considered as proton source.

As illustrated in Fig. 2, both reaction pathways exhibit enhanced driving force when compared to scenarios involving only a single molecule on the surface. Particularly in the case of structure 3, which features a favorable driving force of −0.20 eV.

In case of the activation energies for the bi-molecular structures, rather similar barriers of 2.51 and 2.37 eV are predicted for the bi-molecular analogous of structures 2 and 3 (Fig. 2). The chemical environment of the nitrogen atom is further governed by dispersive effects stemming from the second 4-MPY molecular – either by means of the π-system (structure 2) or *via* the lone-pair of the other nitrogen atom (structure 3). Notably, both 4-MPY molecules are getting protonated for the bi-molecular H_2_ pathways. Therefore, the activation energy per 4-MPY molecule is with 1.26 and 1.19 eV quasi-identical. In consequence, our simulations reveal that π-interactions among the surface-immobilized molecules allow to reduce the barrier for the studied H_2_ dissociation substantially (from 3.83 to 1.26 eV), while a second neighbouring N-atom in the bi-molecular structure 3 slightly increases the barrier form 0.91 (mono-molecular case) to 1.19 eV (bi-molecular case). Thus, stabilization of the second H-atom within the transition state is more favourable in case of the Ag slab in comparison to a second N-atom lone-pair in proximity.

The reason for the selective occurrence of the protonation reaction under 532 nm laser radiation, as opposed to 632 nm, remained opened. By considering intermolecular interactions, it became apparent that protonation cannot occur under 632 nm (1.97 eV) irradiation due to the inadequate energy to overcome the energy barrier of 2.37 eV. This results further reveals the impact of surface coverage and its association with intermolecular binding modes and chemical reactivity. In scenarios of low surface coverage or/and at nanoparticle edges, reactions can successfully proceed *via* the ground state, facilitated by a flat-lying binding mode (referenced as structure 3). However, in high coverage samples, the likelihood of adopting a flat-lying binding mode decreases significantly along with its detectability.

### 3.4 Effects of electromagnetic field on thermodynamic properties of reaction

Up to this point, our analysis has focused solely on chemical contributions, omitting the influence of the electromagnetic field. These electromagnetic effects were accounted for by hybrid quantum-classical calculations (see Computational methods section for more details).

**Fig. 3:**
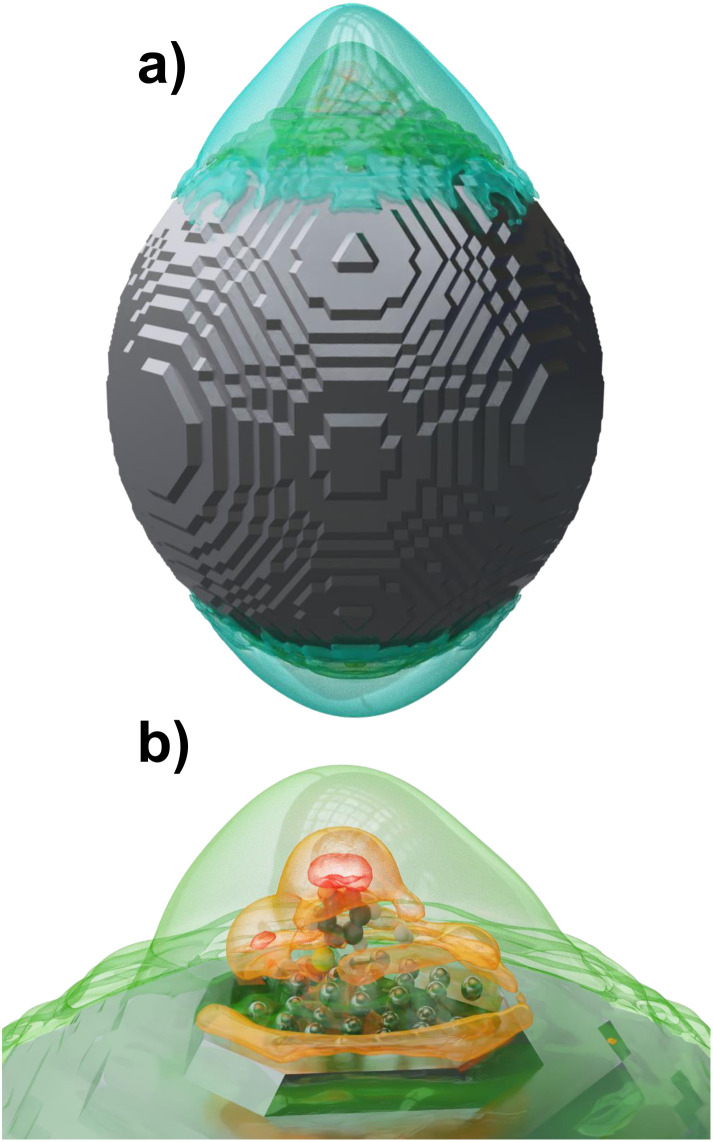
Field enhancement at 532 nm (2.33 eV) is shown with coloured surfaces for a factor of 4 (blue), 5 (green), 7 (orange) and 10 (red) for (a) hybrid quantum-classical system and for (b) quantum subsystem.

Fig. 3 illustrates the field enhancement at an excitation wavelength of 532 nm, which corresponds to the laser's irradiation wavelength used in the experimental setup.^79^ Notably, there is a significant field enhancement in the vicinity of the adsorbed molecule (Fig. 3(b)), suggesting that such enhancement could potentially lower the energy barrier, thereby facilitating the protonation reaction.

**Fig. 4:**
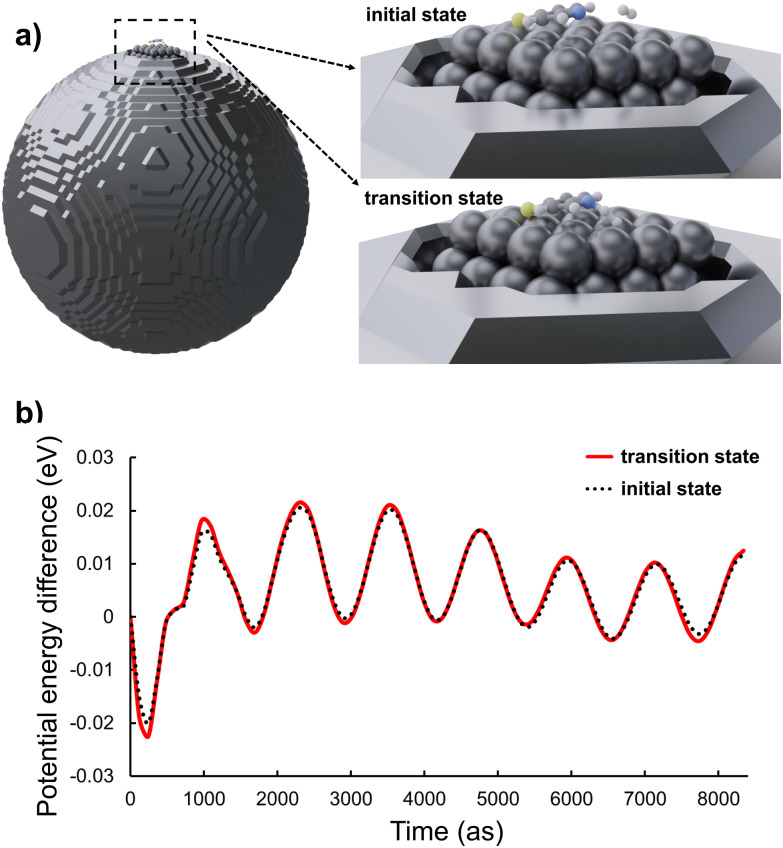
(a) Illustration of the quantum-classical hybrid system, comprising a classical silver nanoparticle with a 120 Å diameter and a quantum subsystem consisting of 48 silver atoms along with reactive compounds (4-MPY and H_2_). (b) Depiction of the energy change within the quantum subsystem over time, influenced by an electromagnetic field, for both the initial and transition structures.

To explore this hypothesis, the electronic structure of the initial state as well as of the transition state and the associated activation energy were evaluated in the presence of a time-dependent electromagnetic field; see Fig. 4(a). The results, as depicted in Fig. 4(b), reveal that the energy gap between the initial and the transition states is quasi time-independent while the energies of both states oscillate with the frequency of the electromagnetic field. Similar results were also observed for other structural configurations, including the case involving two molecules on the surface. For detailed results of hybrid calculations on these structures and regarding the field enhancement related to structure 3, refer to the ESI (Fig. S3 and S4†).

Contrary to expectations that the electromagnetic field decrease the reaction's activation energy, our data reveal no meaningful alteration.

This finding prompted us to further focus our investigation of the reaction mechanism on the chemical effect by calculating charge-transfer states of the hybrid system.

### 3.5 Investigating charge-transfer states and their variability with different molecular binding modes

While it has been already established that charge-transfer excited states influence plasmon-catalysis,^17,18,80^ a stepwise approach was necessary to comprehensively understand these reactions.

To investigate the properties of electronically excited states within the plasmonic hybrid system, we employed non-periodic TDDFT calculations without accounting for the electromagnetic field, computing the lowest 600 electronic excited states for each structure.

These calculated transitions were subject to a comprehensive analysis to elucidate their electronic characteristics, *i.e.*, to elucidate the nature of the dipole-allowed metal-to-molecule charge-transfer, molecule-to-metal charge-transfer, metal-centered and molecule-centered transitions in the vicinity of the excitation energy. In the subsequent discussion, we abstain from discussing specific electronic excitations, primarily due to the plasmonic hybrid system model's complex nature, which features numerous highly mixed and weakly absorbing transitions. This is especially notable in the context of charge-transfer processes involving the Ag slab and the surface-immobilized substrate. Therefore, associating the plasmonic reaction of interest with the populations of a distinct electronic (excited) state or even constructing a reaction coordinate associated with such a state is impossible.

**Fig. 5:**
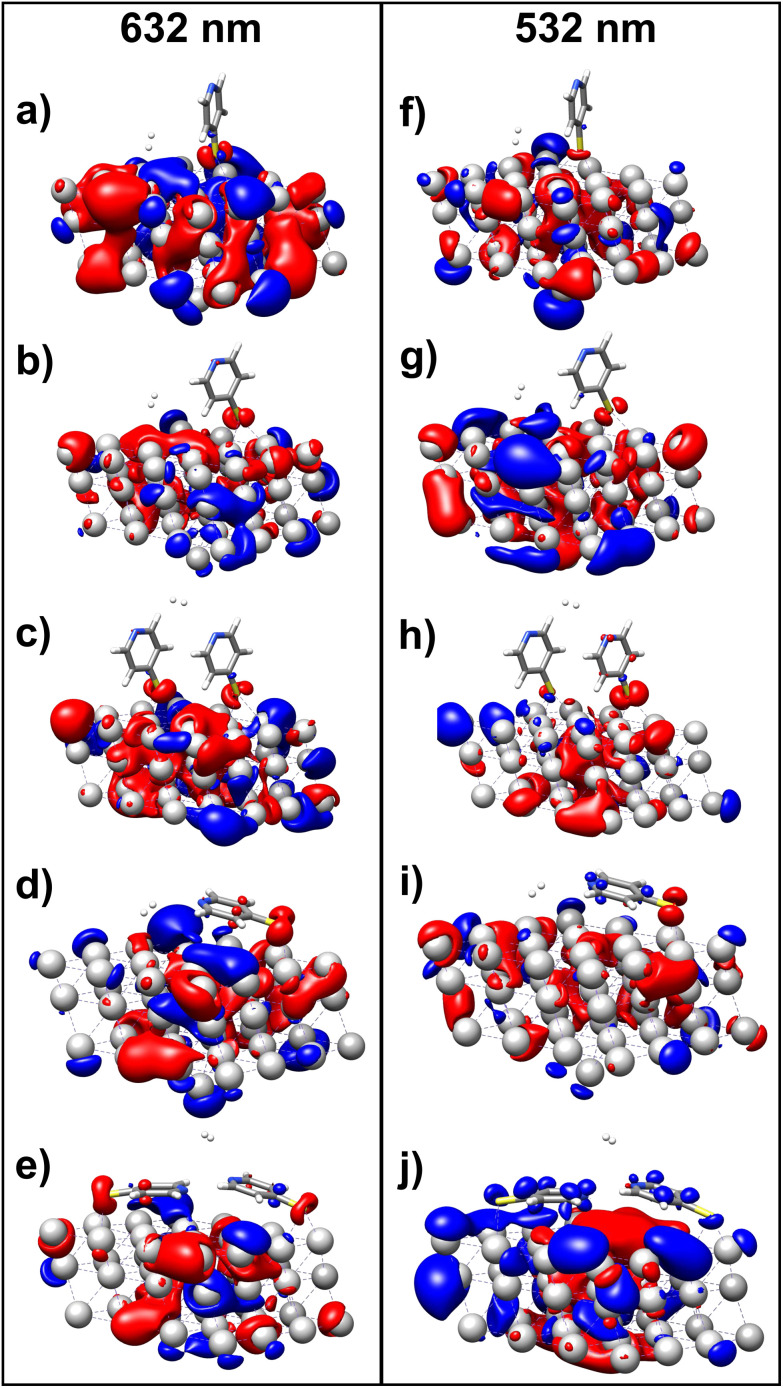
Charge density differences (CDDs) revealing the electronic character of low-lying, dipole-allowed excitations at two distinct wavelengths – 632 nm (a–e) and 532 nm (f–j) – within various orientations of 4-MPY interacting with H_2_. Excitations for structure 1 are visualized in (a) and (f), while structure 2's excitations are showcased in (b) and (g). For structure 2, where two 4-MPY molecules are considered, results are provided in (c) and (h). Structure 3's excitations are illustrated in (d) and (i); for structure 3 with two 4-MPY molecules, the results are displayed in (e) and (j). Charge-transfer takes place from red to blue.

To initiate the protonation of 4-MPY, a fundamental condition is the occurrence of (excited state) charge-transfer from the metal surface to the molecule, specifically directed towards the nitrogen atom, which in consequence may trigger the protonation due to the enhanced site-specific electronic density. As visualized by means of charge density difference (CDD) plots in Fig. 5, the electronic excited states within the 632 nm wavelength range consistently exhibit the characteristics of metal-centered states, irrespective of the molecule's orientation, which translate in a more material interpretation to the plasmonic states of the nanoparticle. This observation implies that, regardless of the molecule's orientation relative to the surface, triggering the protonation reaction under 632 nm laser radiation is unfeasible since the electronic structure of the adsorbed molecule remains unaffected at this excitation wavelength. The situation differs substantially when examining the charge-transfer states within the 532 nm wavelength region. Here, the nature of these states is influenced by the orientation of the molecules. For structures 1 and 2, the electronic transitions are predominantly of metal-centered character (Fig. 5(f)–(h)), similar to what was observed in the range of 632 nm. However, in case of structure 3, the flat-lying orientation, the excited states display a metal-to-molecule character as shown in Fig. 5(i). Therefore, the electron density of the surface-immobilized 4-MPY is higher upon population of such metal-to-molecule charge-transfer states. In consequence, the nitrogen's basicity is increased, which in turn, favors its protonation within the excited state compared to the electronic ground state. This characteristic becomes notably enhanced when considering dispersive molecule-molecule interactions as shown in Fig. 5(j). Notably, the lifetime of such charge-separated excited state is presumably rather short, however, the continuous laser excitation repeatedly pumps such state with the laser frequency. Thus, statistically, the plasmon-induced protonation proceeds in case of a favorable pre-orientation of the proton source in the vicinity of the 4-MPY's nitrogen atom.

Thus, we could show that the investigated protonation reaction of 4-MPY proceeds within the realm of excited states. Unfortunately, the assessment of excited state reaction pathways for this reaction proved unattainable due to the substantial presence of numerous closely mixed excited states. Worth noting is that in case of the single Ag atom employed to mimic the metallic nanoparticle (Table 1), the charge-transfer is predicted in opposing directions – namely from 4-MPY to the Ag atom. This finding highlights the necessary to utilize a sufficiently large model to adequately describe the electronic properties of the metallic nanoparticle.

These findings align remarkably well with experimental results and allow to elucidate the mechanism underlying this reaction, one that depends on various parameters.

## 4. Conclusions

The reaction mechanisms underlying plasmon-induced reactions are still widely unknown. In our present case study, we focused on a rather simple model reaction, namely the plasmon-driven protonation of 4-mercaptopyridine (4-MPY) on silver nanoparticles. In our fully theoretical investigation, we evaluate the thermodynamic properties such as driving forces and activation energy depending on the binding mode as well the reactivity of electronically excited states of the hybrid system while considering both the chemical and the electromagnetic contributions.

We found that the activation energy of the reaction varies considerably based on the binding mode and the proton source, *i.e.*, H_2_*vs.* H_2_O. The sample's binding mode onto the Ag surface plays a vital role in determining the feasibility of the reaction. While energy barriers for some pathways are insurmountable within the range of laser energy, certain configurations, *i.e.*, with the aromatic plane roughly parallel to the Ag (111) surface (structure 3) and considering a H_2_ as proton source, exhibit the most probable pathway for the protonation reaction. Expanding our research to the exploration of dispersive molecule-molecule interactions in scenarios involving two close-lying molecules on the surface, we noted that dispersive molecule-molecule interactions influence the reaction favorability and barriers, providing valuable insights for high-coverage self-assembled monolayers.

Regarding the role of electromagnetic fields in plasmon-catalysis reactions, our findings have shown that the electromagnetic contributions do not significantly alter the activation energy along the ground state pathways. However, the electromagnetic (near-)field plays a pivotal role to trigger charge-transfer processes within the excited states of the hybrid system.

Examining charge-transfer excited states between the metallic nanoparticle and the adsorbed substrate, we observed that protonation initiation under 632 nm laser excitation remains unfeasible. This conclusion is drawn as 632 nm excitation does not allow to alter the electronic structure of the surface-immobilized 4-MPY – neither by a local (ππ*) excitation of the organic substrate nor *via* a charge-transfer process between the nanoparticle and substrate. Notably, contribution of thermal heating and a potential impact of hot electrons were not investigated herein. In contrast, 532 nm excitation drives a charge-transfer from the silver to the molecule's lowest energy π* orbital. This is observed in particular for a flat orientation of 4-MPY on the silver surface. Thus, protonation of such excited (charge-separated) species is more likely to occur due to the increased excited-state basicity of 4-MPY. These results align remarkably well with the experimental observations, providing insights into the mechanism underlying the protonation reaction.

Therefore, our computational protocol based on a combined quantum chemical-quantum/classical hybrid approach allows to assess binding modes, intermolecular interactions, thermodynamical properties as well as excited states of plasmonic hybrid systems. This way, the underlying reaction mechanism in a broad range of plasmon-induced catalytic reactions are assessable based on the protocol established in the present contribution. Furthermore, the present study highlights the importance of charge-transfer processes within the excited states of the plasmonic hybrid system and, thus, of the chemical contribution in plasmon-catalysis.

## Data availability

The data supporting this article have been included in the main manuscript as well as part of the ESI.† Furthermore, all optimized structures are available *via* the free online repository Zenodo (https://doi.org/10.5281/zenodo.10276984); ref. 91.

## Conflicts of interest

There are no conflicts to declare.

## Supplementary Material

NR-016-D4NR02099E-s001
