# Supplementary material for: Assessing plasmon-induced reactions by a combined quantum chemical-quantum/classical hybrid approach
Source: Nanoscale. 2024 Jul 23;16(32):15219–29. doi: 10.1039/d4nr02099e (PMC11325215; doi:10.1039/d4nr02099e)
Supplement: NR-016-D4NR02099E-s001 [file NR-016-D4NR02099E-s001.pdf]

Supplementary Information (SI) for Nanoscale.
This journal is © The Royal Society of Chemistry 2024

Supporting Information

Assessing Plasmon-Induced Reactions by a Combined Quantum Chemical-Quantum/Classical Hybrid Approach

Sadaf Ehtesabi, Martin Richter, Stephan Kupfer, Stefanie Gräfe**

Table S1. Active space orbitals used in CASSCF calculations for 4-MPY and 4-MPY-Ag

4-MPY	4-MPY-Ag
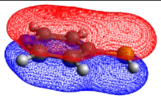	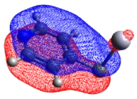
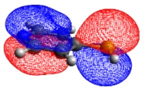	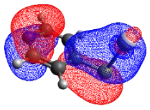
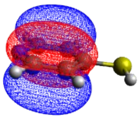	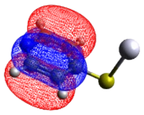
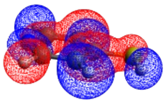	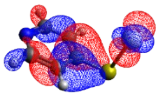
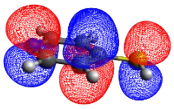	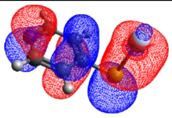
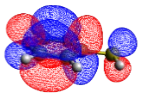	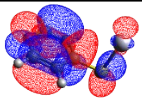
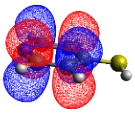	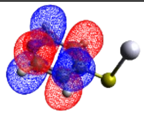
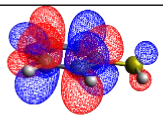	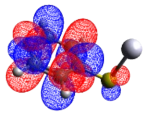
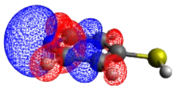	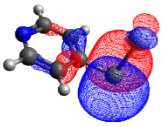
	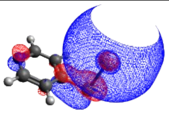

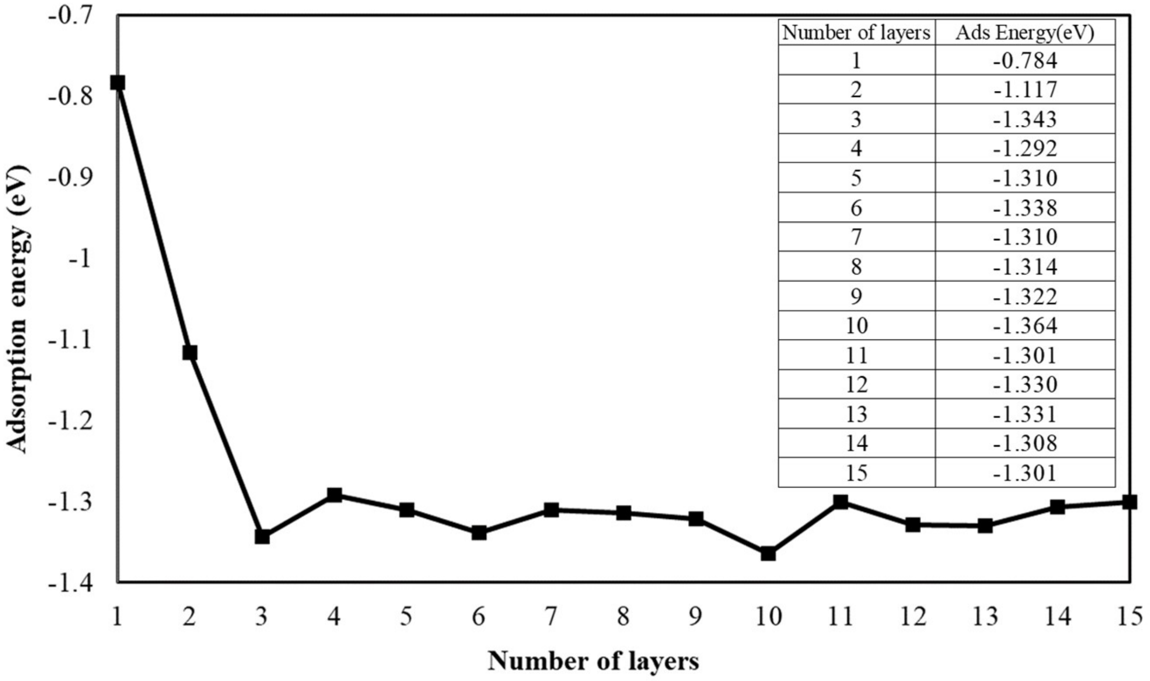

Number of layers	Ads Energy(eV)
1	-0.784
2	-1.117
3	-1.343
4	-1.292
5	-1.310
6	-1.338
7	-1.310
8	-1.314
9	-1.322
10	-1.364
11	-1.301
12	-1.330
13	-1.331
14	-1.308
15	-1.301

Figure S1. Calculated adsorption energy of 4-MPY molecule across silver surfaces ranging from 1 to 15 layers. The data provides critical insights into the optimal number of layers needed for accurate simulation, highlighting the balance between computational efficiency and model precision.

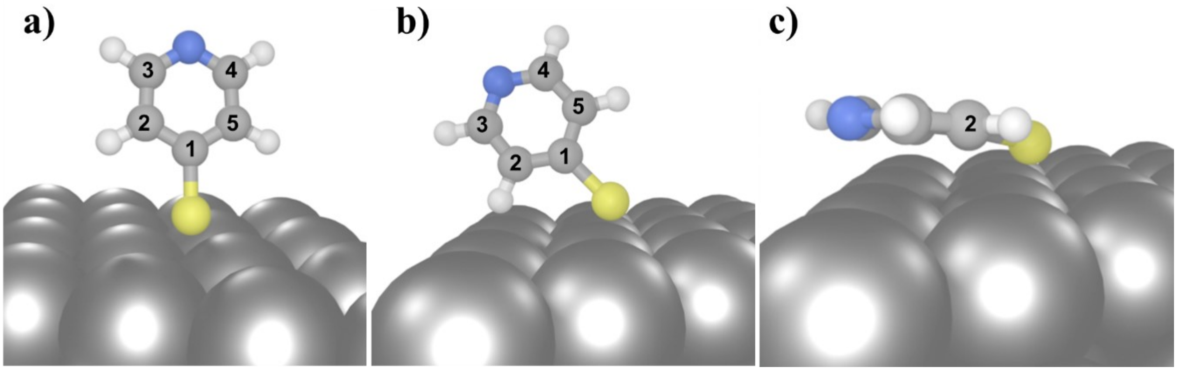

Figure S2. Different optimized structures denoted as (a) structure 1 (b) structure 2 (c) strcuture3

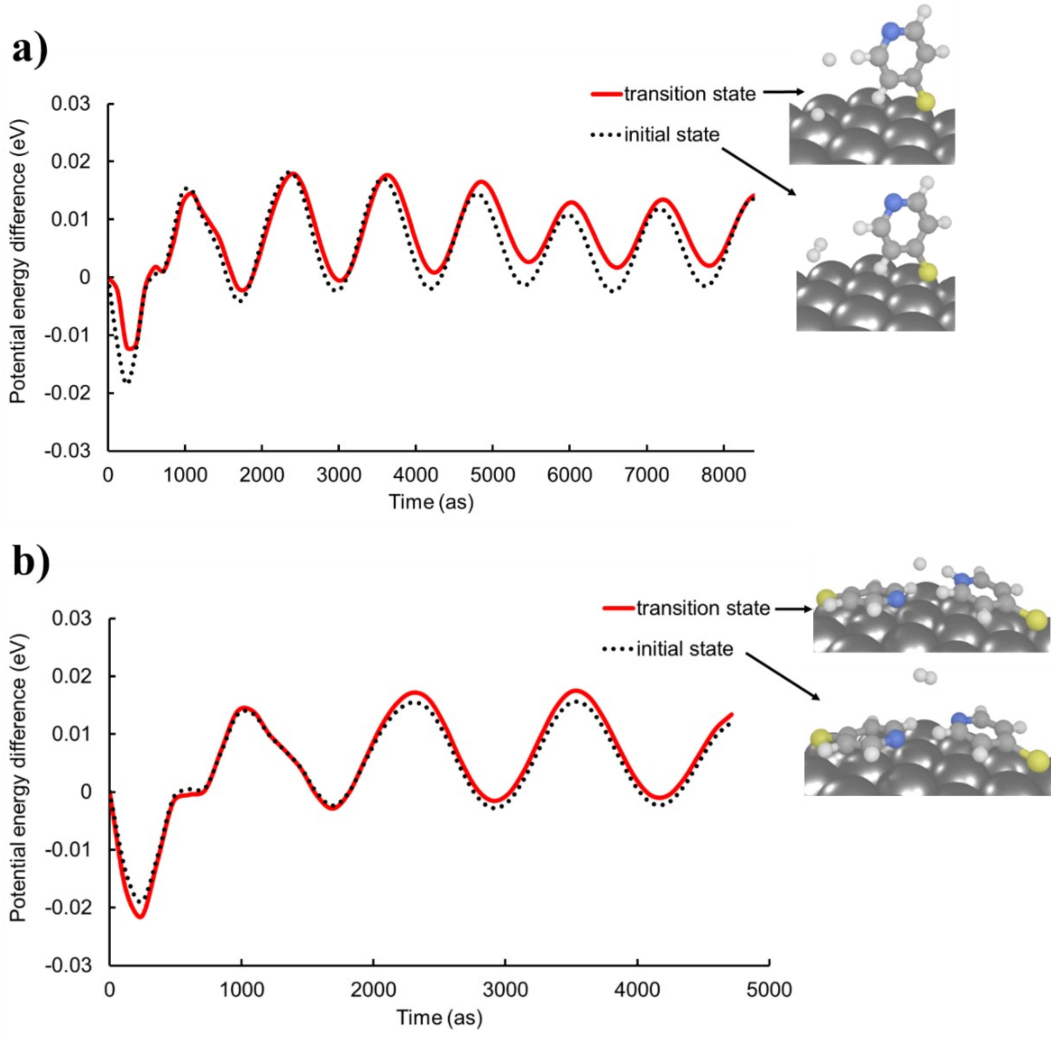

Figure S3. Depiction of the energy change within the quantum subsystem over time, influenced by an electromagnetic field, for both the initial and transition structures (a) in structure 2 and (b) in two molecules in structure 3 considering hydrogen molecule as proton source.

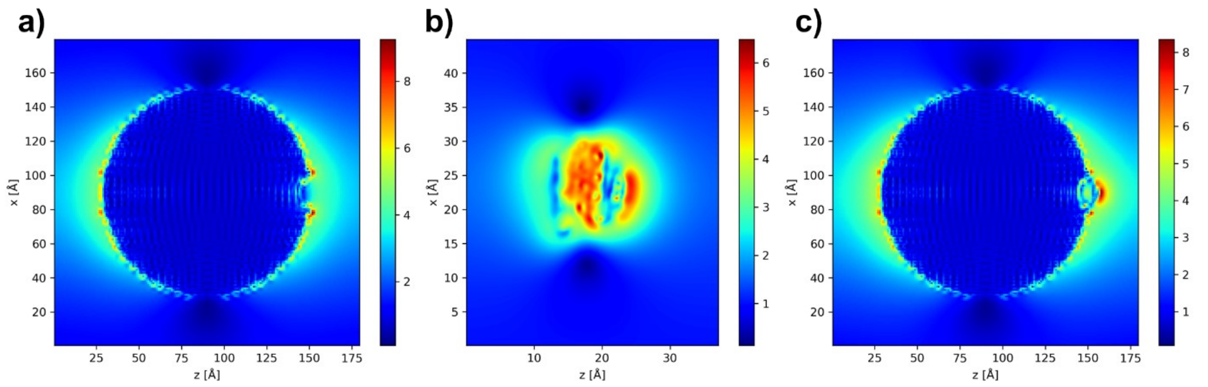

Figure S4. Field enhancement at 532 nm (2.33 eV) is shown for (a) classical subsystem, (b) quantum subsystem (structure 3 considering hydrogen molecule as proton source) and for (c) hybrid quantum-classical system for

Table S2. Charge density differences (CDDs) illustrating the nature of the low-lying bright excitations of structure 1 with an H_2 molecule on Ag surface. Charge transfer takes place from red to blue.

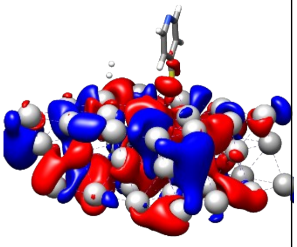<br>State 39<br>Energy: 1.441 eV<br>Osc.: 0.010	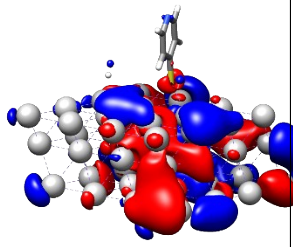<br>State 60<br>Energy: 1.788 eV<br>Osc.: 0.010	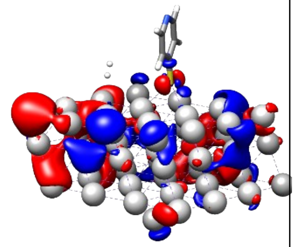<br>State 63<br>Energy: 1.828 eV<br>Osc.: 0.013	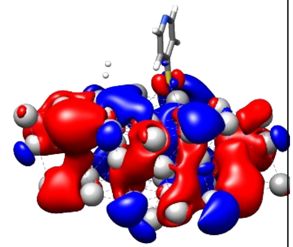<br>State 72<br>Energy: 1.927 eV<br>Osc.: 0.013
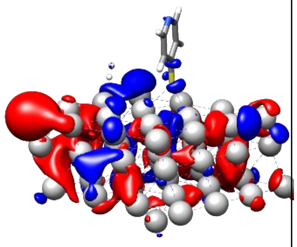<br>State 73<br>Energy: 1.951 eV<br>Osc.: 0.010	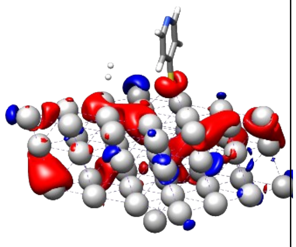<br>State 76<br>Energy: 1.999 eV<br>Osc.: 0.020	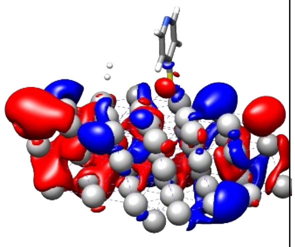<br>State 81<br>Energy: 2.050 eV<br>Osc.: 0.013	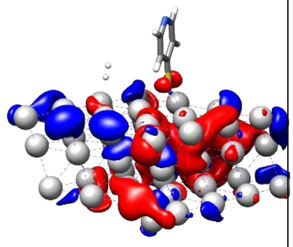<br>State 86<br>Energy: 2.122 eV<br>Osc.: 0.024
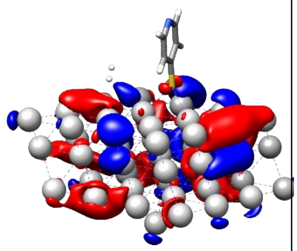<br>State 87<br>Energy: 2.135 eV<br>Osc.: 0.014	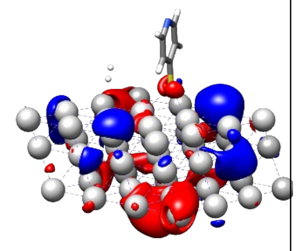<br>State 88<br>Energy: 2.151 eV<br>Osc.: 0.014	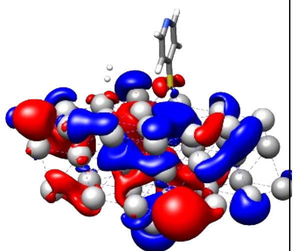<br>State 90<br>Energy: 2.171 eV<br>Osc.: 0.016	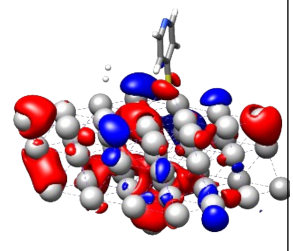<br>State 93<br>Energy: 2.215 eV<br>Osc.: 0.010
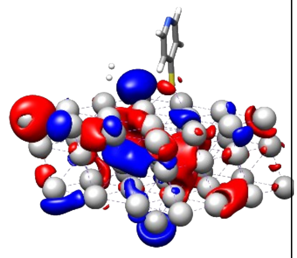<br>State 94<br>Energy: 2.223 eV<br>Osc.: 0.013	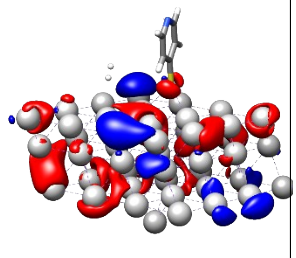<br>State 96<br>Energy: 2.236 eV<br>Osc.: 0.011	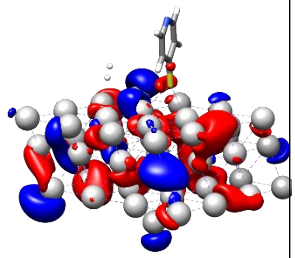<br>State 97<br>Energy: 2.255 eV<br>Osc.: 0.013	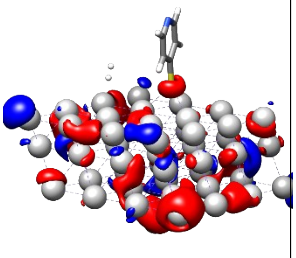<br>State 99<br>Energy: 2.281 eV<br>Osc.: 0.013

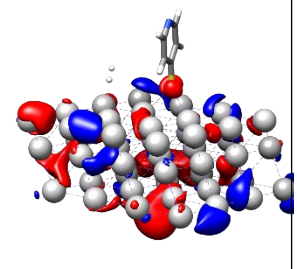

State 100
Energy: 2.289 eV
Osc.: 0.021

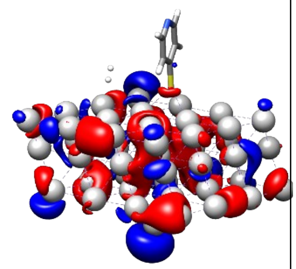

State 106
Energy: 2.353 eV
Osc.: 0.023

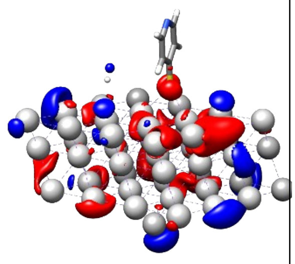

State 107
Energy: 2.360 eV
Osc.: 0.034

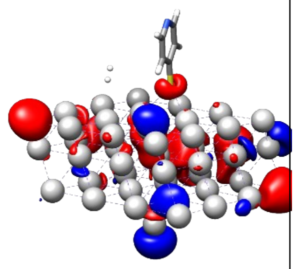

State 112
Energy: 2.434 eV
Osc.: 0.046

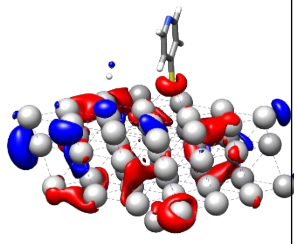

State 113
Energy: 2.442 eV
Osc.: 0.061

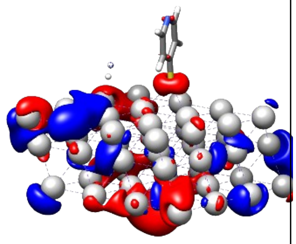

State 115
Energy: 2.461 eV
Osc.: 0.038

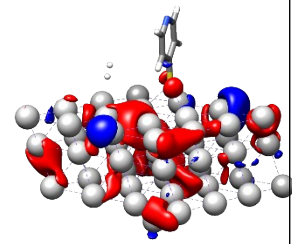

State 116
Energy: 2.481 eV
Osc.: 0.021

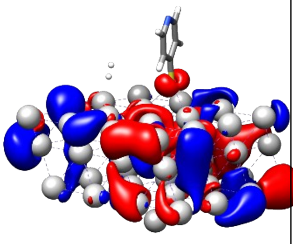

State 117
Energy: 2.484 eV
Osc.: 0.010

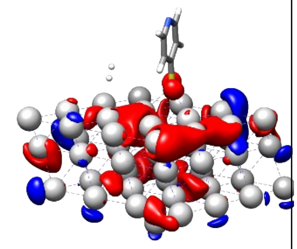

State 118
Energy: 2.501 eV
Osc.: 0.060

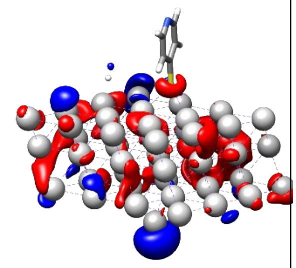

State 119
Energy: 2.508 eV
Osc.: 0.017

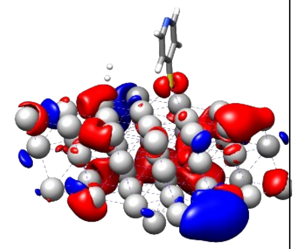

State 120
Energy: 2.510 eV
Osc.: 0.015

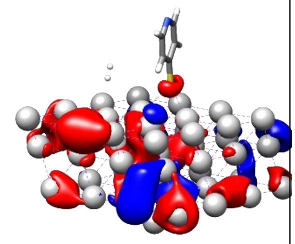

State 121
Energy: 2.524 eV
Osc.: 0.017

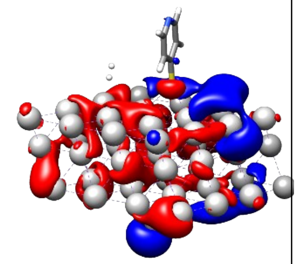

State 123
Energy: 2.549 eV
Osc.: 0.045

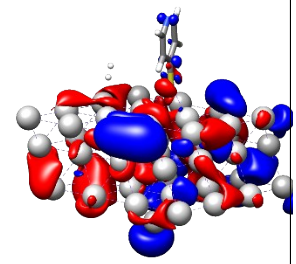

State 124
Energy: 2.558 eV
Osc.: 0.013

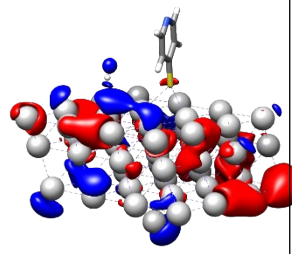

State 126
Energy: 2.569 eV
Osc.: 0.027

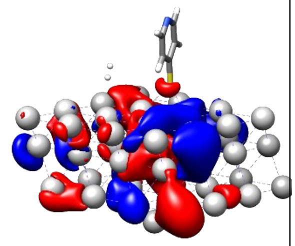

State 127
Energy: 2.576 eV
Osc.: 0.017

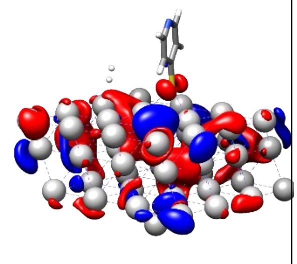
State 128
Energy: 2.587 eV
Osc.: 0.010

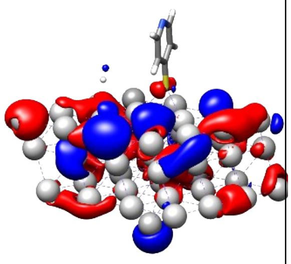
State 129
Energy: 2.594 eV
Osc.: 0.015

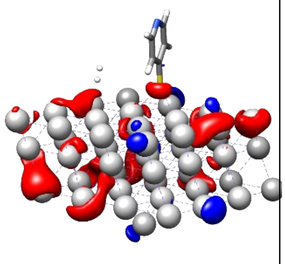
State 130
Energy: 2.608 eV
Osc.: 0.172

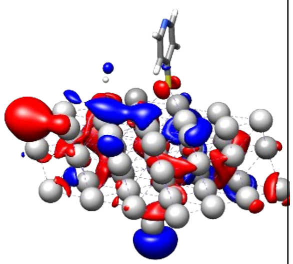
State 131
Energy: 2.614 eV
Osc.: 0.018

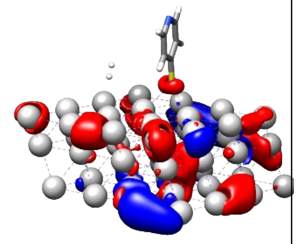
State 132
Energy: 2.637 eV
Osc.: 0.032

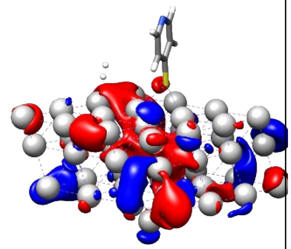
State 133
Energy: 2.639 eV
Osc.: 0.021

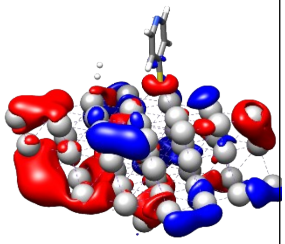
State 134
Energy: 2.656 eV
Osc.: 0.033

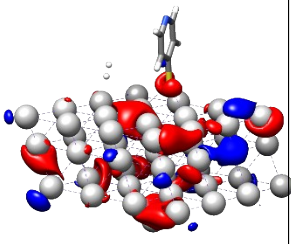
State 135
Energy: 2.661 eV
Osc.: 0.025

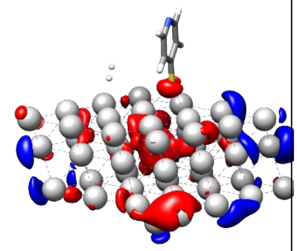
State 136
Energy: 2.664 eV
Osc.: 0.014

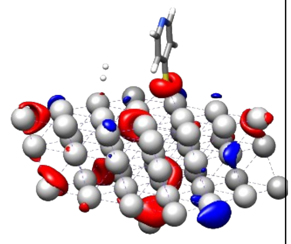
State 138
Energy: 2.676 eV
Osc.: 0.057

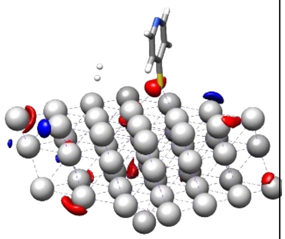
State 139
Energy: 2.686 eV
Osc.: 0.046

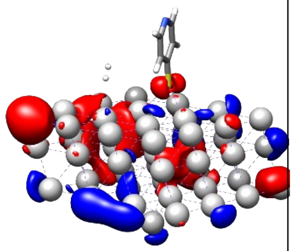
State 140
Energy: 2.698 eV
Osc.: 0.092

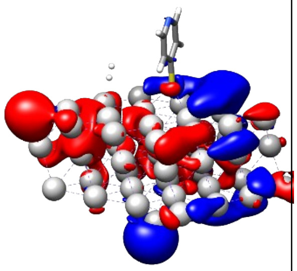
State 142
Energy: 2.709 eV
Osc.: 0.020

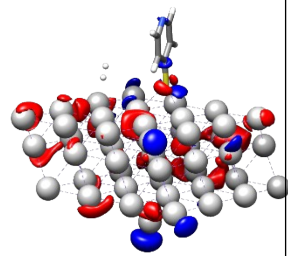
State 143
Energy: 2.728 eV
Osc.: 0.056

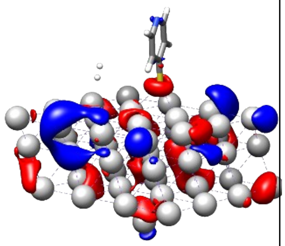
State 144
Energy: 2.729 eV
Osc.: 0.020

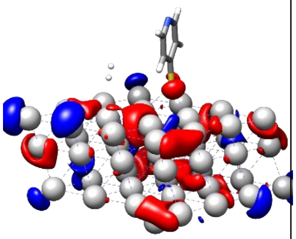
State 145
Energy: 2.744 eV
Osc.: 0.026

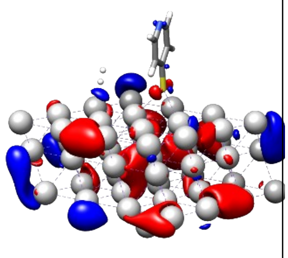

State 146
Energy: 2.750 eV
Osc.: 0.012

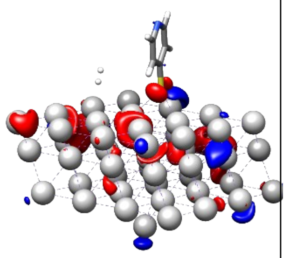

State 147
Energy: 2.763 eV
Osc.: 0.058

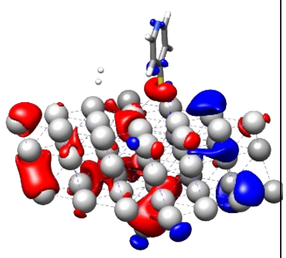

State 148
Energy: 2.772 eV
Osc.: 0.021

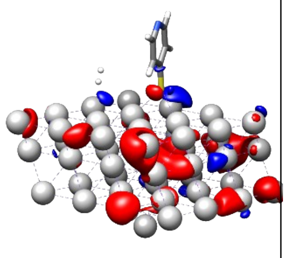

State 149
Energy: 2.777 eV
Osc.: 0.017

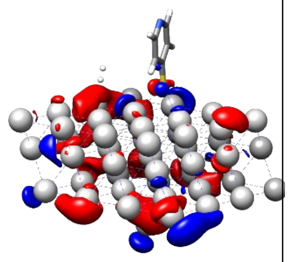

State 150
Energy: 2.785 eV
Osc.: 0.043

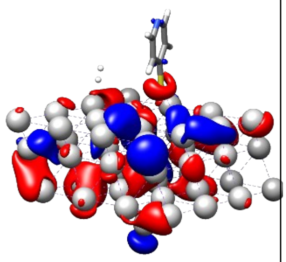

State 153
Energy: 2.813 eV
Osc.: 0.025

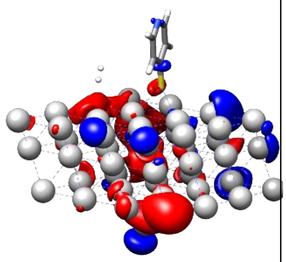

State 154
Energy: 2.817 eV
Osc.: 0.026

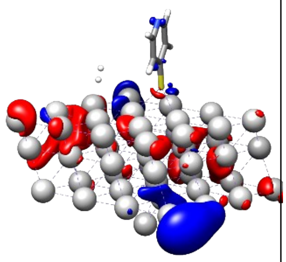

State 155
Energy: 2.821 eV
Osc.: 0.136

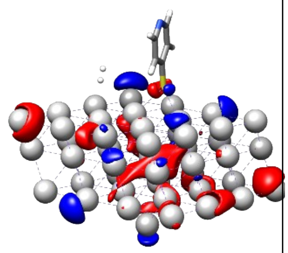

State 156
Energy: 2.826 eV
Osc.: 0.030

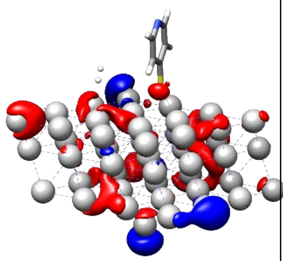

State 157
Energy: 2.829 eV
Osc.: 0.012

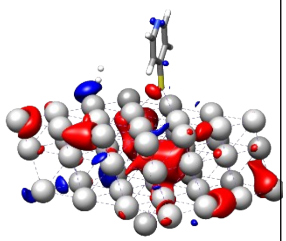

State 159
Energy: 2.862 eV
Osc.: 0.017

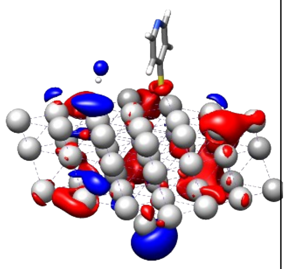

State 161
Energy: 2.868 eV
Osc.: 0.070

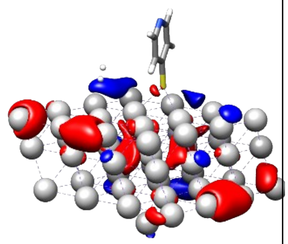

State 163
Energy: 2.893 eV
Osc.: 0.068

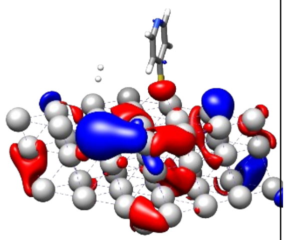

State 164
Energy: 2.896 eV
Osc.: 0.012

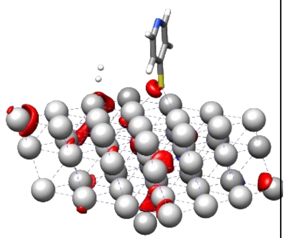

State 165
Energy: 2.912 eV
Osc.: 0.151

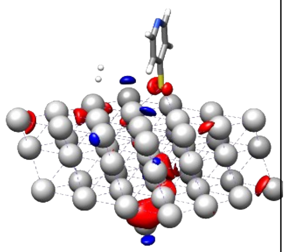

State 166
Energy: 2.922 eV
Osc.: 0.311

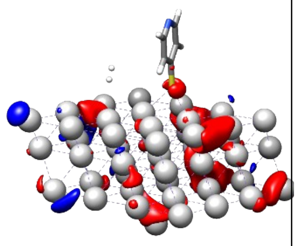<br>State 167<br>Energy: 2.927 eV<br>Osc.: 0.045	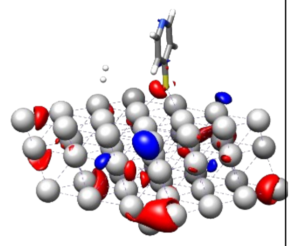<br>State 168<br>Energy: 2.943 eV<br>Osc.: 0.036	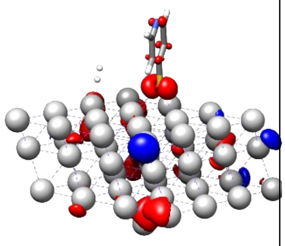<br>State 169<br>Energy: 2.947 eV<br>Osc.: 0.145	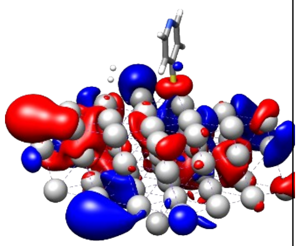<br>State 170<br>Energy: 2.951 eV<br>Osc.: 0.232
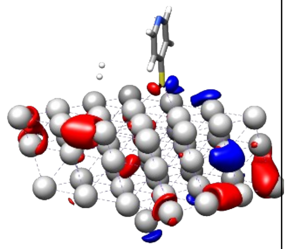<br>State 172<br>Energy: 2.964 eV<br>Osc.: 0.018	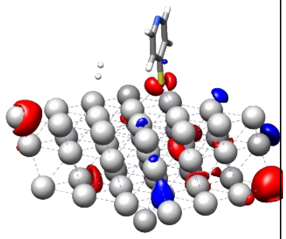<br>State 173<br>Energy: 2.975 eV<br>Osc.: 0.278	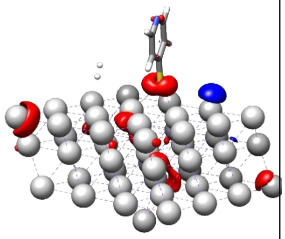<br>State 174<br>Energy: 2.993 eV<br>Osc.: 0.252	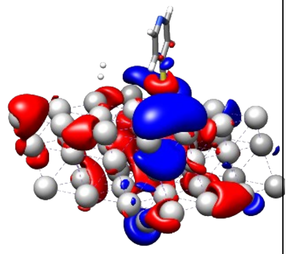<br>State 176<br>Energy: 3.008 eV<br>Osc.: 0.028
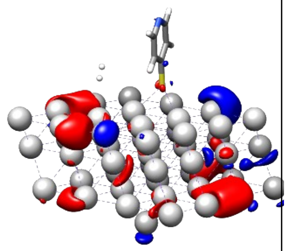<br>State 177<br>Energy: 3.009 eV<br>Osc.: 0.021	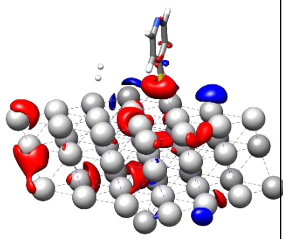<br>State 178<br>Energy: 3.016 eV<br>Osc.: 0.304	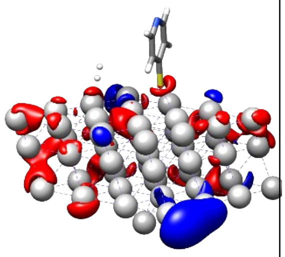<br>State 179<br>Energy: 3.030 eV<br>Osc.: 0.026	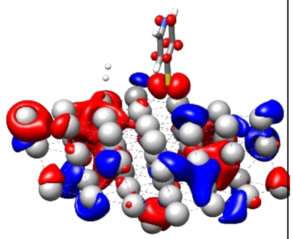<br>State 180<br>Energy: 3.041 eV<br>Osc.: 0.107
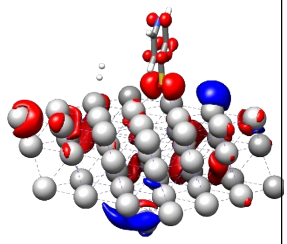<br>State 181<br>Energy: 3.046 eV<br>Osc.: 0.193	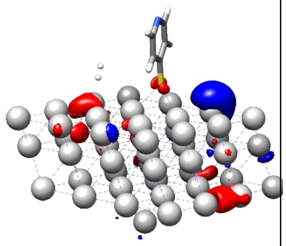<br>State 182<br>Energy: 3.052 eV<br>Osc.: 0.028	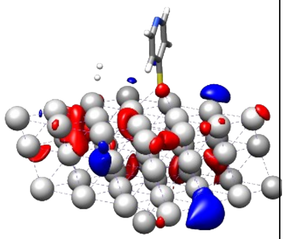<br>State 183<br>Energy: 3.055 eV<br>Osc.: 0.523	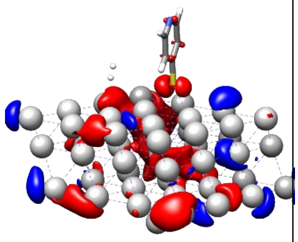<br>State 184<br>Energy: 3.063 eV<br>Osc.: 0.230

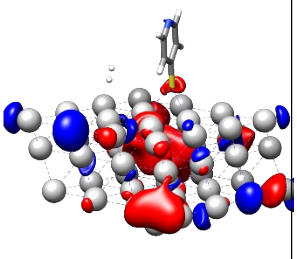<br>State 185<br>Energy: 3.066 eV<br>Osc.: 0.020	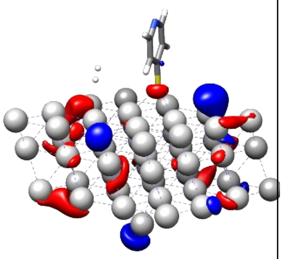<br>State 186<br>Energy: 3.079 eV<br>Osc.: 0.166	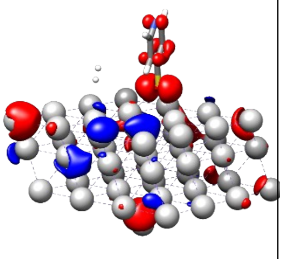<br>State 187<br>Energy: 3.101 eV<br>Osc.: 0.021	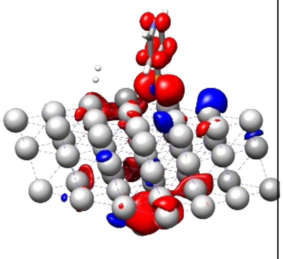<br>State 188<br>Energy: 3.107 eV<br>Osc.: 0.186
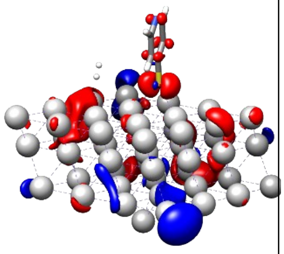<br>State 189<br>Energy: 3.112 eV<br>Osc.: 0.015	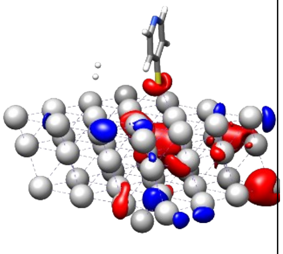<br>State 190<br>Energy: 3.124 eV<br>Osc.: 0.229	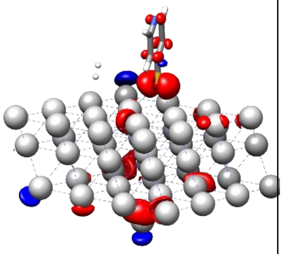<br>State 192<br>Energy: 3.137 eV<br>Osc.: 0.295	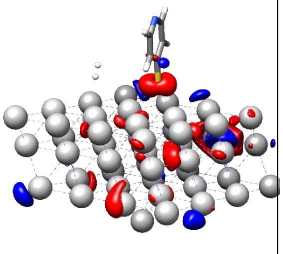<br>State 193<br>Energy: 3.145 eV<br>Osc.: 0.021
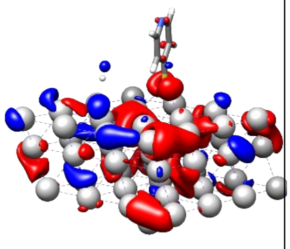<br>State 194<br>Energy: 3.151 eV<br>Osc.: 0.089	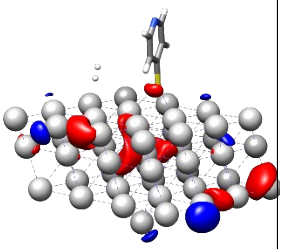<br>State 195<br>Energy: 3.158 eV<br>Osc.: 0.510	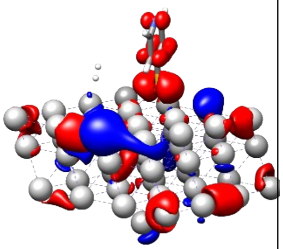<br>State 196<br>Energy: 3.165 eV<br>Osc.: 0.079	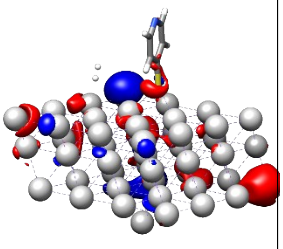<br>State 197<br>Energy: 3.171 eV<br>Osc.: 0.160
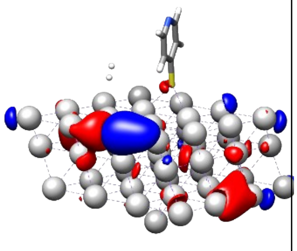<br>State 198<br>Energy: 3.177 eV<br>Osc.: 0.183	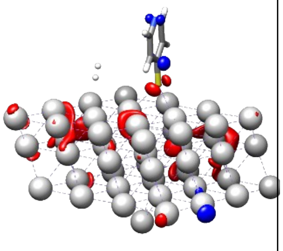<br>State 199<br>Energy: 3.177 eV<br>Osc.: 0.184	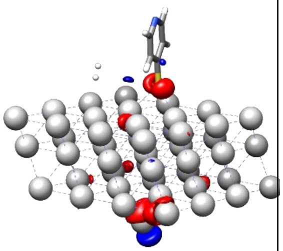<br>State 200<br>Energy: 3.190 eV<br>Osc.: 0.096	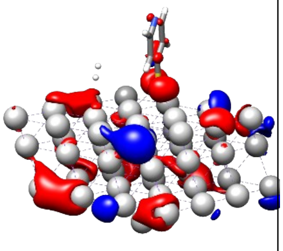<br>State 201<br>Energy: 3.197 eV<br>Osc.: 0.017

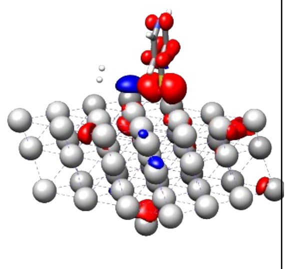<br>State 203<br>Energy: 3.211 eV<br>Osc.: 0.021	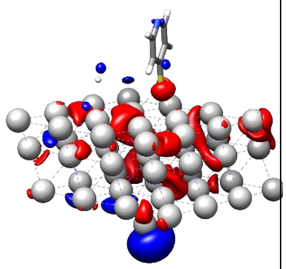<br>State 204<br>Energy: 3.217 eV<br>Osc.: 0.036	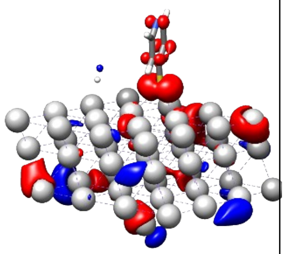<br>State 205<br>Energy: 3.228 eV<br>Osc.: 0.561	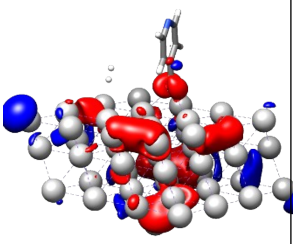<br>State 206<br>Energy: 3.237 eV<br>Osc.: 0.096
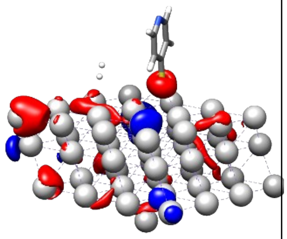<br>State 207<br>Energy: 3.246 eV<br>Osc.: 0.072	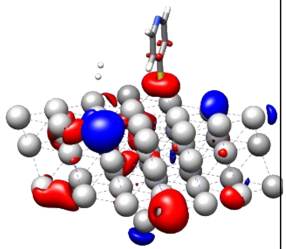<br>State 208<br>Energy: 3.249 eV<br>Osc.: 0.476	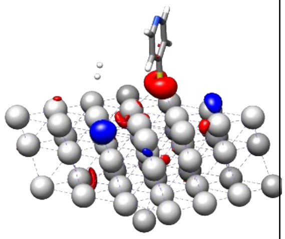<br>State 210<br>Energy: 3.270 eV<br>Osc.: 0.110	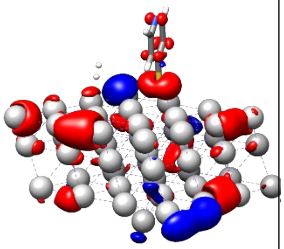<br>State 211<br>Energy: 3.272 eV<br>Osc.: 0.013
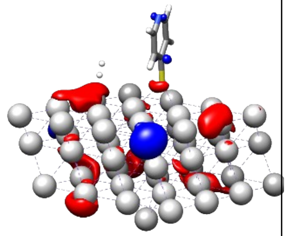<br>State 212<br>Energy: 3.278 eV<br>Osc.: 0.052	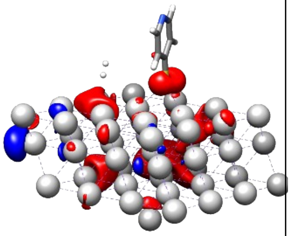<br>State 213<br>Energy: 3.283 eV<br>Osc.: 0.013	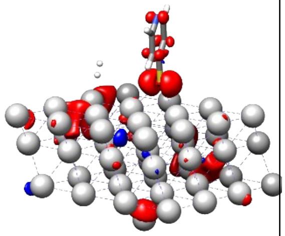<br>State 216<br>Energy: 3.303 eV<br>Osc.: 0.016	

Table S3. Charge density differences (CDDs) illustrating the nature of the low-lying bright excitations of structure 2 with an H_2 molecule on Ag surface. Charge transfer takes place from red to blue.

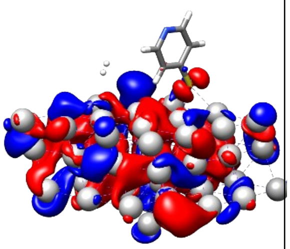<br>State 44<br>Energy: 1.534 eV<br>Osc.: 0.014	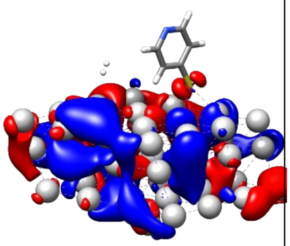<br>State 45<br>Energy: 1.551 eV<br>Osc.: 0.014	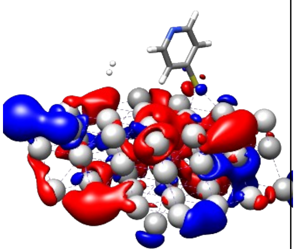<br>State 55<br>Energy: 1.715 eV<br>Osc.: 0.020	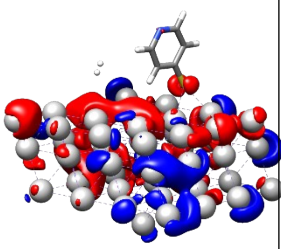<br>State 69<br>Energy: 1.927 eV<br>Osc.: 0.012

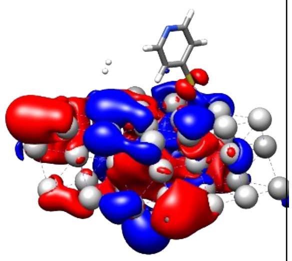

State 75
Energy: 2.000 eV
Osc.: 0.013

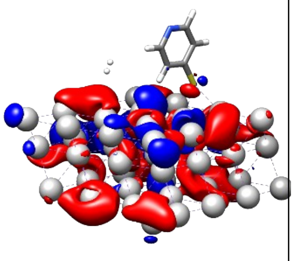

State 79
Energy: 2.049 eV
Osc.: 0.012

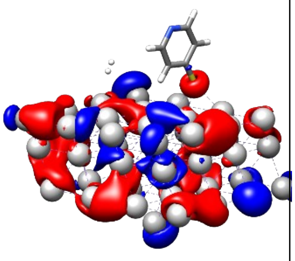

State 82
Energy: 2.093 eV
Osc.: 0.026

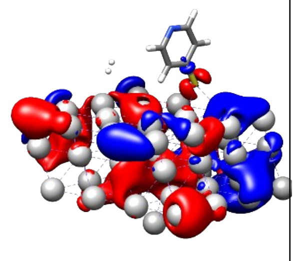

State 85
Energy: 2.131 eV
Osc.: 0.023

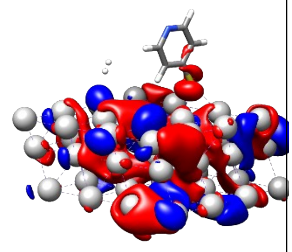

State 88
Energy: 2.169 eV
Osc.: 0.020

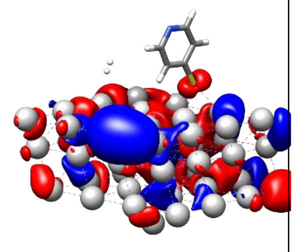

State 89
Energy: 2.181 eV
Osc.: 0.012

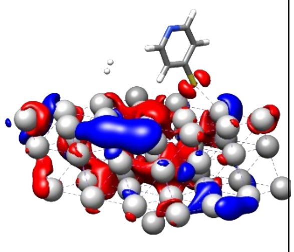

State 93
Energy: 2.227 eV
Osc.: 0.014

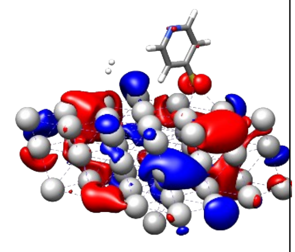

State 94
Energy: 2.236 eV
Osc.: 0.008

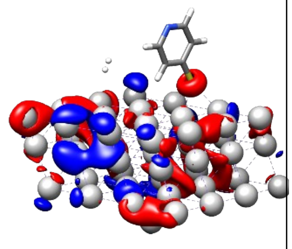

State 95
Energy: 2.246 eV
Osc.: 0.007

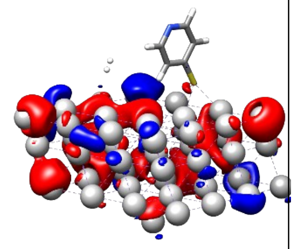

State 96
Energy: 2.253 eV
Osc.: 0.009

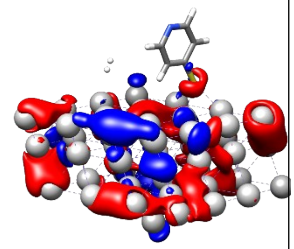

State 97
Energy: 2.286 eV
Osc.: 0.012

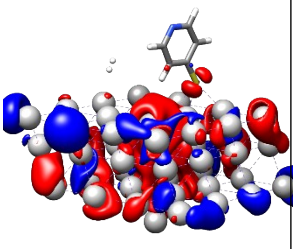

State 98
Energy: 2.297 eV
Osc.: 0.008

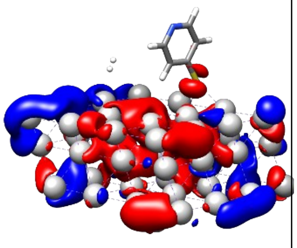

State 99
Energy: 2.302 eV
Osc.: 0.001

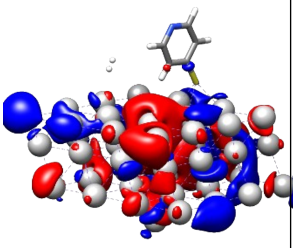

State 100
Energy: 2.313 eV
Osc.: 0.002

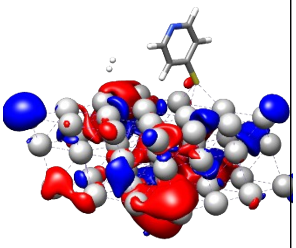

State 101
Energy: 2.318 eV
Osc.: 0.003

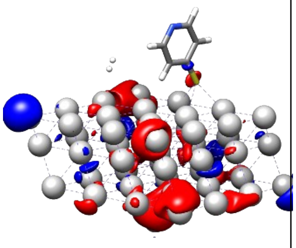

State 102
Energy: 2.338 eV
Osc.: 0.013

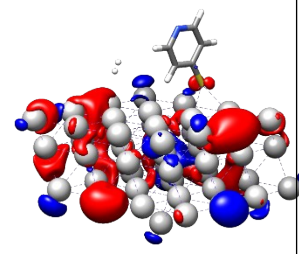

State 103
Energy: 2.356 eV
Osc.: 0.000

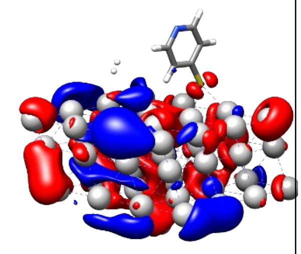

State 104
Energy: 2.361 eV
Osc.: 0.041

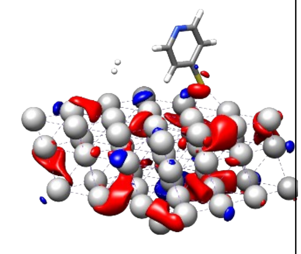

State 105
Energy: 2.377 eV
Osc.: 0.008

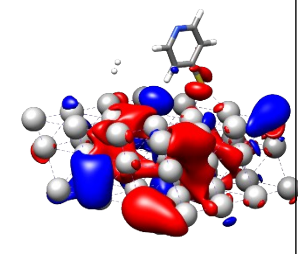

State 106
Energy: 2.381 eV
Osc.: 0.000

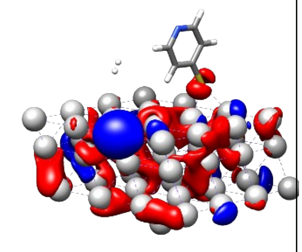

State 107
Energy: 2.393 eV
Osc.: 0.019

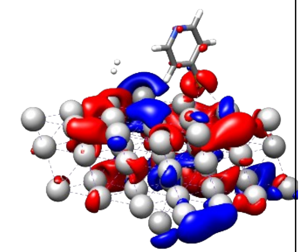

State 108
Energy: 2.399 eV
Osc.: 0.002

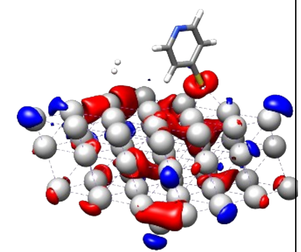

State 109
Energy: 2.419 eV
Osc.: 0.014

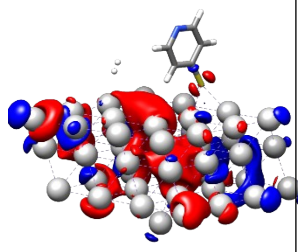

State 110
Energy: 2.432 eV
Osc.: 0.002

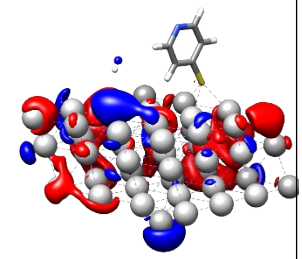

State 111
Energy: 2.434 eV
Osc.: 0.003

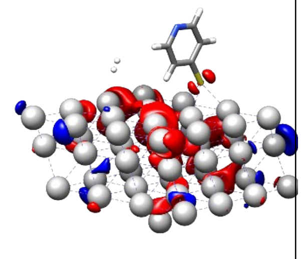

State 113
Energy: 2.459 eV
Osc.: 0.029

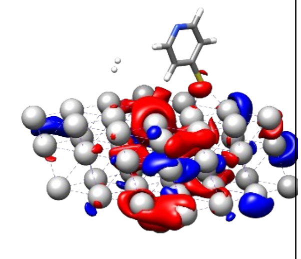

State 114
Energy: 2.470 eV
Osc.: 0.032

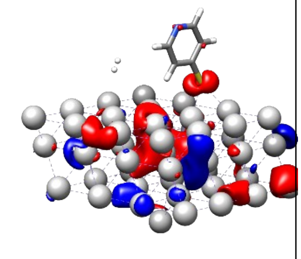

State 117
Energy: 2.507 eV
Osc.: 0.027

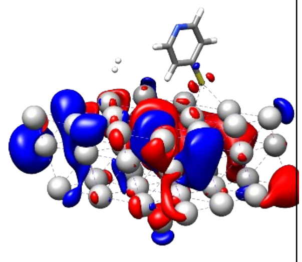

State 121
Energy: 2.542 eV
Osc.: 0.030

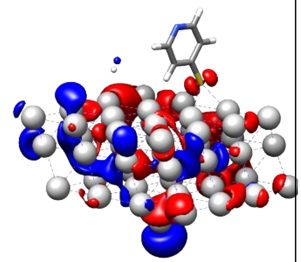

State 122
Energy: 2.543 eV
Osc.: 0.013

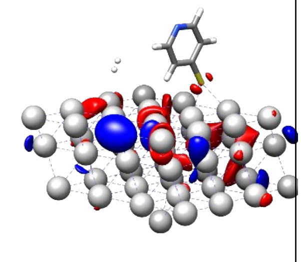

State 123
Energy: 2.558 eV
Osc.: 0.050

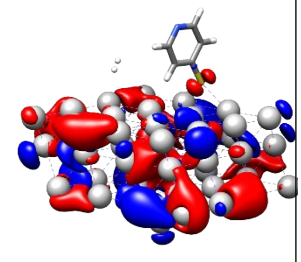

State 126
Energy: 2.581 eV
Osc.: 0.010

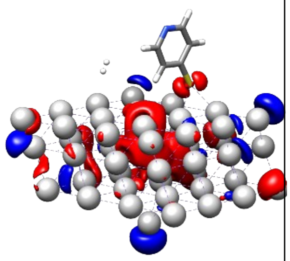

State 127
Energy: 2.590 eV
Osc.: 0.033

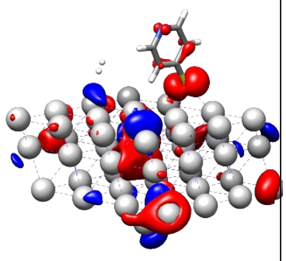

State 130
Energy: 2.624 eV
Osc.: 0.043

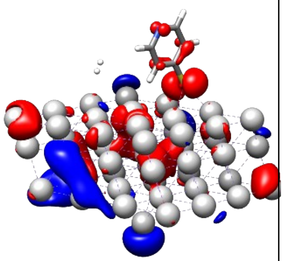

State 131
Energy: 2.641 eV
Osc.: 0.084

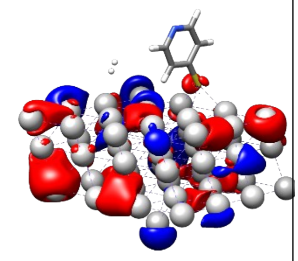

State 132
Energy: 2.650 eV
Osc.: 0.019

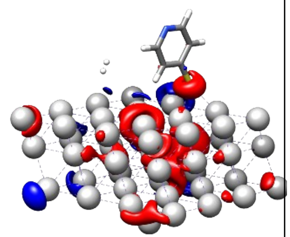

State 135
Energy: 2.677 eV
Osc.: 0.014

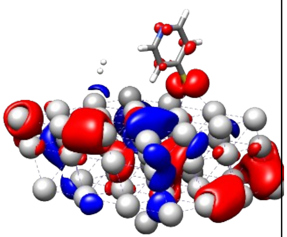

State 137
Energy: 2.693 eV
Osc.: 0.024

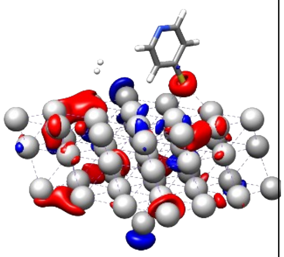

State 140
Energy: 2.722 eV
Osc.: 0.112

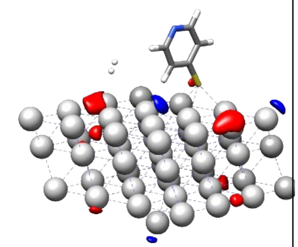

State 141
Energy: 2.726 eV
Osc.: 0.276

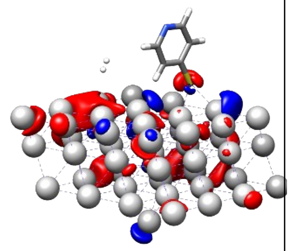

State 142
Energy: 2.736 eV
Osc.: 0.074

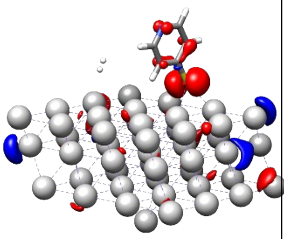

State 143
Energy: 2.746 eV
Osc.: 0.010

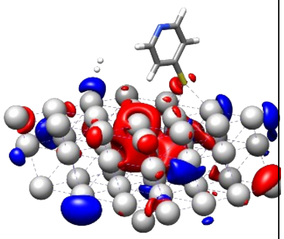

State 144
Energy: 2.748 eV
Osc.: 0.118

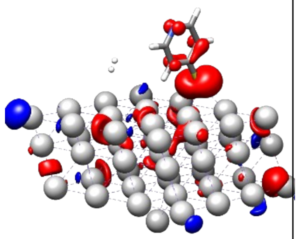

State 147
Energy: 2.782 eV
Osc.: 0.103

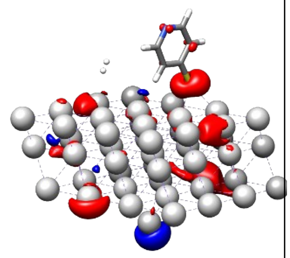

State 149
Energy: 2.797 eV
Osc.: 0.023

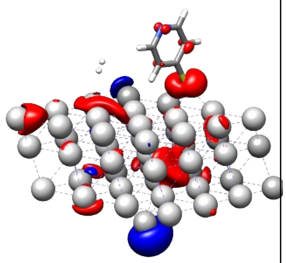

State 150
Energy: 2.801 eV
Osc.: 0.131

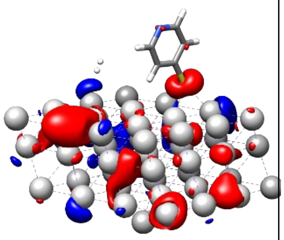

State 151
Energy: 2.803 eV
Osc.: 0.053

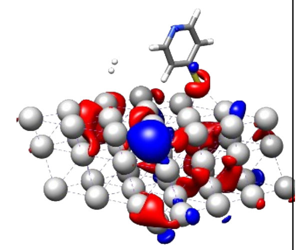

State 152
Energy: 2.817 eV
Osc.: 0.015

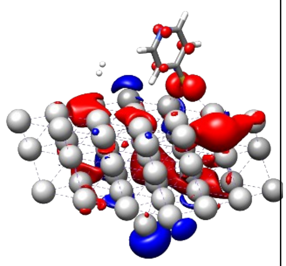
State 153
Energy: 2.818 eV
Osc.: 0.022

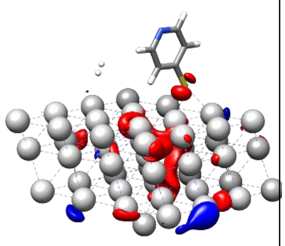
State 155
Energy: 2.849 eV
Osc.: 0.031

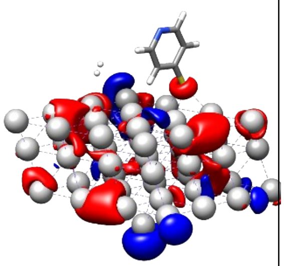
State 157
Energy: 2.862 eV
Osc.: 0.066

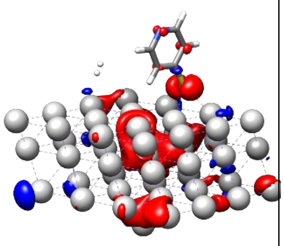
State 158
Energy: 2.871 eV
Osc.: 0.086

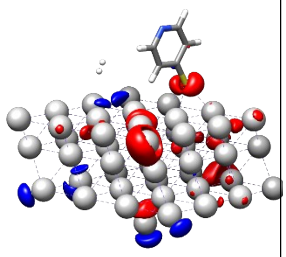
State 159
Energy: 2.873 eV
Osc.: 0.517

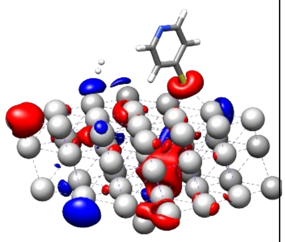
State 160
Energy: 2.880 eV
Osc.: 0.046

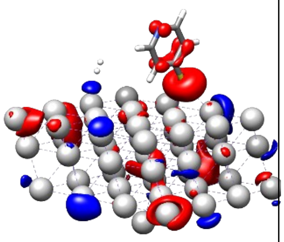
State 161
Energy: 2.895 eV
Osc.: 0.029

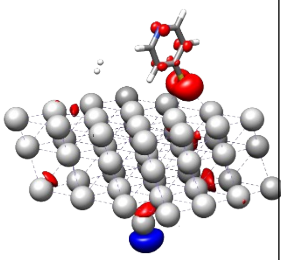
State 162
Energy: 2.901 eV
Osc.: 0.079

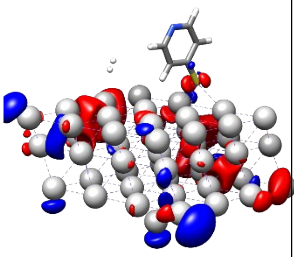
State 163
Energy: 2.909 eV
Osc.: 0.082

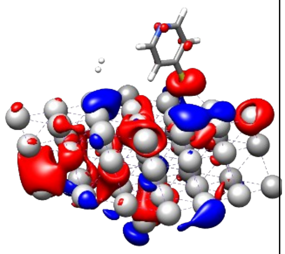
State 166
Energy: 2.935 eV
Osc.: 0.025

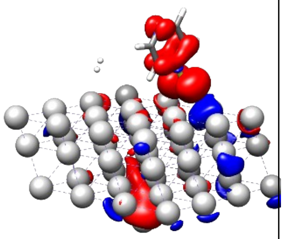
State 167
Energy: 2.945 eV
Osc.: 0.018

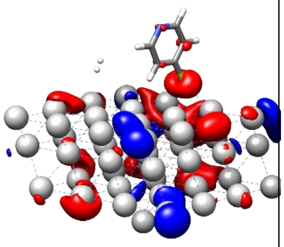
State 168
Energy: 2.950 eV
Osc.: 0.013

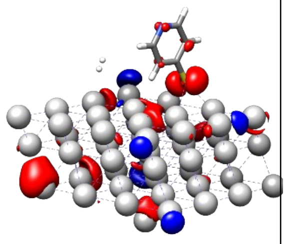
State 169
Energy: 2.956 eV
Osc.: 0.070

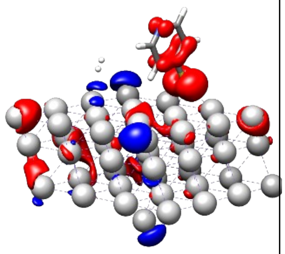
State 170
Energy: 2.977 eV
Osc.: 0.013

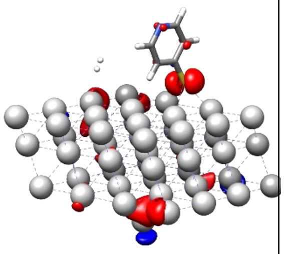
State 171
Energy: 2.986 eV
Osc.: 0.033

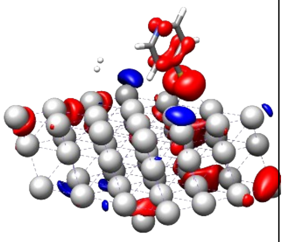
State 172
Energy: 2.996 eV
Osc.: 0.248

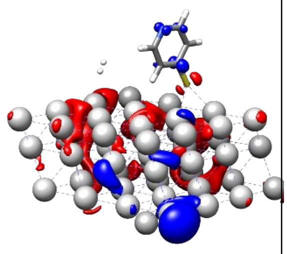

State 173
Energy: 3.000 eV
Osc.: 0.216

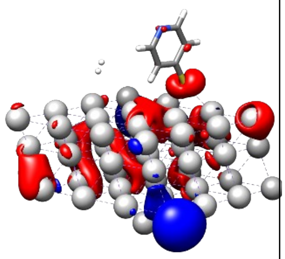

State 174
Energy: 3.004 eV
Osc.: 0.231

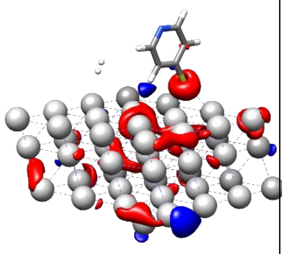

State 175
Energy: 3.017 eV
Osc.: 0.107

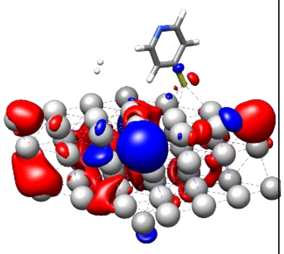

State 176
Energy: 3.024 eV
Osc.: 0.013

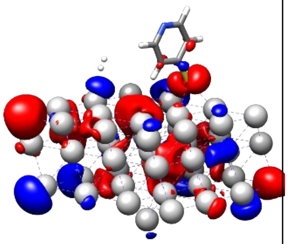

State 177
Energy: 3.035 eV
Osc.: 0.180

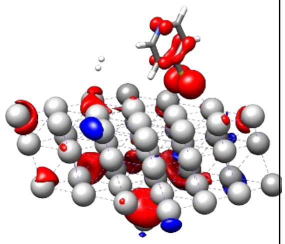

State 178
Energy: 3.041 eV
Osc.: 0.078

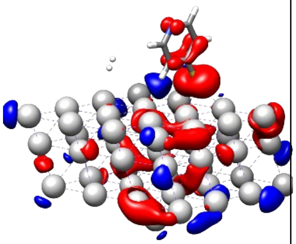

State 179
Energy: 3.050 eV
Osc.: 0.010

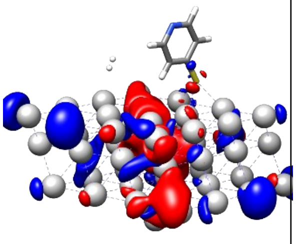

State 180
Energy: 3.057 eV
Osc.: 0.028

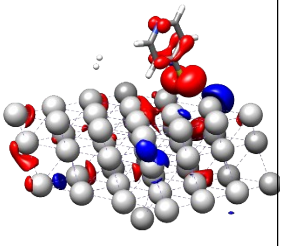

State 181
Energy: 3.067 eV
Osc.: 0.136

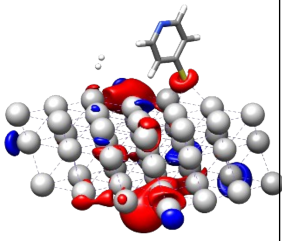

State 182
Energy: 3.069 eV
Osc.: 0.100

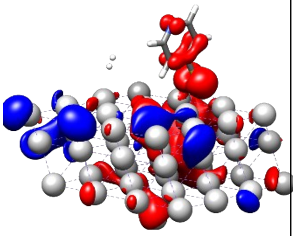

State 183
Energy: 3.082 eV
Osc.: 0.014

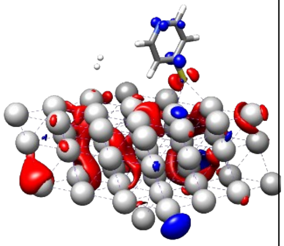

State 184
Energy: 3.089 eV
Osc.: 0.012

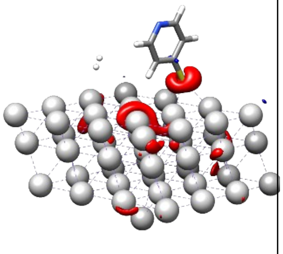

State 185
Energy: 3.091 eV
Osc.: 0.423

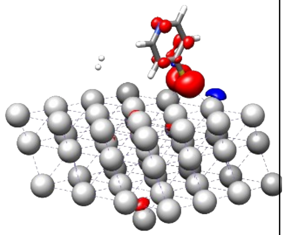

State 186
Energy: 3.095 eV
Osc.: 0.488

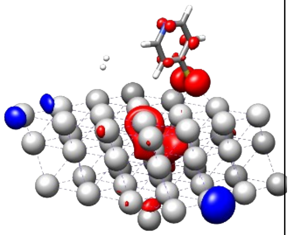

State 187
Energy: 3.106 eV
Osc.: 0.793

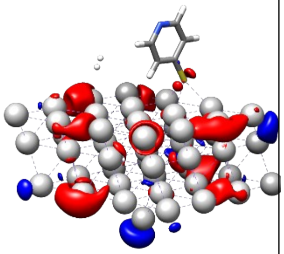

State 188
Energy: 3.112 eV
Osc.: 0.091

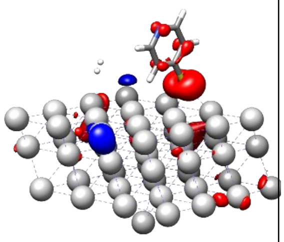

State 189
Energy: 3.120 eV
Osc.: 0.029

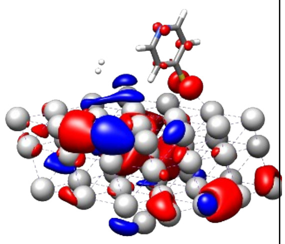

State 191
Energy: 3.136 eV
Osc.: 0.395

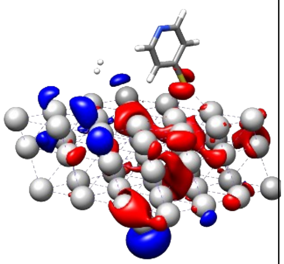

State 192
Energy: 3.139 eV
Osc.: 0.640

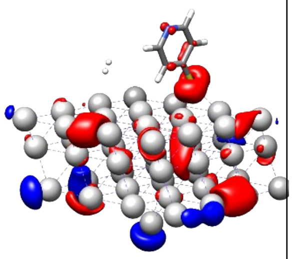

State 193
Energy: 3.146 eV
Osc.: 0.010

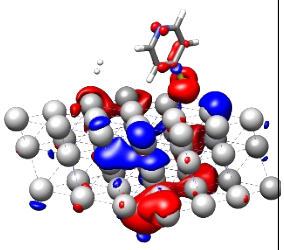

State 195
Energy: 3.163 eV
Osc.: 0.195

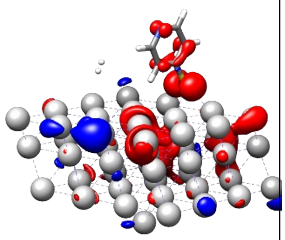

State 196
Energy: 3.173 eV
Osc.: 0.141

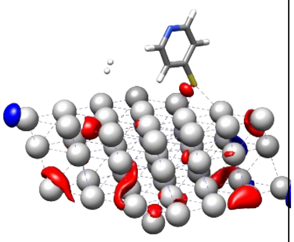

State 198
Energy: 3.189 eV
Osc.: 0.161

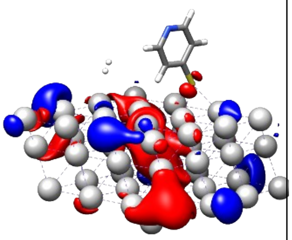

State 200
Energy: 3.202 eV
Osc.: 0.010

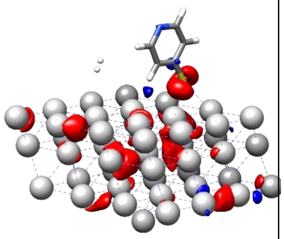

State 201
Energy: 3.206 eV
Osc.: 0.068

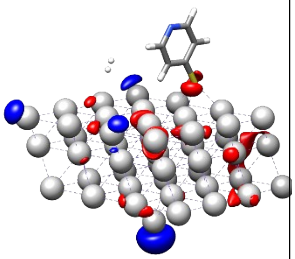

State 202
Energy: 3.213 eV
Osc.: 0.062

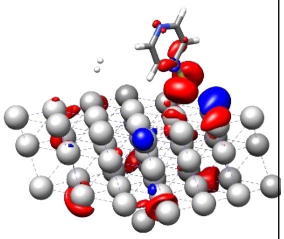

State 204
Energy: 3.228 eV
Osc.: 0.021

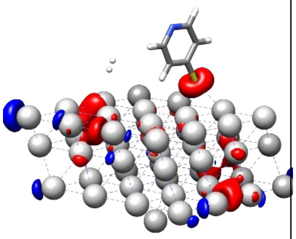

State 205
Energy: 3.229 eV
Osc.: 0.034

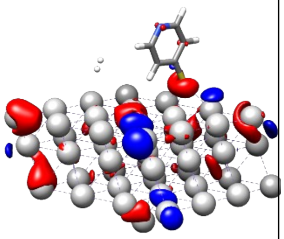

State 206
Energy: 3.233 eV
Osc.: 0.027

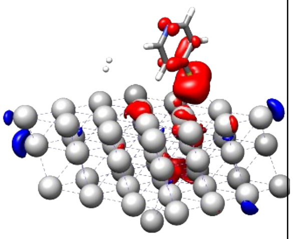

State 207
Energy: 3.241 eV
Osc.: 0.051

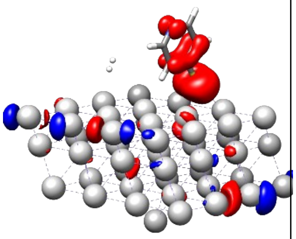

State 208
Energy: 3.248 eV
Osc.: 0.016

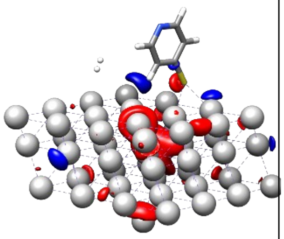

State 209
Energy: 3.252 eV
Osc.: 0.032

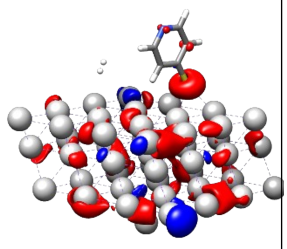<br>State 210<br>Energy: 3.264 eV<br>Osc.: 0.279	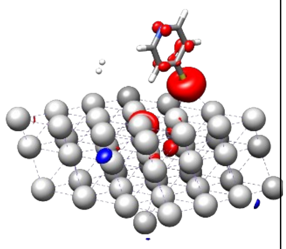<br>State 211<br>Energy: 3.265 eV<br>Osc.: 0.028	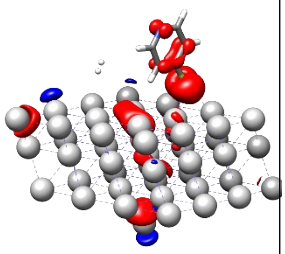<br>State 212<br>Energy: 3.274 eV<br>Osc.: 0.014	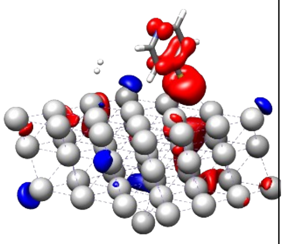<br>State 213<br>Energy: 3.287 eV<br>Osc.: 0.026
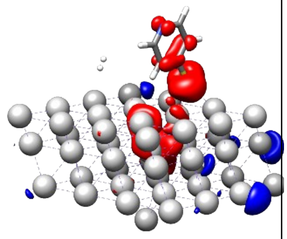<br>State 214<br>Energy: 3.296 eV<br>Osc.: 0.262	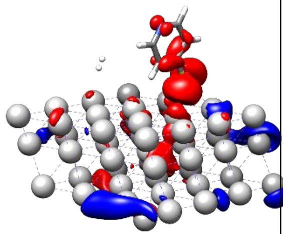<br>State 215<br>Energy: 3.302 eV<br>Osc.: 0.252	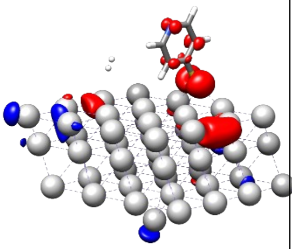<br>State 216<br>Energy: 3.304 eV<br>Osc.: 0.036	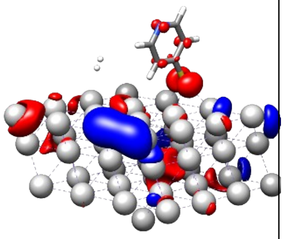<br>State 217<br>Energy: 3.314 eV<br>Osc.: 0.045
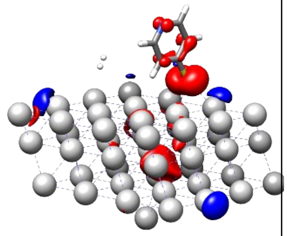<br>State 218<br>Energy: 3.320 eV<br>Osc.: 0.047	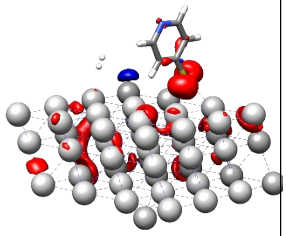<br>State 219<br>Energy: 3.326 eV<br>Osc.: 0.060	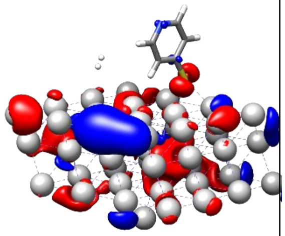<br>State 220<br>Energy: 3.337 eV<br>Osc.: 0.059	

Table S4. Charge density differences (CDDs) illustrating the nature of the low-lying bright excitations of structure 3 with an H_2 molecule on Ag surface. Charge transfer takes place from red to blue.

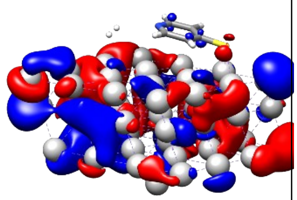<br>State 44<br>Energy: 1.549 eV<br>Osc.: 0.025	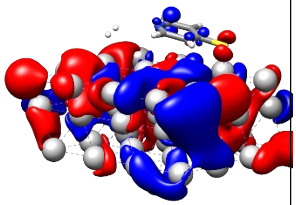<br>State 47<br>Energy: 1.605 eV<br>Osc.: 0.026	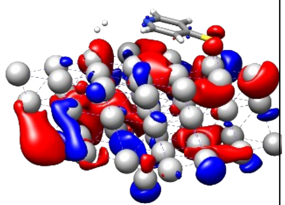<br>State 55<br>Energy: 1.749 eV<br>Osc.: 0.014	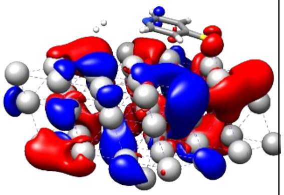<br>State 60<br>Energy: 1.792 eV<br>Osc.: 0.015

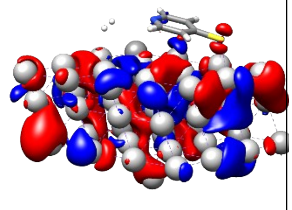<br>State 61<br>Energy: 1.803 eV<br>Osc.: 0.015	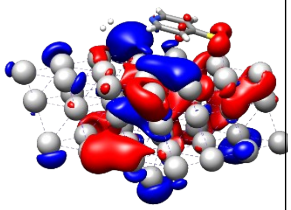<br>State 72<br>Energy: 1.957 eV<br>Osc.: 0.015	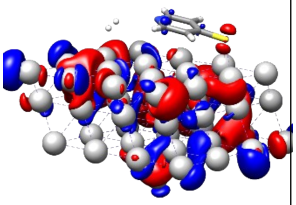<br>State 83<br>Energy: 2.110 eV<br>Osc.: 0.019	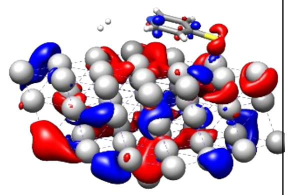<br>State 88<br>Energy: 2.189 eV<br>Osc.: 0.010
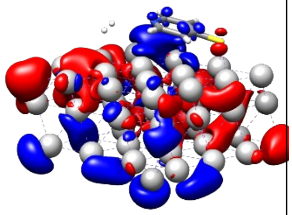<br>State 89<br>Energy: 2.199 eV<br>Osc.: 0.010	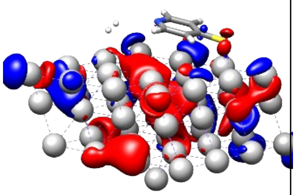<br>State 92<br>Energy: 2.223 eV<br>Osc.: 0.014	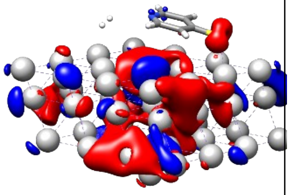<br>State 93<br>Energy: 2.243 eV<br>Osc.: 0.022	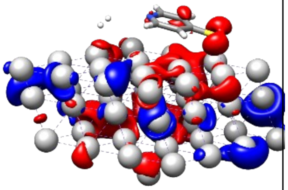<br>State 94<br>Energy: 2.255 eV<br>Osc.: 0.005
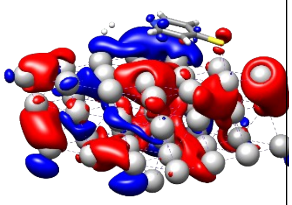<br>State 95<br>Energy: 2.258 eV<br>Osc.: 0.003	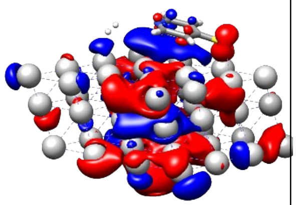<br>State 96<br>Energy: 2.271 eV<br>Osc.: 0.003	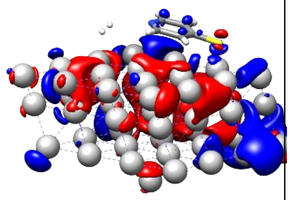<br>State 97<br>Energy: 2.278 eV<br>Osc.: 0.000	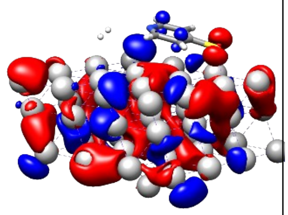<br>State 98<br>Energy: 2.281 eV<br>Osc.: 0.007
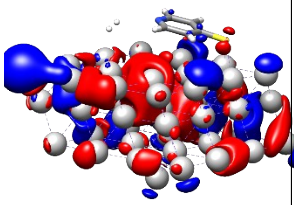<br>State 99<br>Energy: 2.302 eV<br>Osc.: 0.003	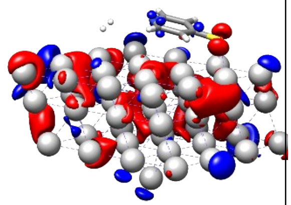<br>State 100<br>Energy: 2.307 eV<br>Osc.: 0.014	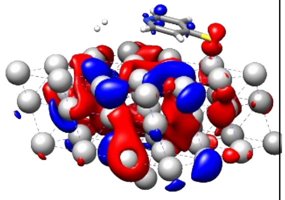<br>State 101<br>Energy: 2.314 eV<br>Osc.: 0.003	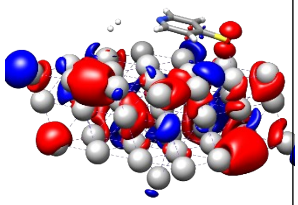<br>State 102<br>Energy: 2.328 eV<br>Osc.: 0.001

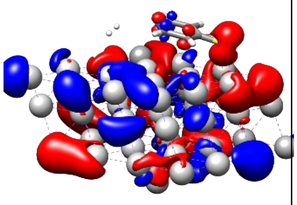<br>State 103<br>Energy: 2.334 eV<br>Osc.: 0.004	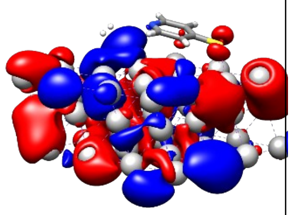<br>State 104<br>Energy: 2.354 eV<br>Osc.: 0.002	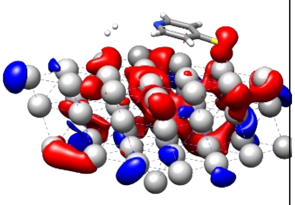<br>State 105<br>Energy: 2.359 eV<br>Osc.: 0.041	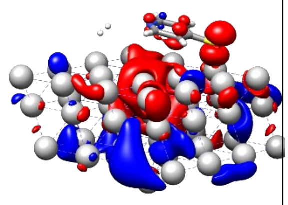<br>State 106<br>Energy: 2.381 eV<br>Osc.: 0.071
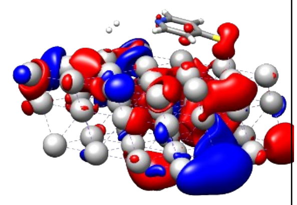<br>State 107<br>Energy: 2.401 eV<br>Osc.: 0.025	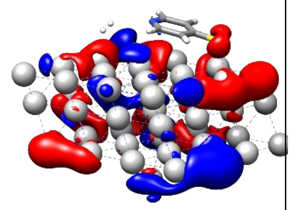<br>State 108<br>Energy: 2.413 eV<br>Osc.: 0.018	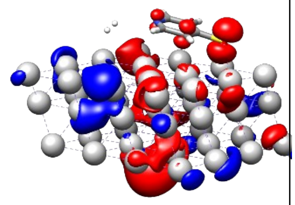<br>State 109<br>Energy: 2.421 eV<br>Osc.: 0.015	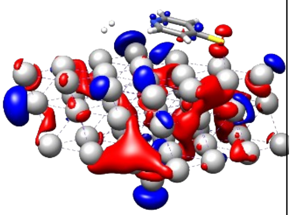<br>State 110<br>Energy: 2.428 eV<br>Osc.: 0.013
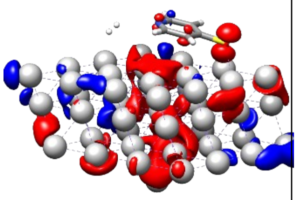<br>State 111<br>Energy: 2.434 eV<br>Osc.: 0.013	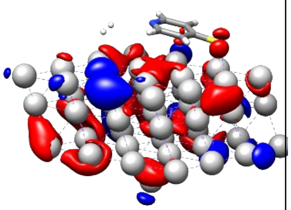<br>State 112<br>Energy: 2.441 eV<br>Osc.: 0.021	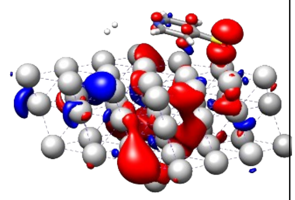<br>State 115<br>Energy: 2.469 eV<br>Osc.: 0.038	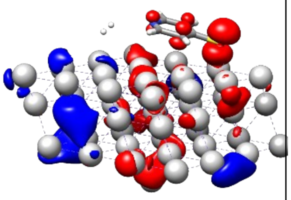<br>State 116<br>Energy: 2.479 eV<br>Osc.: 0.034
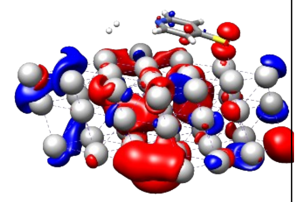<br>State 120<br>Energy: 2.520 eV<br>Osc.: 0.011	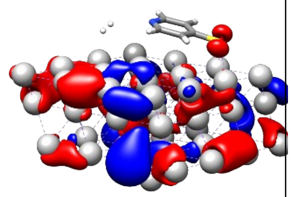<br>State 121<br>Energy: 2.533 eV<br>Osc.: 0.017	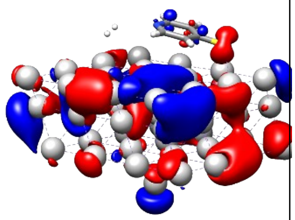<br>State 122<br>Energy: 2.539 eV<br>Osc.: 0.013	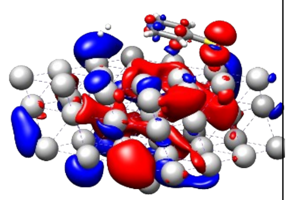<br>State 123<br>Energy: 2.557 eV<br>Osc.: 0.068

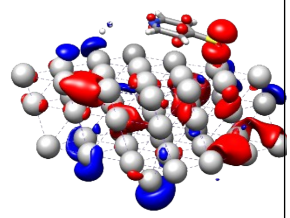
State 124
Energy: 2.571 eV
Osc.: 0.031

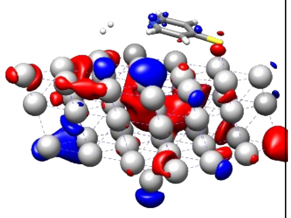
State 125
Energy: 2.575 eV
Osc.: 0.023

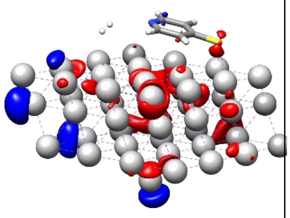
State 129
Energy: 2.607 eV
Osc.: 0.056

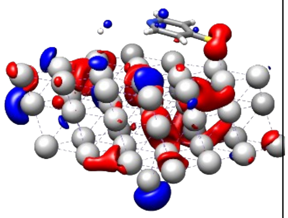
State 131
Energy: 2.628 eV
Osc.: 0.016

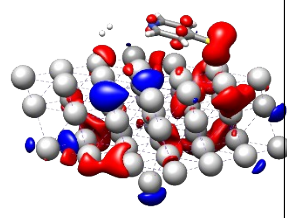
State 132
Energy: 2.634 eV
Osc.: 0.040

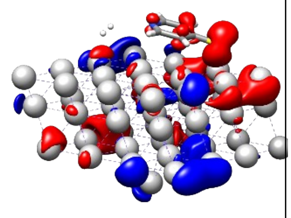
State 135
Energy: 2.651 eV
Osc.: 0.033

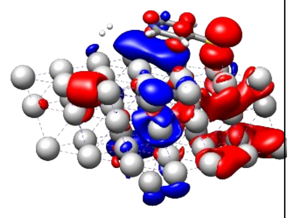
State 136
Energy: 2.659 eV
Osc.: 0.042

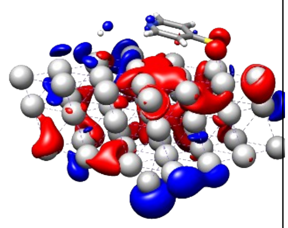
State 139
Energy: 2.688 eV
Osc.: 0.030

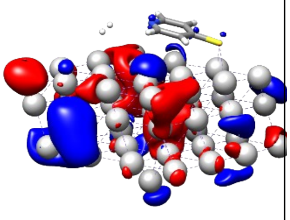
State 141
Energy: 2.722 eV
Osc.: 0.030

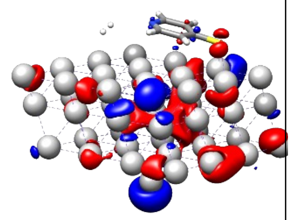
State 142
Energy: 2.733 eV
Osc.: 0.027

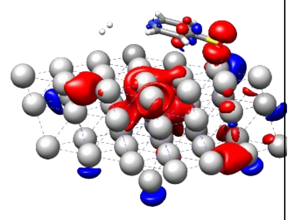
State 143
Energy: 2.739 eV
Osc.: 0.061

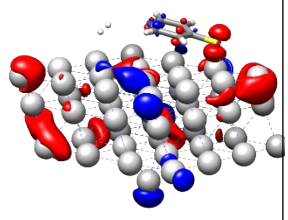
State 145
Energy: 2.749 eV
Osc.: 0.022

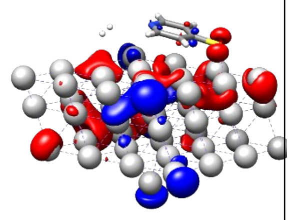
State 146
Energy: 2.762 eV
Osc.: 0.050

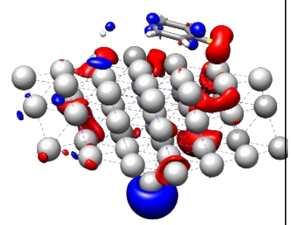
State 148
Energy: 2.777 eV
Osc.: 0.157

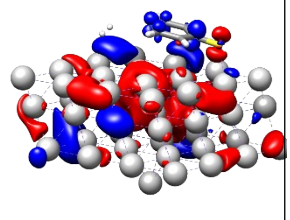
State 149
Energy: 2.782 eV
Osc.: 0.269

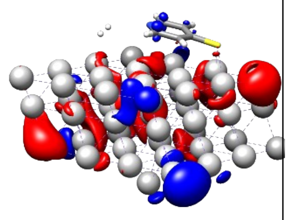
State 150
Energy: 2.793 eV
Osc.: 0.020

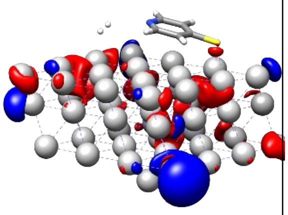<br>State 151<br>Energy: 2.800 eV<br>Osc.: 0.050	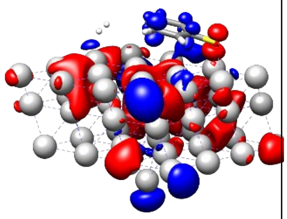<br>State 152<br>Energy: 2.802 eV<br>Osc.: 0.011	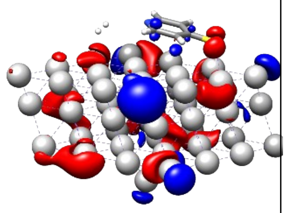<br>State 154<br>Energy: 2.824 eV<br>Osc.: 0.075	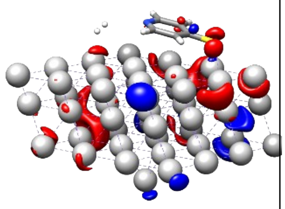<br>State 155<br>Energy: 2.831 eV<br>Osc.: 0.043
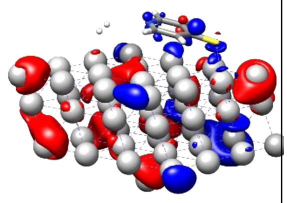<br>State 156<br>Energy: 2.842 eV<br>Osc.: 0.072	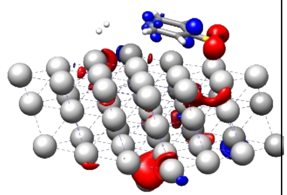<br>State 157<br>Energy: 2.844 eV<br>Osc.: 0.160	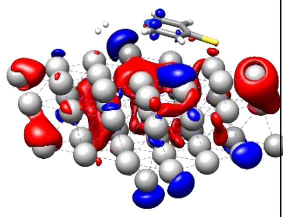<br>State 158<br>Energy: 2.857 eV<br>Osc.: 0.048	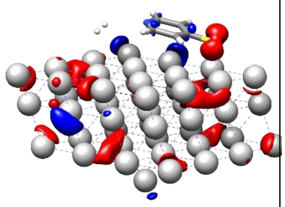<br>State 159<br>Energy: 2.875 eV<br>Osc.: 0.021
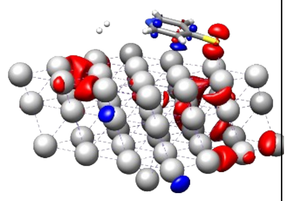<br>State 161<br>Energy: 2.890 eV<br>Osc.: 0.021	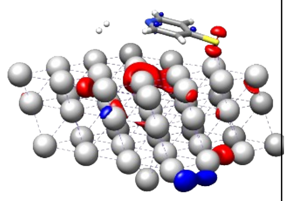<br>State 162<br>Energy: 2.894 eV<br>Osc.: 0.139	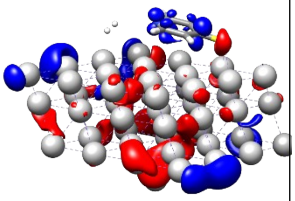<br>State 163<br>Energy: 2.905 eV<br>Osc.: 0.061	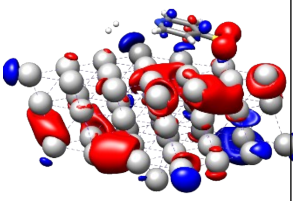<br>State 164<br>Energy: 2.912 eV<br>Osc.: 0.011
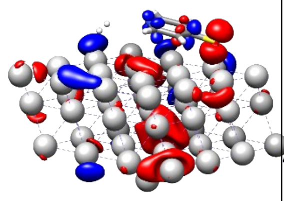<br>State 165<br>Energy: 2.915 eV<br>Osc.: 0.020	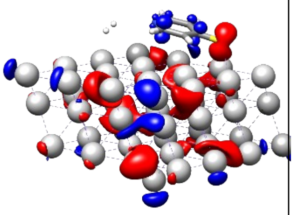<br>State 166<br>Energy: 2.919 eV<br>Osc.: 0.051	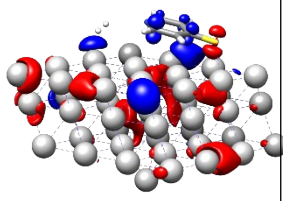<br>State 167<br>Energy: 2.933 eV<br>Osc.: 0.013	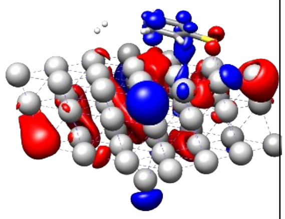<br>State 168<br>Energy: 2.946 eV<br>Osc.: 0.036

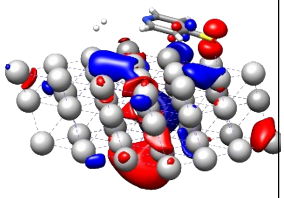

State 169
Energy: 2.949 eV
Osc.: 0.126

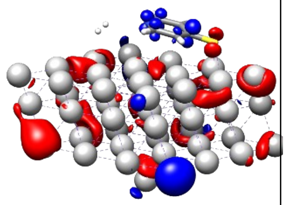

State 170
Energy: 2.954 eV
Osc.: 0.017

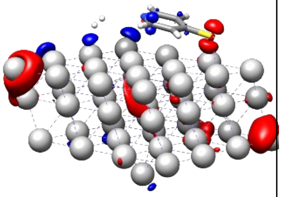

State 171
Energy: 2.961 eV
Osc.: 0.269

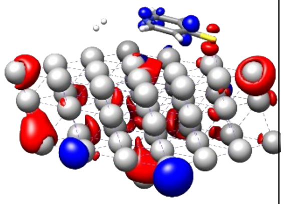

State 172
Energy: 2.973 eV
Osc.: 0.162

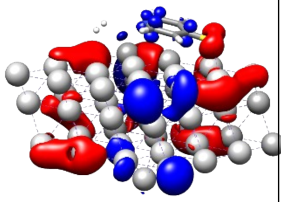

State 173
Energy: 2.979 eV
Osc.: 0.078

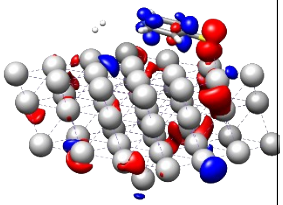

State 174
Energy: 2.982 eV
Osc.: 0.178

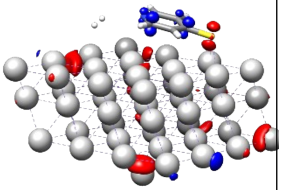

State 175
Energy: 2.987 eV
Osc.: 0.023

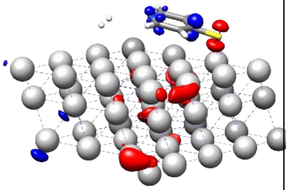

State 176
Energy: 3.000 eV
Osc.: 0.209

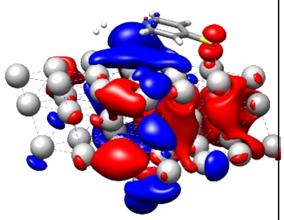

State 178
Energy: 3.019 eV
Osc.: 0.174

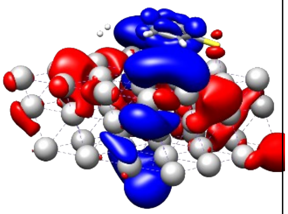

State 179
Energy: 3.027 eV
Osc.: 0.363

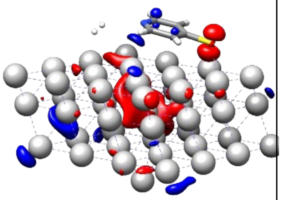

State 180
Energy: 3.034 eV
Osc.: 0.136

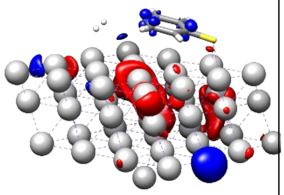

State 181
Energy: 3.048 eV
Osc.: 0.012

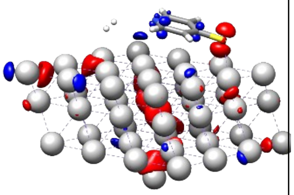

State 182
Energy: 3.056 eV
Osc.: 0.089

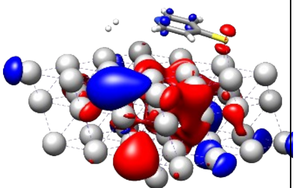

State 183
Energy: 3.064 eV
Osc.: 0.445

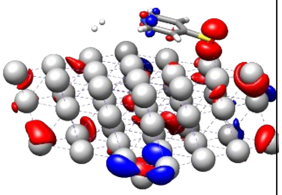

State 184
Energy: 3.071 eV
Osc.: 0.080

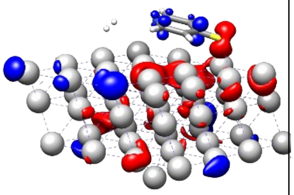

State 185
Energy: 3.079 eV
Osc.: 0.034

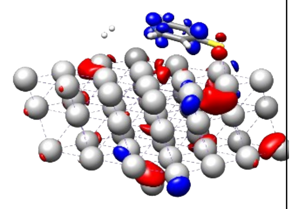<br>State 186<br>Energy: 3.088 eV<br>Osc.: 0.072	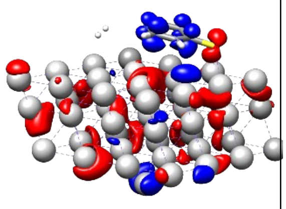<br>State 187<br>Energy: 3.096 eV<br>Osc.: 0.070	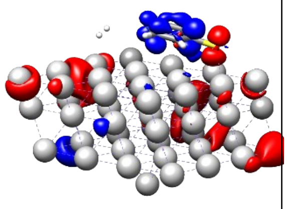<br>State 188<br>Energy: 3.103 eV<br>Osc.: 0.177	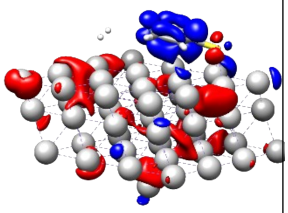<br>State 189<br>Energy: 3.106 eV<br>Osc.: 0.186
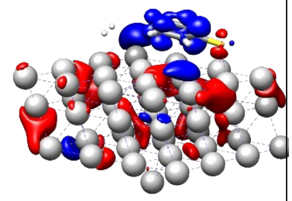<br>State 190<br>Energy: 3.118 eV<br>Osc.: 0.475	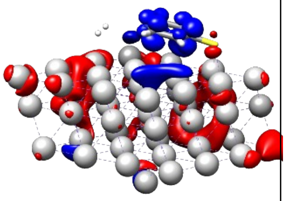<br>State 191<br>Energy: 3.120 eV<br>Osc.: 0.037	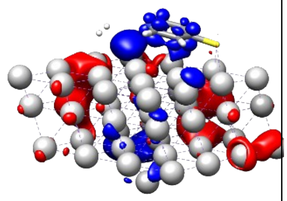<br>State 192<br>Energy: 3.127 eV<br>Osc.: 0.017	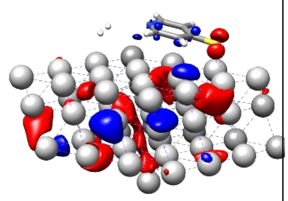<br>State 193<br>Energy: 3.130 eV<br>Osc.: 0.609
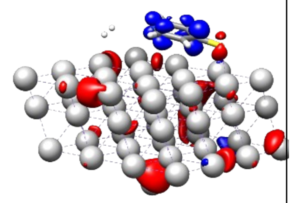<br>State 195<br>Energy: 3.152 eV<br>Osc.: 0.326	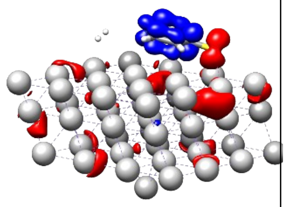<br>State 196<br>Energy: 3.157 eV<br>Osc.: 0.083	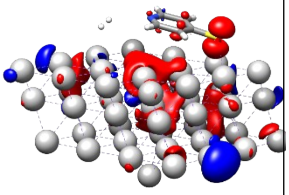<br>State 197<br>Energy: 3.166 eV<br>Osc.: 0.033	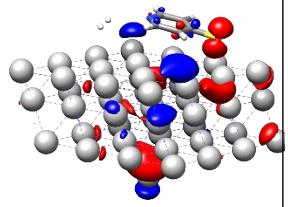<br>State 198<br>Energy: 3.173 eV<br>Osc.: 0.281
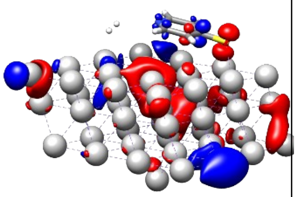<br>State 199<br>Energy: 3.180 eV<br>Osc.: 0.020	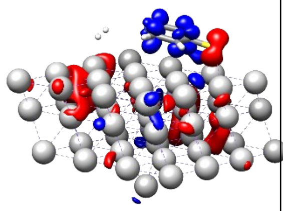<br>State 200<br>Energy: 3.182 eV<br>Osc.: 0.056	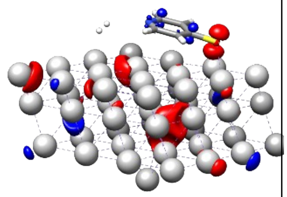<br>State 201<br>Energy: 3.196 eV<br>Osc.: 0.121	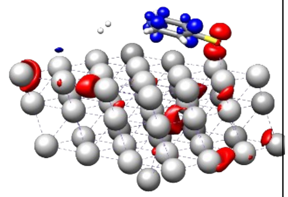<br>State 202<br>Energy: 3.202 eV<br>Osc.: 0.012

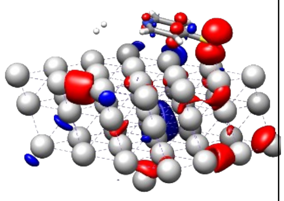<br>State 203<br>Energy: 3.208 eV<br>Osc.: 0.034	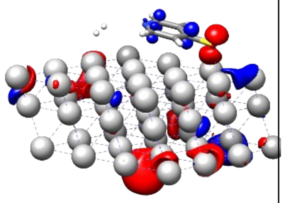<br>State 204<br>Energy: 3.217 eV<br>Osc.: 0.023	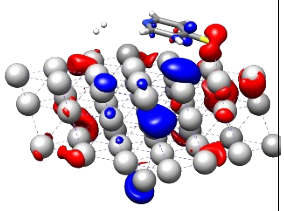<br>State 205<br>Energy: 3.227 eV<br>Osc.: 0.169	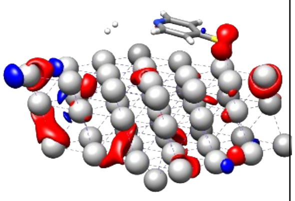<br>State 206<br>Energy: 3.230 eV<br>Osc.: 0.016
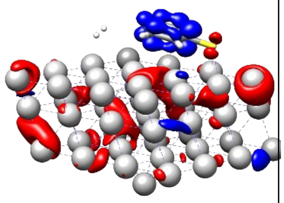<br>State 207<br>Energy: 3.236 eV<br>Osc.: 0.028	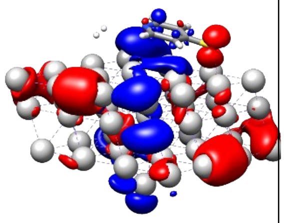<br>State 208<br>Energy: 3.242 eV<br>Osc.: 0.027	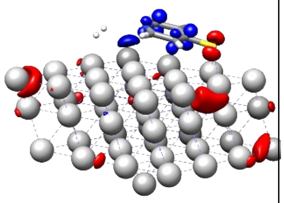<br>State 209<br>Energy: 3.247 eV<br>Osc.: 0.015	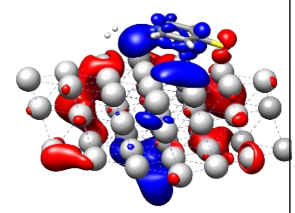<br>State 210<br>Energy: 3.252 eV<br>Osc.: 0.024
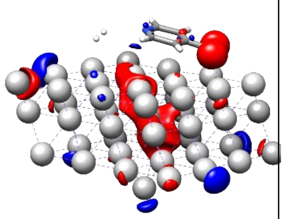<br>State 211<br>Energy: 3.260 eV<br>Osc.: 0.042	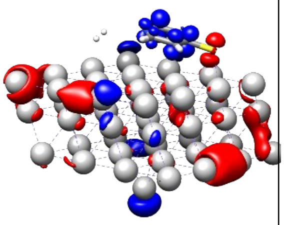<br>State 213<br>Energy: 3.277 eV<br>Osc.: 0.018	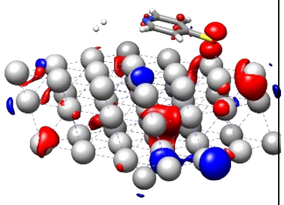<br>State 214<br>Energy: 3.281 eV<br>Osc.: 0.157	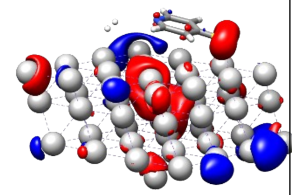<br>State 215<br>Energy: 3.284 eV<br>Osc.: 0.015
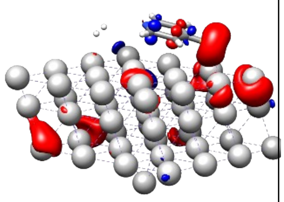<br>State 216<br>Energy: 3.292 eV<br>Osc.: 0.392	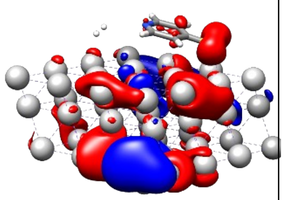<br>State 217<br>Energy: 3.297 eV<br>Osc.: 0.031	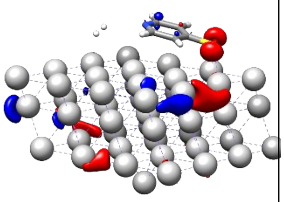<br>State 220<br>Energy: 3.312 eV<br>Osc.: 0.152	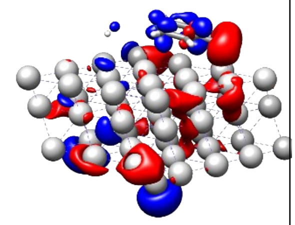<br>State 222<br>Energy: 3.324 eV<br>Osc.: 0.037

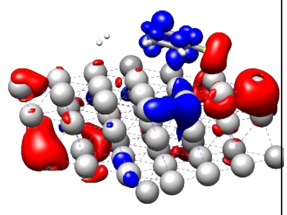<br>State 223<br>Energy: 3.334 eV<br>Osc.: 0.151	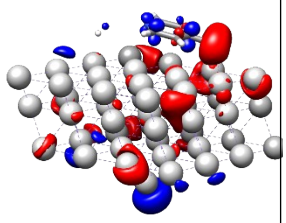<br>State 224<br>Energy: 3.335 eV<br>Osc.: 0.056		

Table S5. Charge density differences (CDDs) illustrating the nature of the low-lying bright excitations of two molecules in structure 2 interacting with an H_2 molecule on Ag surface. Charge transfer takes place from red to blue.

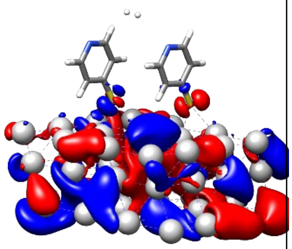<br>State 48<br>Energy: 1.636 eV<br>Osc.: 0.010	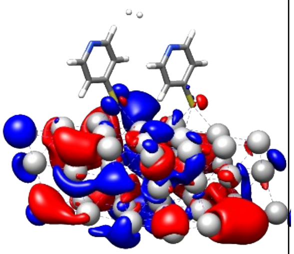<br>State 49<br>Energy: 1.653 eV<br>Osc.: 0.012	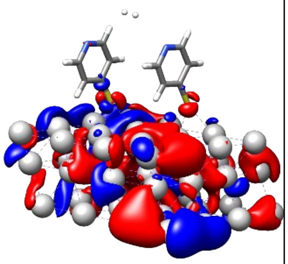<br>State 54<br>Energy: 1.742 eV<br>Osc.: 0.022	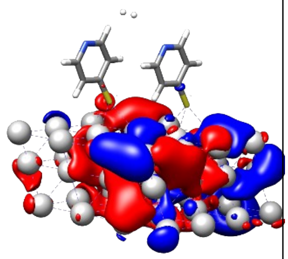<br>State 56<br>Energy: 1.762 eV<br>Osc.: 0.010
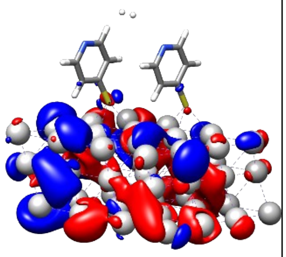<br>State 58<br>Energy: 1.789 eV<br>Osc.: 0.015	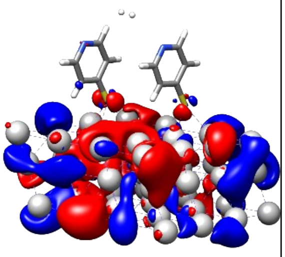<br>State 63<br>Energy: 1.846 eV<br>Osc.: 0.018	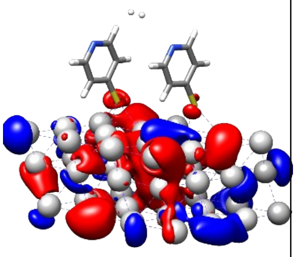<br>State 64<br>Energy: 1.869 eV<br>Osc.: 0.010	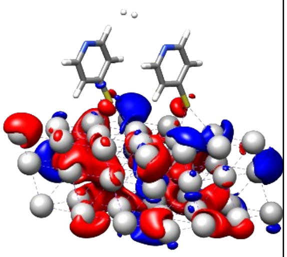<br>State 68<br>Energy: 1.920 eV<br>Osc.: 0.011
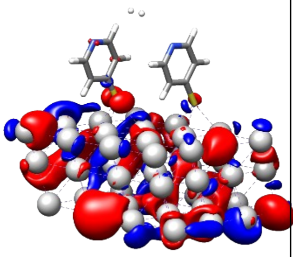<br>State 71<br>Energy: 1.958 eV<br>Osc.: 0.018	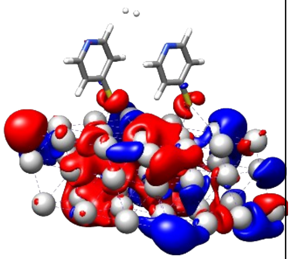<br>State 73<br>Energy: 1.995 eV<br>Osc.: 0.010	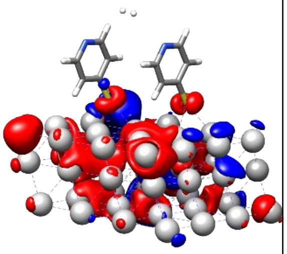<br>State 74<br>Energy: 2.008 eV<br>Osc.: 0.013	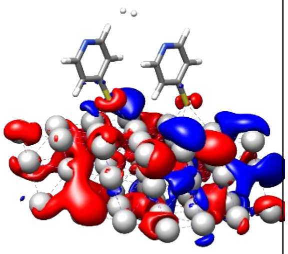<br>State 77<br>Energy: 2.044 eV<br>Osc.: 0.010

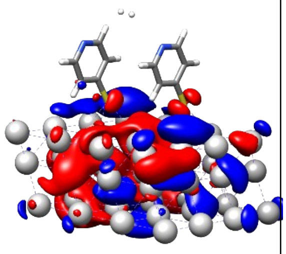

State 83
Energy: 2.136 eV
Osc.: 0.023

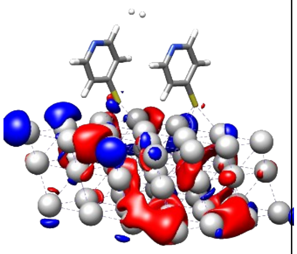

State 85
Energy: 2.176 eV
Osc.: 0.017

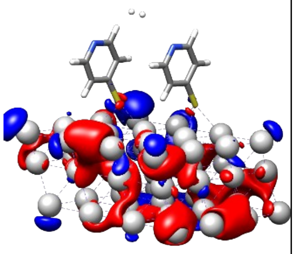

State 87
Energy: 2.204 eV
Osc.: 0.014

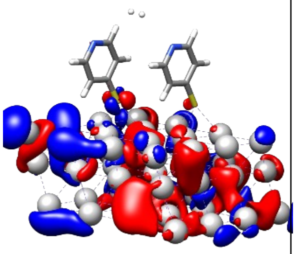

State 89
Energy: 2.231 eV
Osc.: 0.000

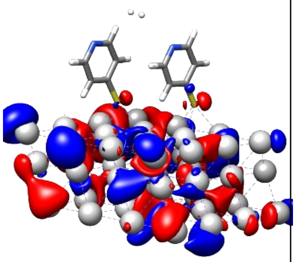

State 90
Energy: 2.236 eV
Osc.: 0.031

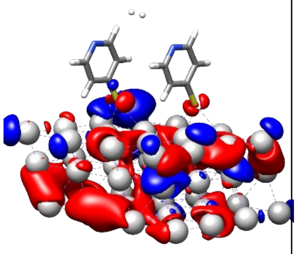

State 92
Energy: 2.257 eV
Osc.: 0.019

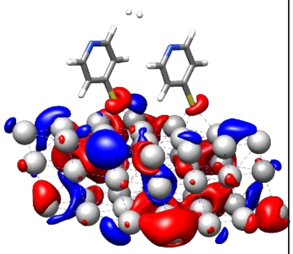

State 93
Energy: 2.261 eV
Osc.: 0.011

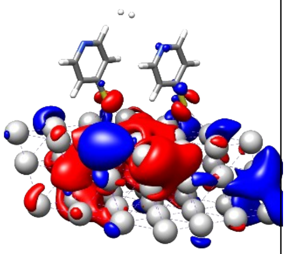

State 95
Energy: 2.290 eV
Osc.: 0.025

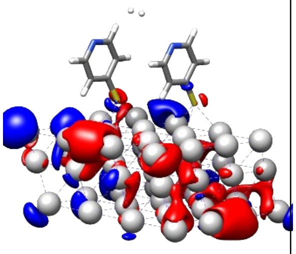

State 98
Energy: 2.332 eV
Osc.: 0.011

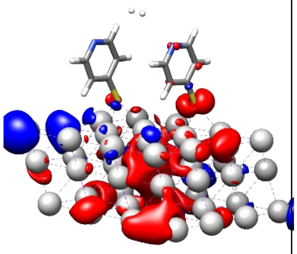

State 99
Energy: 2.344 eV
Osc.: 0.024

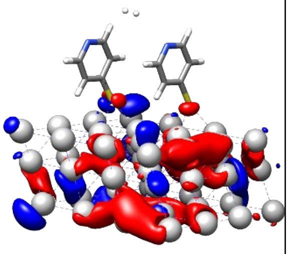

State 101
Energy: 2.370 eV
Osc.: 0.053

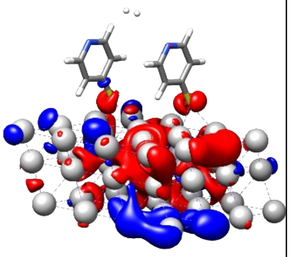

State 109
Energy: 2.458 eV
Osc.: 0.026

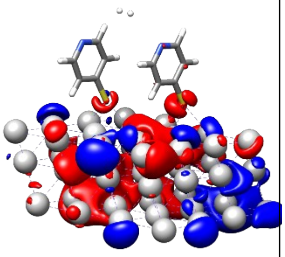

State 110
Energy: 2.494 eV
Osc.: 0.010

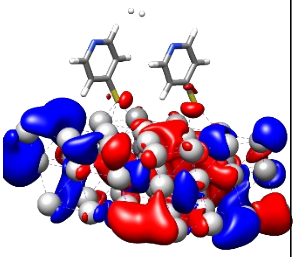

State 111
Energy: 2.508 eV
Osc.: 0.010

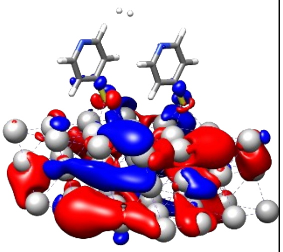

State 112
Energy: 2.514 eV
Osc.: 0.021

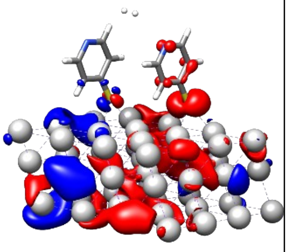

State 113
Energy: 2.528 eV
Osc.: 0.020

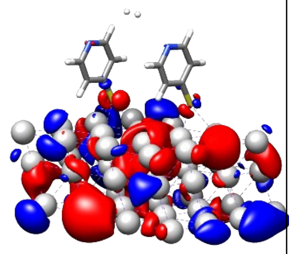<br>State 115<br>Energy: 2.543 eV<br>Osc.: 0.026	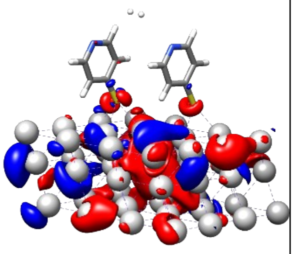<br>State 116<br>Energy: 2.546 eV<br>Osc.: 0.044	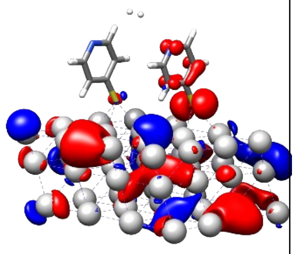<br>State 119<br>Energy: 2.578 eV<br>Osc.: 0.074	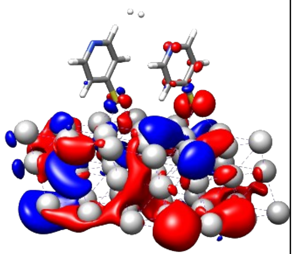<br>State 120<br>Energy: 2.581 eV<br>Osc.: 0.012
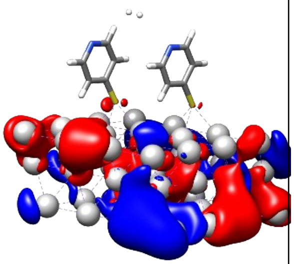<br>State 122<br>Energy: 2.612 eV<br>Osc.: 0.024	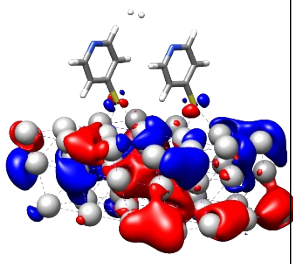<br>State 124<br>Energy: 2.623 eV<br>Osc.: 0.012	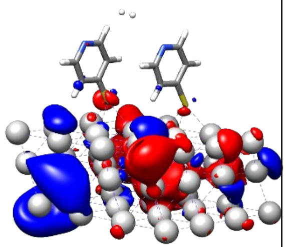<br>State 125<br>Energy: 2.638 eV<br>Osc.: 0.014	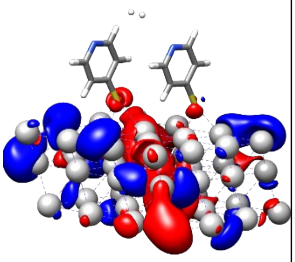<br>State 127<br>Energy: 2.645 eV<br>Osc.: 0.021
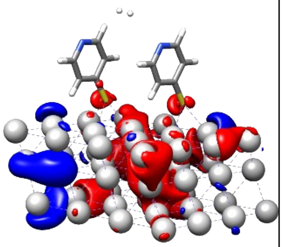<br>State 128<br>Energy: 2.655 eV<br>Osc.: 0.045	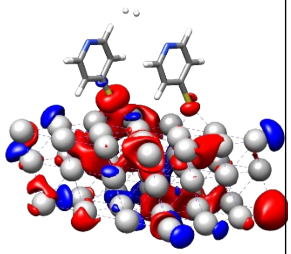<br>State 130<br>Energy: 2.672 eV<br>Osc.: 0.051	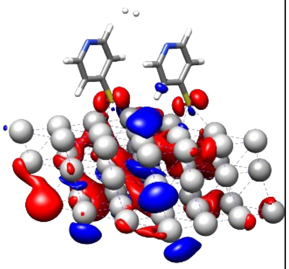<br>State 131<br>Energy: 2.676 eV<br>Osc.: 0.030	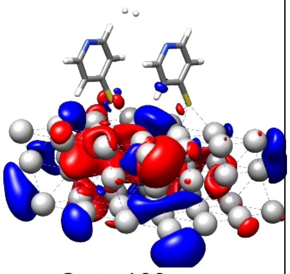<br>State 132<br>Energy: 2.685 eV<br>Osc.: 0.028
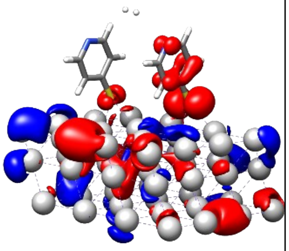<br>State 133<br>Energy: 2.701 eV<br>Osc.: 0.015	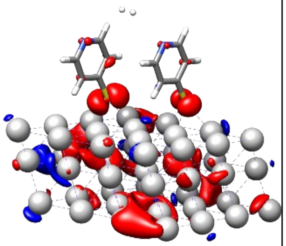<br>State 134<br>Energy: 2.719 eV<br>Osc.: 0.015	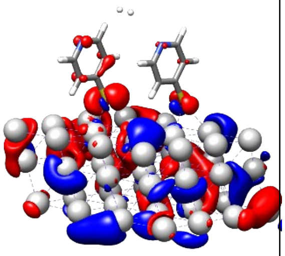<br>State 135<br>Energy: 2.725 eV<br>Osc.: 0.011	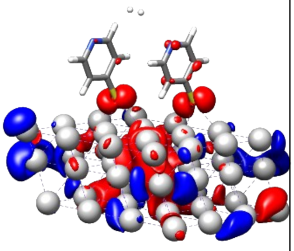<br>State 136<br>Energy: 2.740 eV<br>Osc.: 0.025

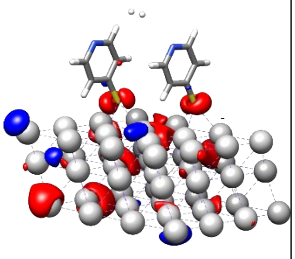<br>State 137<br>Energy: 2.747 eV<br>Osc.: 0.019	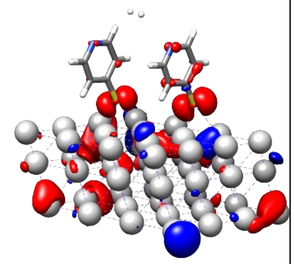<br>State 139<br>Energy: 2.771 eV<br>Osc.: 0.028	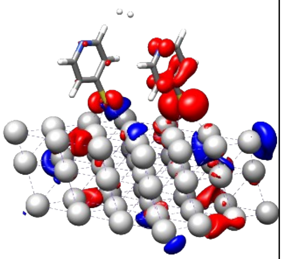<br>State 140<br>Energy: 2.775 eV<br>Osc.: 0.020	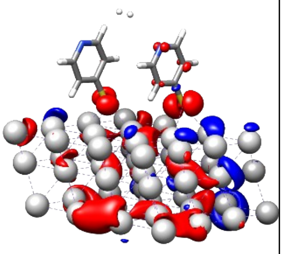<br>State 141<br>Energy: 2.781 eV<br>Osc.: 0.019
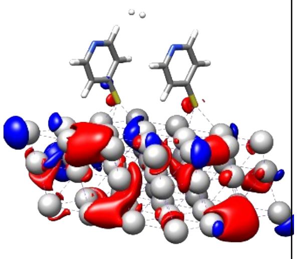<br>State 142<br>Energy: 2.791 eV<br>Osc.: 0.012	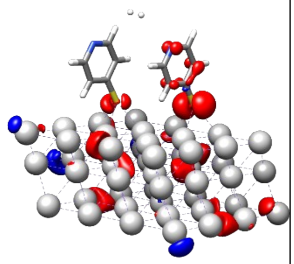<br>State 144<br>Energy: 2.809 eV<br>Osc.: 0.053	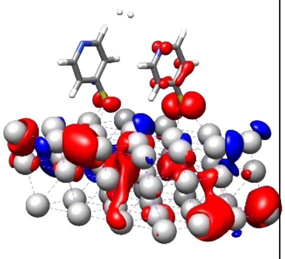<br>State 145<br>Energy: 2.817 eV<br>Osc.: 0.013	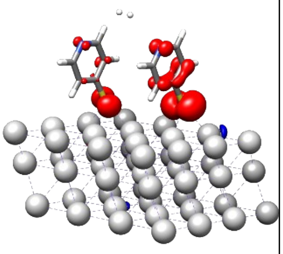<br>State 146<br>Energy: 2.831 eV<br>Osc.: 1.473
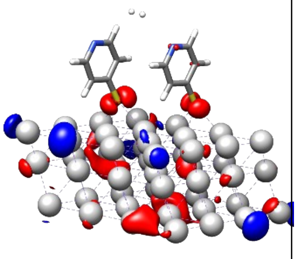<br>State 147<br>Energy: 2.847 eV<br>Osc.: 0.022	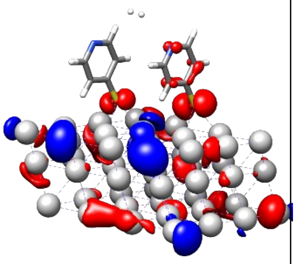<br>State 148<br>Energy: 2.857 eV<br>Osc.: 0.046	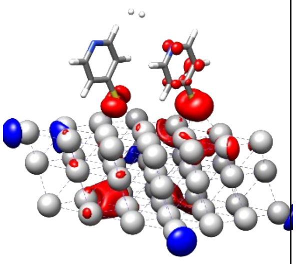<br>State 150<br>Energy: 2.870 eV<br>Osc.: 0.486	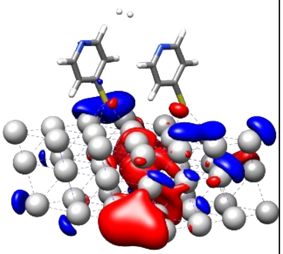<br>State 151<br>Energy: 2.876 eV<br>Osc.: 0.257
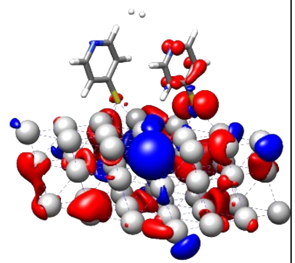<br>State 152<br>Energy: 2.888 eV<br>Osc.: 0.017	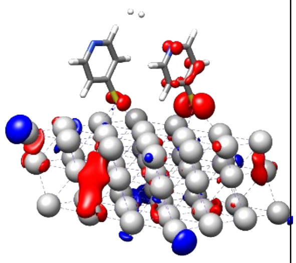<br>State 153<br>Energy: 2.895 eV<br>Osc.: 0.311	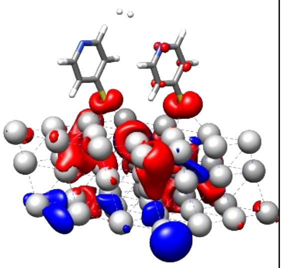<br>State 154<br>Energy: 2.910 eV<br>Osc.: 0.070	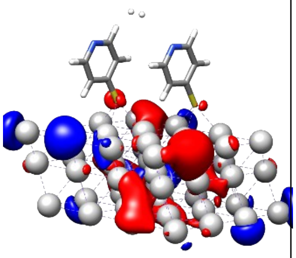<br>State 155<br>Energy: 2.925 eV<br>Osc.: 0.083

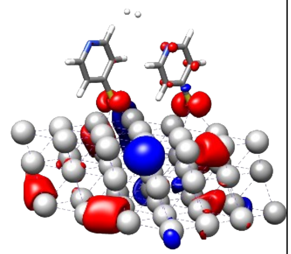<br>State 156<br>Energy: 2.931 eV<br>Osc.: 0.021	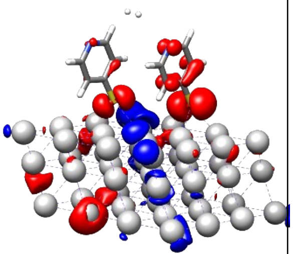<br>State 157<br>Energy: 2.932 eV<br>Osc.: 0.101	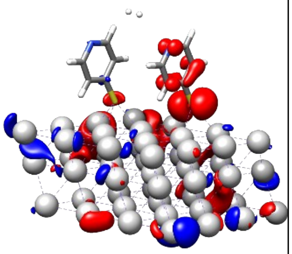<br>State 158<br>Energy: 2.942 eV<br>Osc.: 0.192	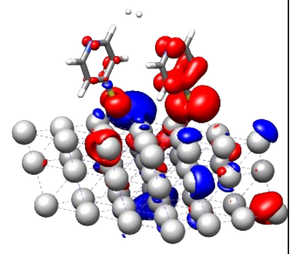<br>State 159<br>Energy: 2.950 eV<br>Osc.: 0.013
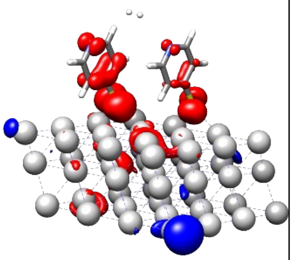<br>State 160<br>Energy: 2.957 eV<br>Osc.: 0.062	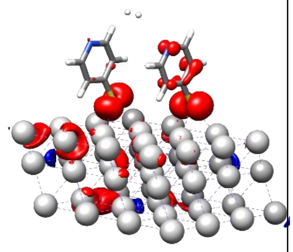<br>State 162<br>Energy: 2.972 eV<br>Osc.: 0.032	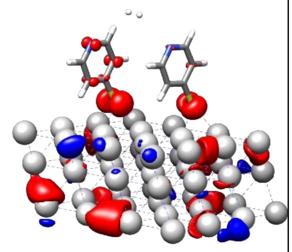<br>State 163<br>Energy: 2.977 eV<br>Osc.: 0.311	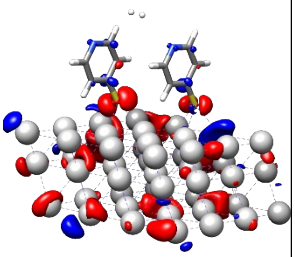<br>State 164<br>Energy: 2.986 eV<br>Osc.: 0.040
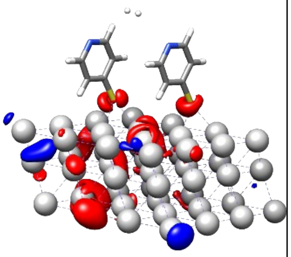<br>State 165<br>Energy: 3.001 eV<br>Osc.: 0.011	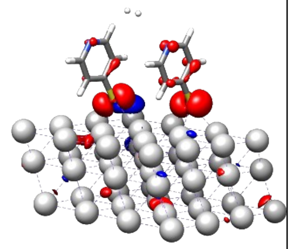<br>State 166<br>Energy: 3.004 eV<br>Osc.: 0.193	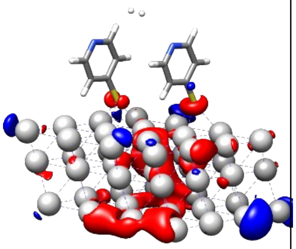<br>State 167<br>Energy: 3.007 eV<br>Osc.: 0.054	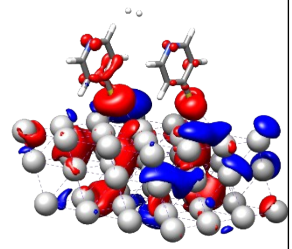<br>State 169<br>Energy: 3.026 eV<br>Osc.: 0.092
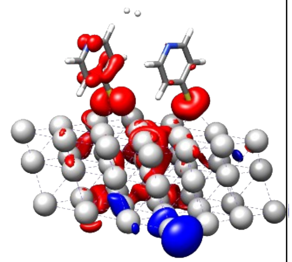<br>State 170<br>Energy: 3.029 eV<br>Osc.: 0.131	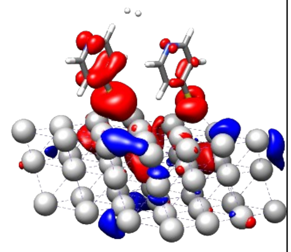<br>State 171<br>Energy: 3.036 eV<br>Osc.: 0.160	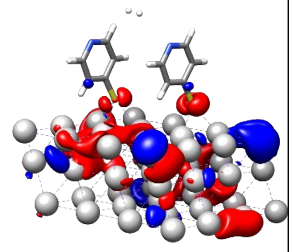<br>State 172<br>Energy: 3.043 eV<br>Osc.: 0.138	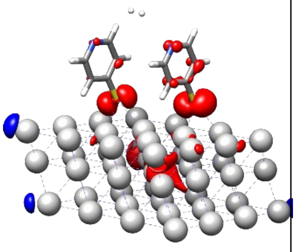<br>State 173<br>Energy: 3.049 eV<br>Osc.: 0.026

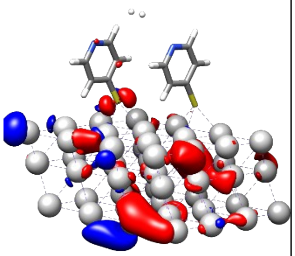<br>State 174<br>Energy: 3.061 eV<br>Osc.: 0.071	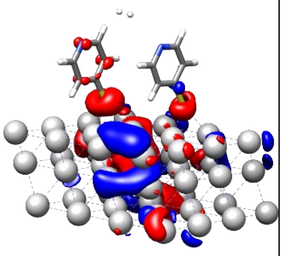<br>State 175<br>Energy: 3.065 eV<br>Osc.: 0.251	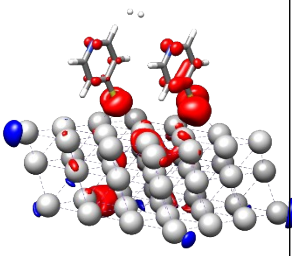<br>State 177<br>Energy: 3.083 eV<br>Osc.: 0.106	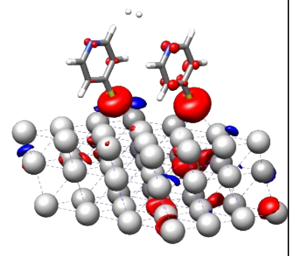<br>State 178<br>Energy: 3.097 eV<br>Osc.: 0.026
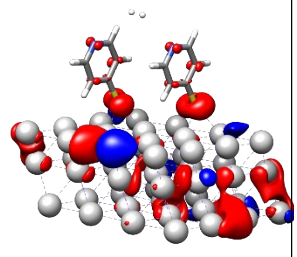<br>State 179<br>Energy: 3.097 eV<br>Osc.: 0.209	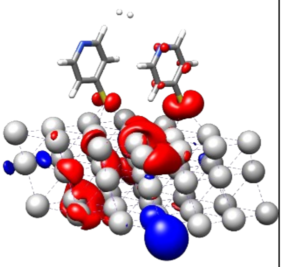<br>State 180<br>Energy: 3.105 eV<br>Osc.: 0.019	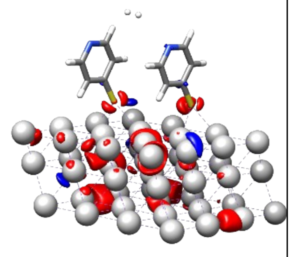<br>State 181<br>Energy: 3.117 eV<br>Osc.: 0.063	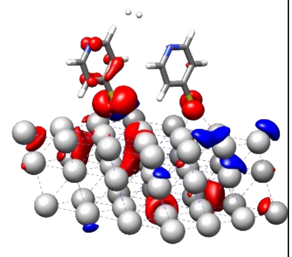<br>State 182<br>Energy: 3.127 eV<br>Osc.: 0.275
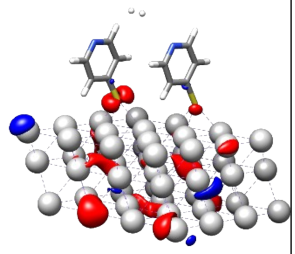<br>State 183<br>Energy: 3.136 eV<br>Osc.: 0.033	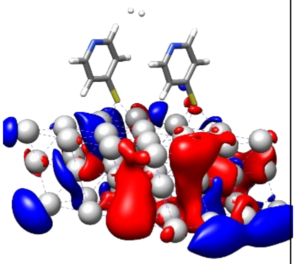<br>State 184<br>Energy: 3.146 eV<br>Osc.: 0.013	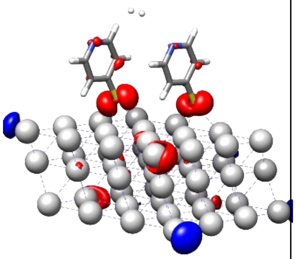<br>State 185<br>Energy: 3.149 eV<br>Osc.: 0.147	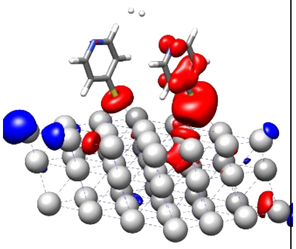<br>State 186<br>Energy: 3.155 eV<br>Osc.: 0.158
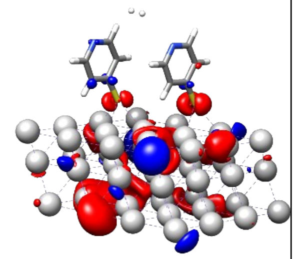<br>State 187<br>Energy: 3.159 eV<br>Osc.: 0.025	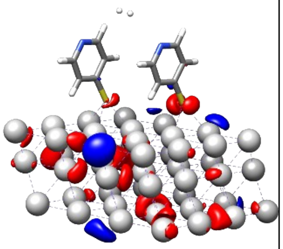<br>State 188<br>Energy: 3.177 eV<br>Osc.: 0.144	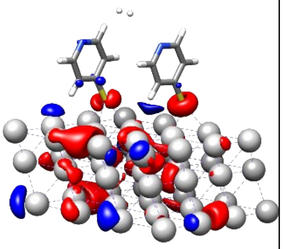<br>State 189<br>Energy: 3.181 eV<br>Osc.: 0.030	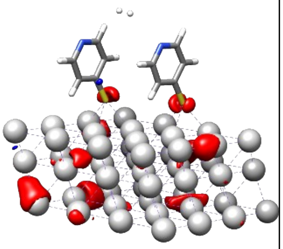<br>State 191<br>Energy: 3.191 eV<br>Osc.: 0.073

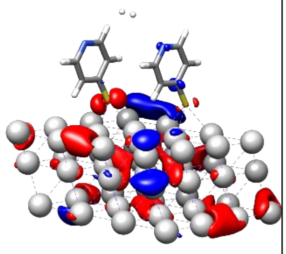

State 192
Energy: 3.195 eV
Osc.: 0.092

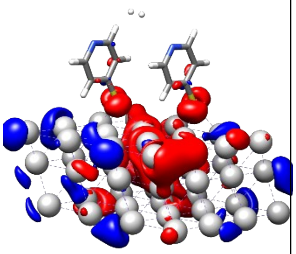

State 193
Energy: 3.200 eV
Osc.: 0.039

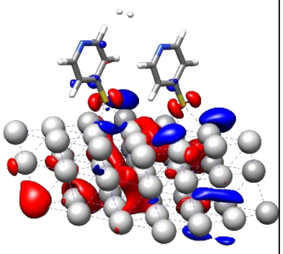

State 194
Energy: 3.209 eV
Osc.: 0.113

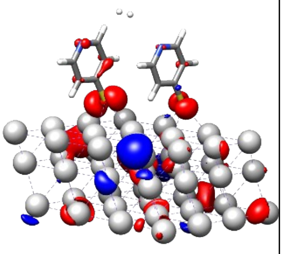

State 195
Energy: 3.213 eV
Osc.: 0.038

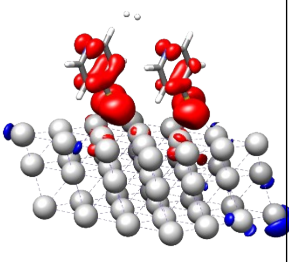

State 196
Energy: 3.222 eV
Osc.: 0.229

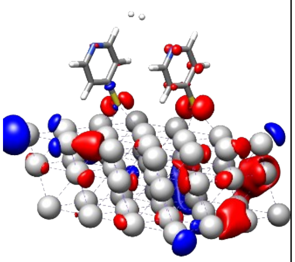

State 197
Energy: 3.228 eV
Osc.: 0.095

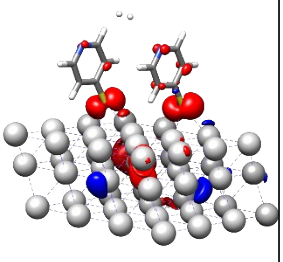

State 198
Energy: 3.236 eV
Osc.: 0.035

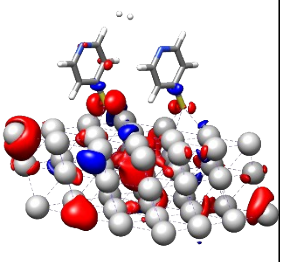

State 199
Energy: 3.239 eV
Osc.: 0.012

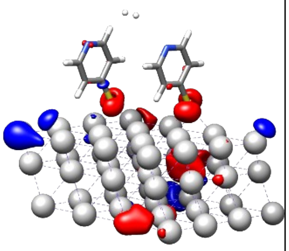

State 200
Energy: 3.247 eV
Osc.: 0.012

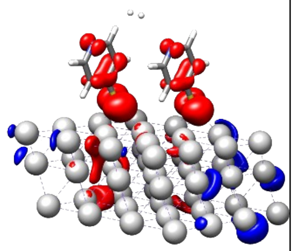

State 202
Energy: 3.252 eV
Osc.: 0.011

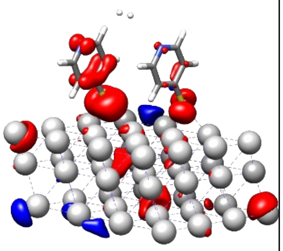

State 203
Energy: 3.268 eV
Osc.: 0.130

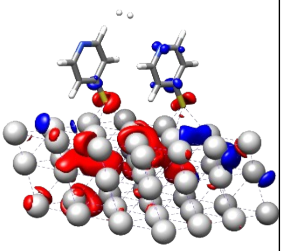

State 204
Energy: 3.275 eV
Osc.: 0.012

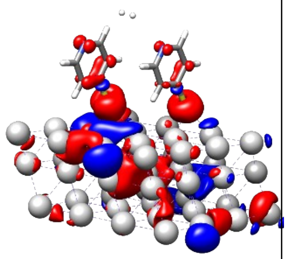

State 205
Energy: 3.283 eV
Osc.: 0.037

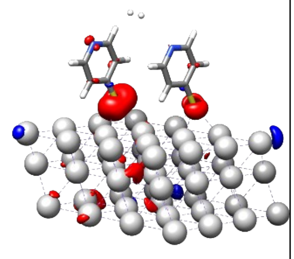

State 206
Energy: 3.289 eV
Osc.: 0.299

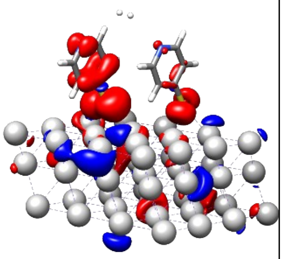

State 207
Energy: 3.292 eV
Osc.: 0.024

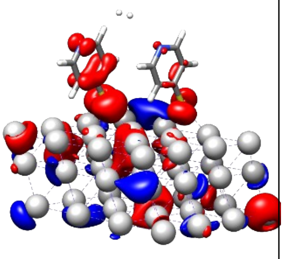

State 208
Energy: 3.301 eV
Osc.: 0.018

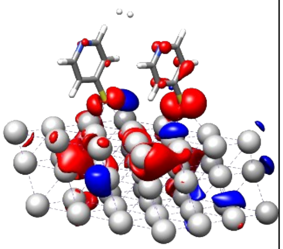<br>State 209<br>Energy: 3.305 eV<br>Osc.: 0.050			

Table S6. Charge density differences (CDDs) illustrating the nature of the low-lying bright excitations of two molecules in structure 3 interacting with an H_2 molecule on Ag surface. Charge transfer takes place from red to blue.

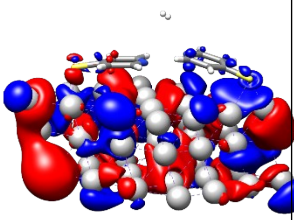<br>State 40<br>Energy: 1.480 eV<br>Osc.: 0.014	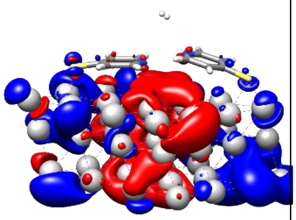<br>State 42<br>Energy: 1.530 eV<br>Osc.: 0.016	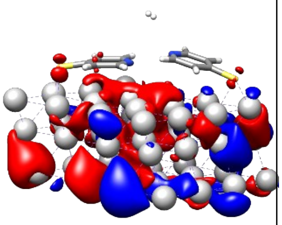<br>State 44<br>Energy: 1.557 eV<br>Osc.: 0.022	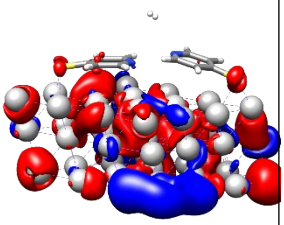<br>State 52<br>Energy: 1.689 eV<br>Osc.: 0.011
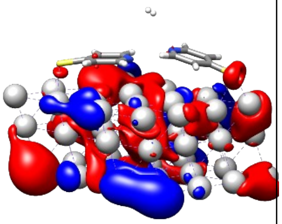<br>State 56<br>Energy: 1.726 eV<br>Osc.: 0.012	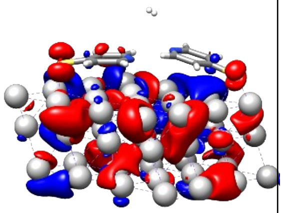<br>State 61<br>Energy: 1.841 eV<br>Osc.: 0.047	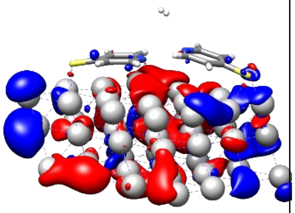<br>State 66<br>Energy: 1.903 eV<br>Osc.: 0.038	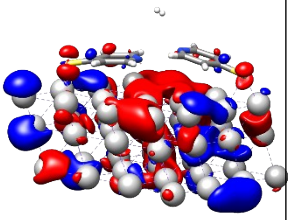<br>State 67<br>Energy: 1.912 eV<br>Osc.: 0.011
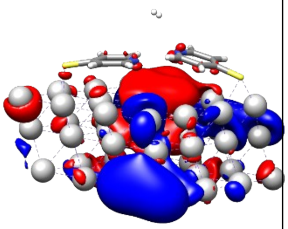<br>State 68<br>Energy: 1.927 eV<br>Osc.: 0.017	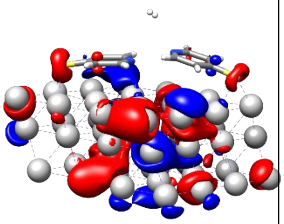<br>State 72<br>Energy: 1.975 eV<br>Osc.: 0.016	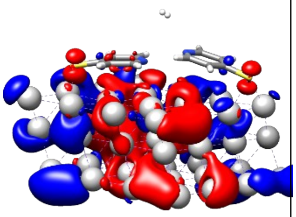<br>State 79<br>Energy: 2.068 eV<br>Osc.: 0.012	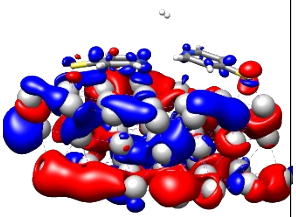<br>State 80<br>Energy: 2.085 eV<br>Osc.: 0.019

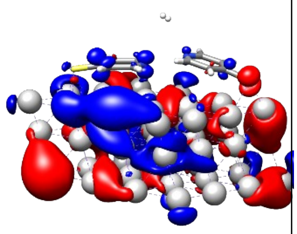

State 81
Energy: 2.094 eV
Osc.: 0.019

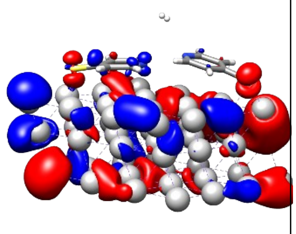

State 83
Energy: 2.140 eV
Osc.: 0.017

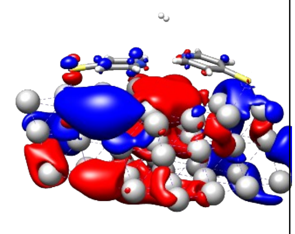

State 89
Energy: 2.210 eV
Osc.: 0.019

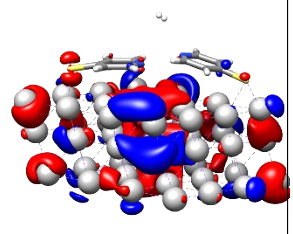

State 92
Energy: 2.246 eV
Osc.: 0.039

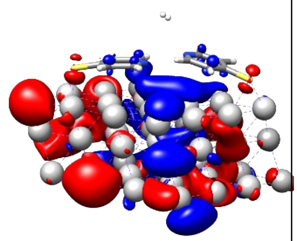

State 93
Energy: 2.261 eV
Osc.: 0.011

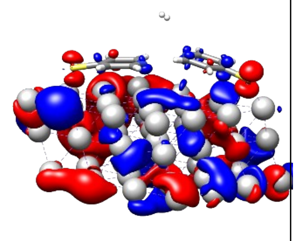

State 99
Energy: 2.314 eV
Osc.: 0.029

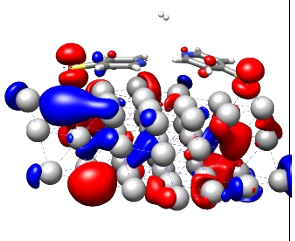

State 101
Energy: 2.345 eV
Osc.: 0.067

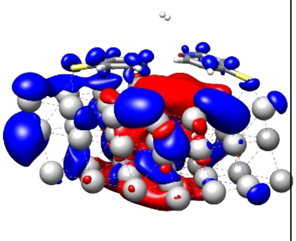

State 104
Energy: 2.373 eV
Osc.: 0.034

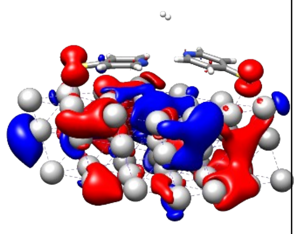

State 105
Energy: 2.387 eV
Osc.: 0.049

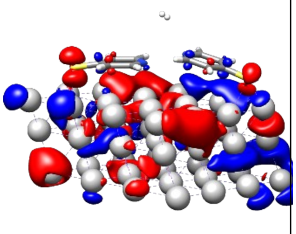

State 107
Energy: 2.412 eV
Osc.: 0.022

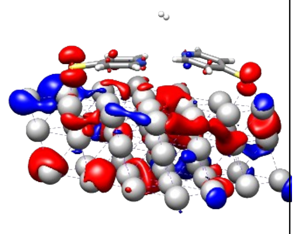

State 110
Energy: 2.456 eV
Osc.: 0.010

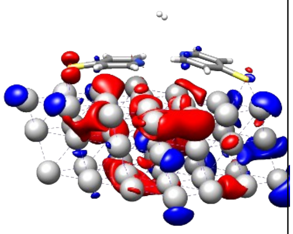

State 112
Energy: 2.464 eV
Osc.: 0.020

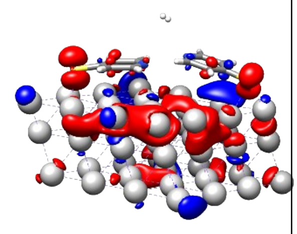

State 114
Energy: 2.482 eV
Osc.: 0.050

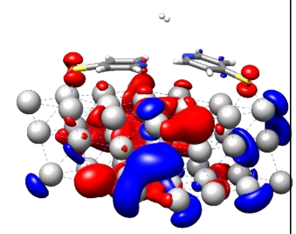

State 115
Energy: 2.493 eV
Osc.: 0.030

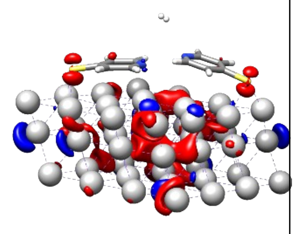

State 116
Energy: 2.510 eV
Osc.: 0.069

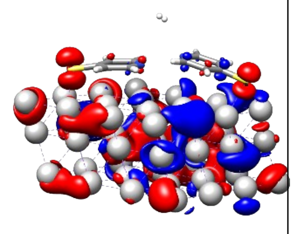

State 117
Energy: 2.514 eV
Osc.: 0.040

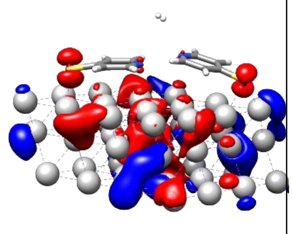

State 118
Energy: 2.522 eV
Osc.: 0.010

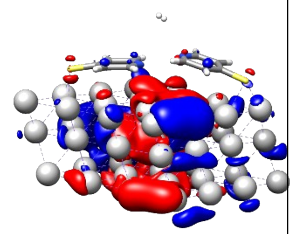

State 119
Energy: 2.526 eV
Osc.: 0.020

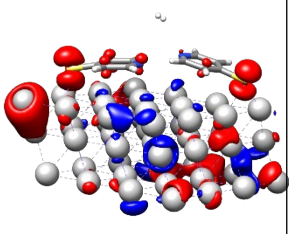

State 120
Energy: 2.542 eV
Osc.: 0.012

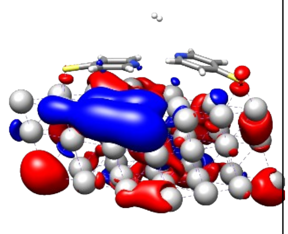

State 121
Energy: 2.552 eV
Osc.: 0.017

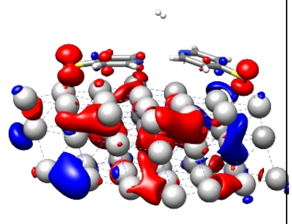

State 122
Energy: 2.564 eV
Osc.: 0.037

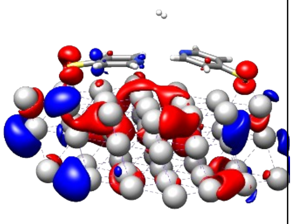

State 125
Energy: 2.583 eV
Osc.: 0.039

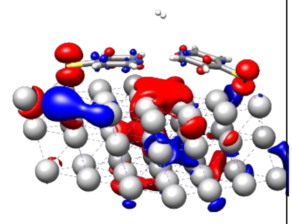

State 126
Energy: 2.587 eV
Osc.: 0.013

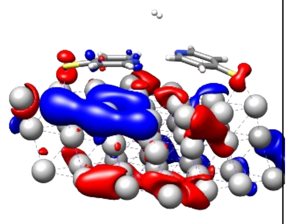

State 127
Energy: 2.599 eV
Osc.: 0.020

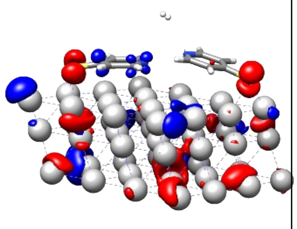

State 128
Energy: 2.612 eV
Osc.: 0.115

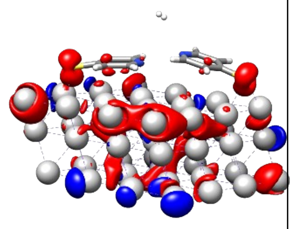

State 129
Energy: 2.626 eV
Osc.: 0.085

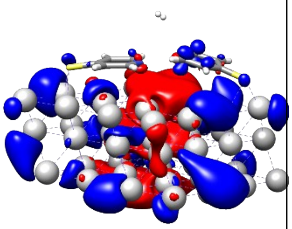

State 130
Energy: 2.640 eV
Osc.: 0.023

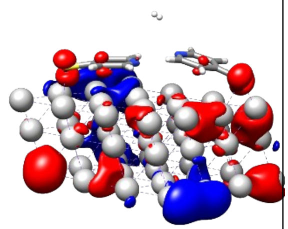

State 131
Energy: 2.656 eV
Osc.: 0.010

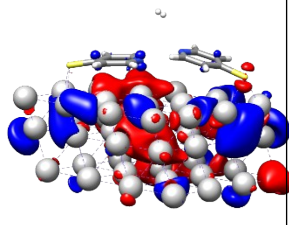

State 132
Energy: 2.664 eV
Osc.: 0.182

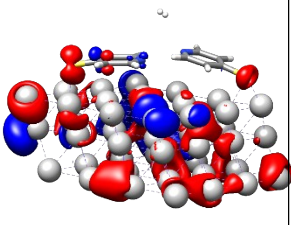

State 133
Energy: 2.666 eV
Osc.: 0.019

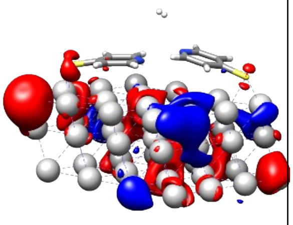

State 134
Energy: 2.682 eV
Osc.: 0.150

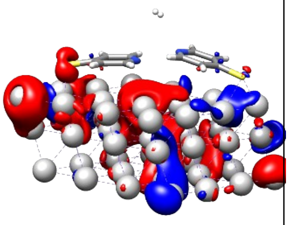

State 135
Energy: 2.684 eV
Osc.: 0.014

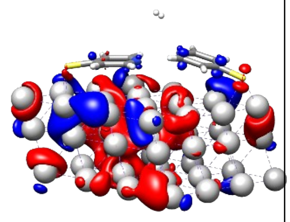<br>State 137<br>Energy: 2.703 eV<br>Osc.: 0.075	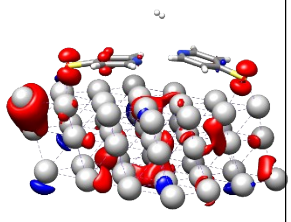<br>State 138<br>Energy: 2.716 eV<br>Osc.: 0.056	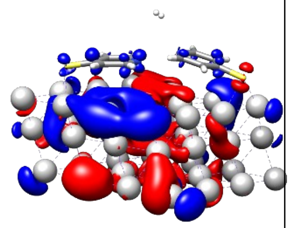<br>State 139<br>Energy: 2.734 eV<br>Osc.: 0.045	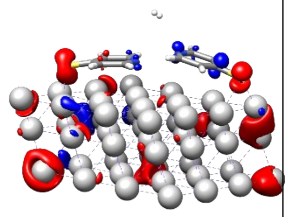<br>State 140<br>Energy: 2.743 eV<br>Osc.: 0.029
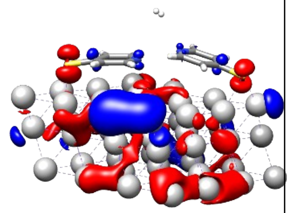<br>State 141<br>Energy: 2.746 eV<br>Osc.: 0.063	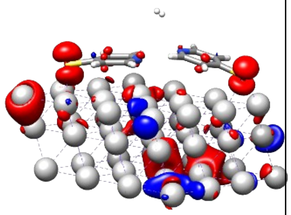<br>State 142<br>Energy: 2.753 eV<br>Osc.: 0.035	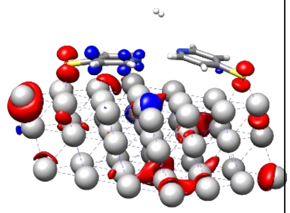<br>State 143<br>Energy: 2.765 eV<br>Osc.: 0.032	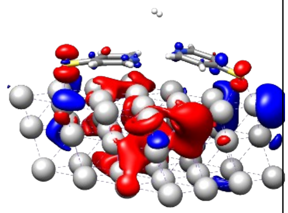<br>State 144<br>Energy: 2.780 eV<br>Osc.: 0.011
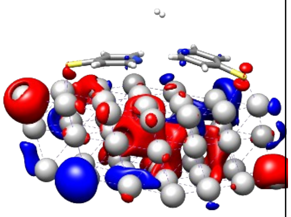<br>State 145<br>Energy: 2.789 eV<br>Osc.: 0.027	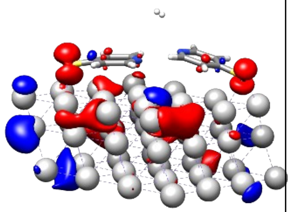<br>State 146<br>Energy: 2.795 eV<br>Osc.: 0.049	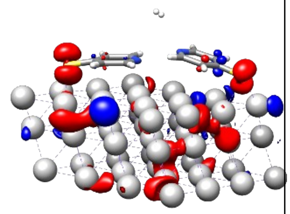<br>State 147<br>Energy: 2.799 eV<br>Osc.: 0.323	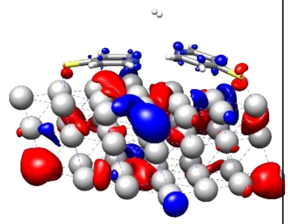<br>State 151<br>Energy: 2.850 eV<br>Osc.: 0.022
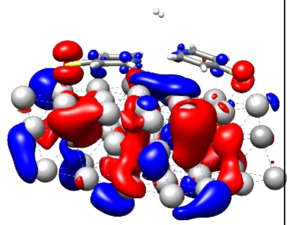<br>State 152<br>Energy: 2.857 eV<br>Osc.: 0.077	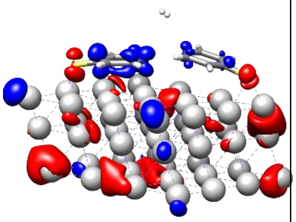<br>State 153<br>Energy: 2.865 eV<br>Osc.: 0.258	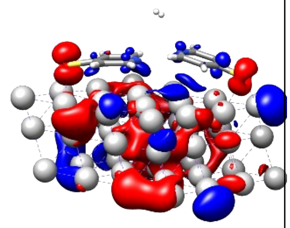<br>State 154<br>Energy: 2.878 eV<br>Osc.: 0.020	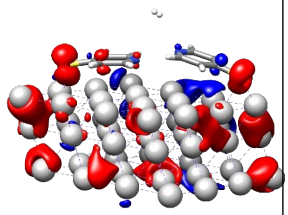<br>State 157<br>Energy: 2.890 eV<br>Osc.: 0.011

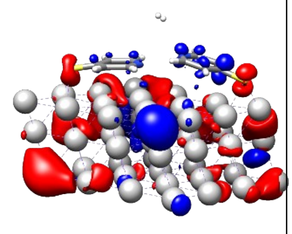

State 158
Energy: 2.907 eV
Osc.: 0.150

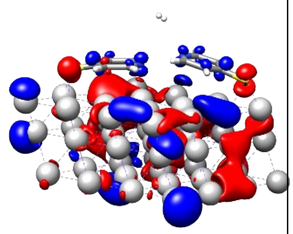

State 159
Energy: 2.912 eV
Osc.: 0.133

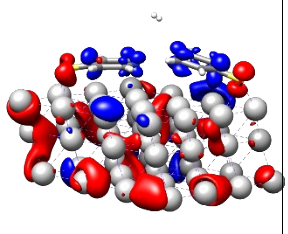

State 160
Energy: 2.918 eV
Osc.: 0.029

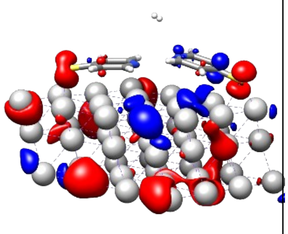

State 161
Energy: 2.927 eV
Osc.: 0.157

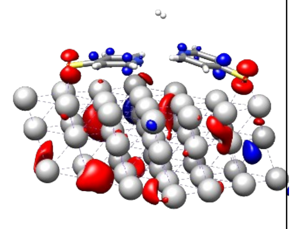

State 162
Energy: 2.936 eV
Osc.: 0.165

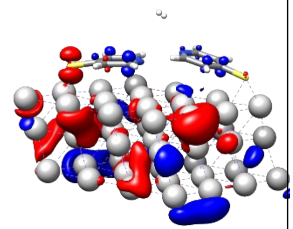

State 163
Energy: 2.942 eV
Osc.: 0.190

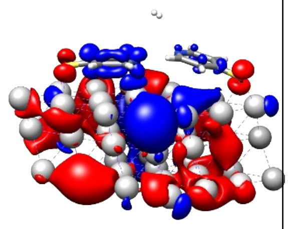

State 164
Energy: 2.954 eV
Osc.: 0.032

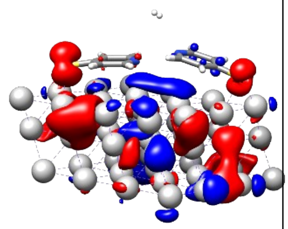

State 165
Energy: 2.967 eV
Osc.: 0.226

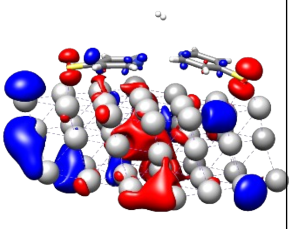

State 166
Energy: 2.982 eV
Osc.: 0.397

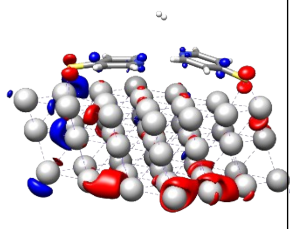

State 167
Energy: 2.984 eV
Osc.: 0.069

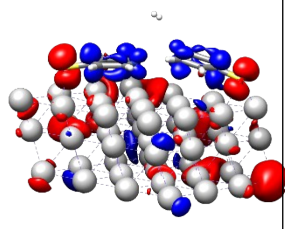

State 168
Energy: 2.994 eV
Osc.: 0.167

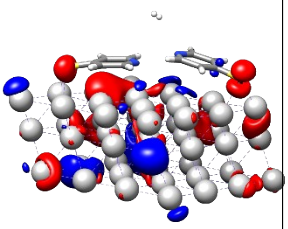

State 171
Energy: 3.015 eV
Osc.: 0.176

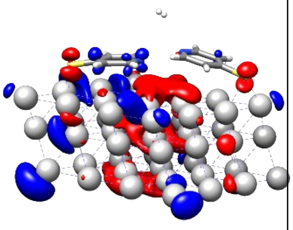

State 172
Energy: 3.021 eV
Osc.: 0.112

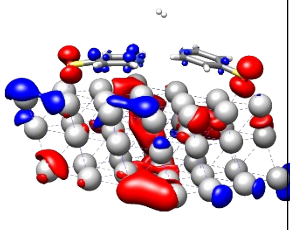

State 173
Energy: 3.031 eV
Osc.: 0.016

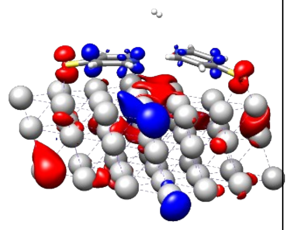

State 175
Energy: 3.048 eV
Osc.: 0.029

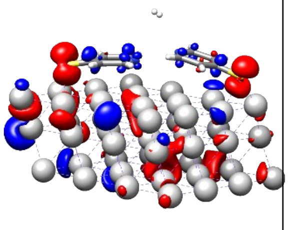

State 176
Energy: 3.068 eV
Osc.: 0.291

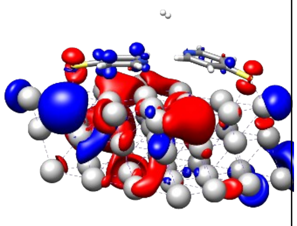<br>State 177<br>Energy: 3.073 eV<br>Osc.: 0.088	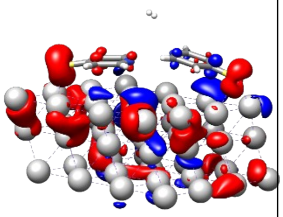<br>State 178<br>Energy: 3.078 eV<br>Osc.: 0.067	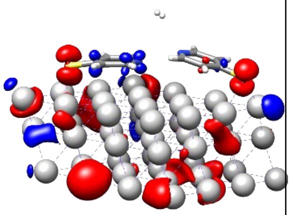<br>State 179<br>Energy: 3.090 eV<br>Osc.: 0.236	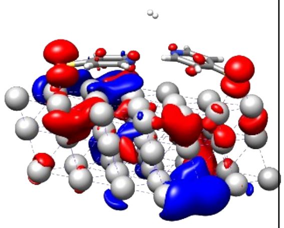<br>State 181<br>Energy: 3.098 eV<br>Osc.: 0.027
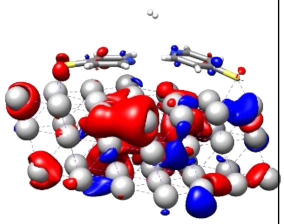<br>State 182<br>Energy: 3.106 eV<br>Osc.: 0.076	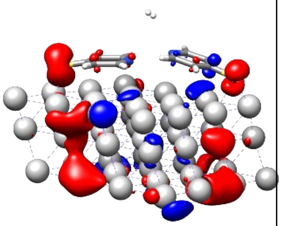<br>State 183<br>Energy: 3.110 eV<br>Osc.: 0.304	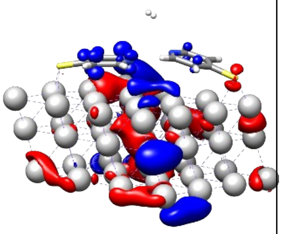<br>State 184<br>Energy: 3.120 eV<br>Osc.: 0.047	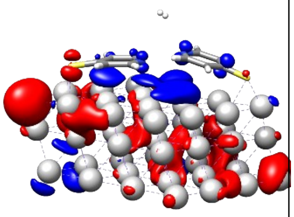<br>State 185<br>Energy: 3.125 eV<br>Osc.: 0.057
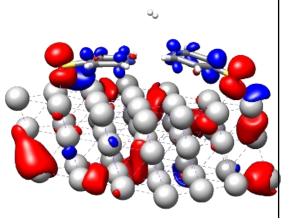<br>State 186<br>Energy: 3.130 eV<br>Osc.: 0.126	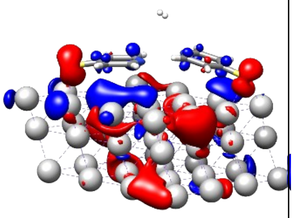<br>State 187<br>Energy: 3.143 eV<br>Osc.: 0.057	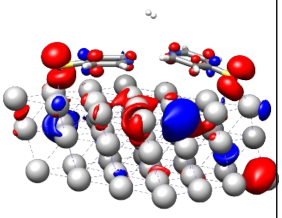<br>State 188<br>Energy: 3.148 eV<br>Osc.: 0.122	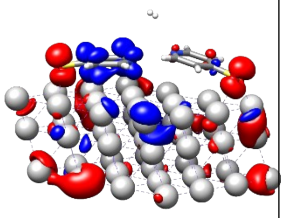<br>State 189<br>Energy: 3.153 eV<br>Osc.: 0.022
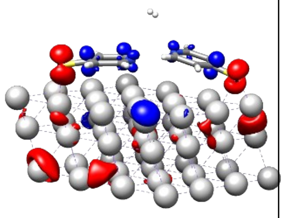<br>State 190<br>Energy: 3.167 eV<br>Osc.: 0.157	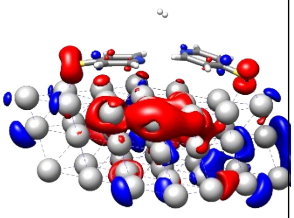<br>State 191<br>Energy: 3.174 eV<br>Osc.: 0.098	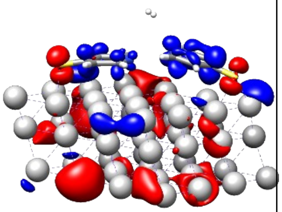<br>State 193<br>Energy: 3.189 eV<br>Osc.: 0.051	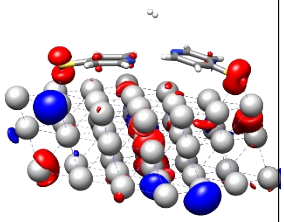<br>State 194<br>Energy: 3.193 eV<br>Osc.: 0.033

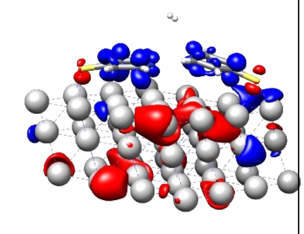

State 195
Energy: 3.203 eV
Osc.: 0.011

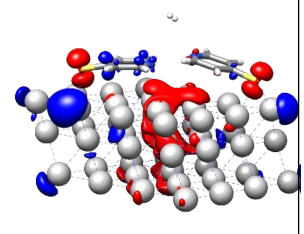

State 197
Energy: 3.211 eV
Osc.: 0.013

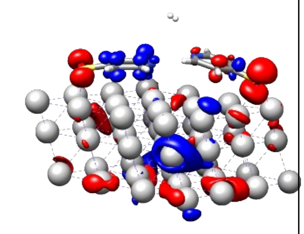

State 198
Energy: 3.225 eV
Osc.: 0.092

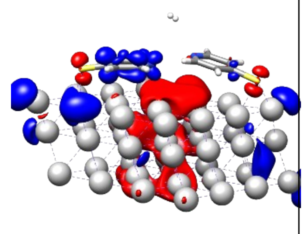

State 199
Energy: 3.228 eV
Osc.: 0.040

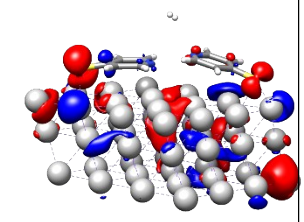

State 200
Energy: 3.237 eV
Osc.: 0.181

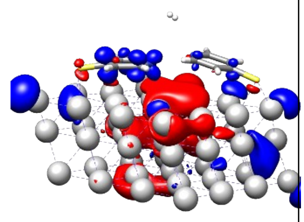

State 201
Energy: 3.238 eV
Osc.: 0.016

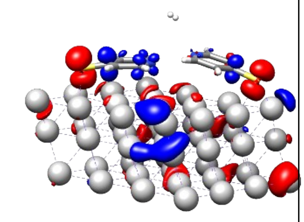

State 202
Energy: 3.247 eV
Osc.: 0.035

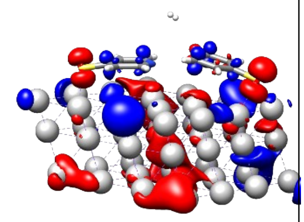

State 203
Energy: 3.255 eV
Osc.: 0.088

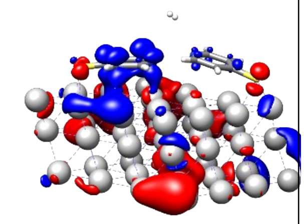

State 205
Energy: 3.268 eV
Osc.: 0.051

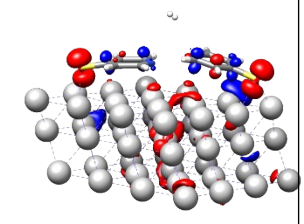

State 206
Energy: 3.276 eV
Osc.: 0.041

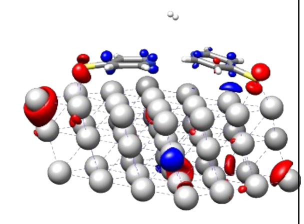

State 207
Energy: 3.285 eV
Osc.: 0.091

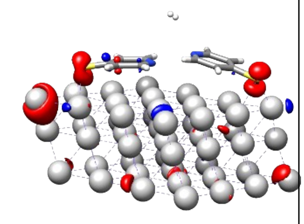

State 208
Energy: 3.286 eV
Osc.: 0.041

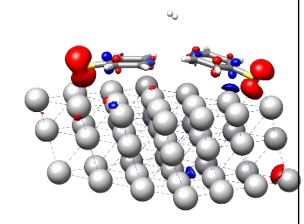

State 209
Energy: 3.289 eV
Osc.: 0.026

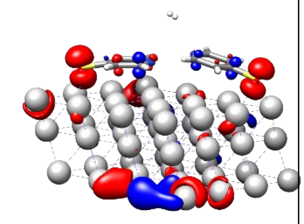

State 210
Energy: 3.293 eV
Osc.: 0.055

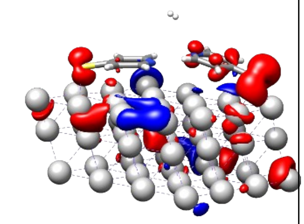

State 211
Energy: 3.304 eV
Osc.: 0.051

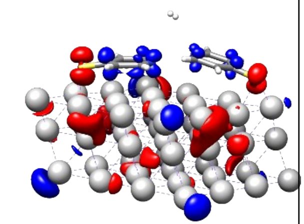

State 212
Energy: 3.305 eV
Osc.: 0.015
